# A prospective analysis of optimal total weight gain ranges and trimester-specific weight gain rates for Chinese pregnant women

**DOI:** 10.1186/s12884-023-05398-8

**Published:** 2023-01-24

**Authors:** Yunhui Gong, Yujie Xu, Ke Wan, Yidi Wang, Linan Zeng, Kun Zou, Yue Chen, Dagang Yang, Jingyuan Xiong, Li Zhao, Lingli Zhang, Toshio Shimokawa, Guo Cheng

**Affiliations:** 1 Department of Gynaecology, West China Women’s and Children’s Hospital, Sichuan University, Chengdu, China; 2 Laboratory of Molecular Translational Medicine, Center for Translational Medicine, Key Laboratory of Birth Defects and Related Diseases of Women and Children (Sichuan University), Ministry of Education, Department of Pediatrics, West China Women’s and Children’s Hospital, Sichuan University, Chengdu, China; 3 Department of Medical Data-science, Graduate School of Medicine, Wakayama Medical University, Wakayama, Japan; 4 West China School of Public Health and West China Fourth Hospital, Sichuan University, Chengdu, China; 5 Department of Pharmacy, Evidence-Based Pharmacy Center, Key Laboratory of Birth Defects and Related Diseases of Women and Children (Sichuan University), West China Women’s and Children’s Hospital, Sichuan University, Chengdu, China; 6 School of Public Health, Southwest Medical University, Luzhou, China; 7 Department of Clinical Nutrition, Affiliated Hospital of Guizhou Medical University, Guiyang, China

**Keywords:** Gestational weight gain, Advanced maternal age, Gestational diabetes mellitus, Preeclampsia, Preterm birth, Cesarean delivery, Large for gestational age, Macrosomia, Small for gestational age, Stillbirth

## Abstract

**Background:**

Gestational weight gain (GWG) criteria recommended by the Institute of Medicine may not be appropriate for Asians. Our aims are to investigate the association between GWG and adverse pregnancy outcomes, and to propose optimal total GWG and rates of GWG for Chinese women.

**Methods:**

Prospective data of 51,125 mother-child pairs from 27 hospitals and community health care centers from Guizhou, Yunnan and Sichuan provinces in China between 2014 and 2018 were analyzed. Generalized Additive Models were performed to determine the associations of GWG with the risk of aggregated adverse outcomes (gestational diabetes mellitus, preeclampsia, cesarean delivery, stillbirth, preterm birth, macrosomia, large for gestational age, and small for gestational age). The range that did not exceed a 2.5% increase from the lowest risk of aggregated adverse outcomes was defined as the optimal GWG range.

**Results:**

Among all participants, U-shaped prospective association was found between GWG and the risk of aggregated adverse pregnancy outcomes. The optimal GWG range of 8.2–13.0 kg was proposed for underweight, 7.3–12.5 kg for normal weight, and 2.0–9.4 kg for overweight/obese women. Meanwhile, a higher GWG rate in the first two trimesters than that in the last trimester was suggested, except for overweight/obese women. After stratified by maternal age, mothers ≥35 years were suggested to gain less weight compared to younger mothers.

**Conclusions:**

To keep a balance between maternal health and neonatal growth, optimal GWG ranges based on Asia-specific BMI categories was suggested for Chinese women with different pre-gravid BMIs and maternal ages.

**Supplementary Information:**

The online version contains supplementary material available at 10.1186/s12884-023-05398-8.

## Background

Excessive or inadequate gestational weight gain (GWG) has been associated with various adverse pregnancy outcomes, including gestational diabetes mellitus (GDM) [1], preeclampsia [2], cesarean delivery [1, 3, 4], stillbirth [5], preterm birth [3, 4], macrosomia [1, 3, 4], large for gestational age (LGA) [3] and small for gestational age (SGA) [3]. The Institute of Medicine (IOM) recommended the optimal GWG range over the course of pregnancy and GWG rate for the last two trimesters in 2009 [6]. However, since the IOM guideline was mainly based on the Caucasian population and standards, its generalizability to Asian women was limited [7–9]. Considering there were approximately 187 million Chinese women of childbearing age (20–39 years) in 2020 [10], establishing an optimal weight gain range for Chinese women is needed.

Several analyses have tried to explore the optimal GWG in Chinese population, yet the limited sample size [11–14] or retrospective design [15, 16] confined the credibility and generalizability. In these investigations, the optimal GWG range is generally proposed either by calculating frequency [12] or by estimating risk [11, 14, 16] of multiple adverse pregnancy outcomes using binary logistic regression, which may lead to misspecification due to purely linear relationships [17]. The generalized additive models (GAMs), as a nonlinear model, will capture the association between risk factors and health outcomes that is not revealed by the binary logistic models, and can avoid model misspecifications in these models [17]. Recently, GAMs were applied to analyze the association between GWG and poor pregnancy outcomes in US [18], and showed high interpretability and predictive accuracy, however, its practice among Chinese pregnant women is lacking. Furthermore, except for optimal total GWG range, the trimester-specific GWG rate is also an important parameter to monitor weight at different stages of pregnancy, but it has not been considered in existing evidence conducted in Chinese women [11–16] or other Asian women [7–9]. It should be noted that in 2021, the Chinese Nutrition Society (CNS) released its GWG guideline [19] and the first report about its applicability among Chinese women has published recently [20]. In this study, we attempted to propose the optimal total and trimester-specific GWG with a cohort of relatively large sample size based on modelling technique GAMs, which may provide a methodological reference for future studies to determine optimal GWG range in their countries, as well as providing evidence for the supplementation of the 2021 CNS guideline in the future.

Moreover, increasing rates of multi-parity and advanced maternal age (AMA) have been detected [21] after the full implementation of a two-child policy of China since 2015 [22]. With the announcement of a three-child policy in 2021 [23], it is likely that more and more Chinese women are giving birth at older ages. However, the impact of GWG on pregnancy outcomes in AMA as well as the optimal GWG for AMA has not been much explored.

Using prospective data from a large cohort, we thus aimed 1) to investigate the association of GWG on multiple maternal and neonatal outcomes including GDM, preeclampsia, cesarean delivery, stillbirth, preterm birth, macrosomia, LGA, and SGA using the GAMs, and 2) to create an optimal total GWG range and trimester-specific GWG range based on Asian-specific body mass index (BMI) categories for Chinese women.

## Methods

### The aim, design and setting of the study

To investigate the impacts of maternal status on the health of mothers and their children, this prospective cohort study was conducted in China between 2014 and 2018. Details on the cohort design are described elsewhere [24]. In brief, singleton pregnant women who had lived without pre-gravid diabetes mellitus or hypertension from 27 hospitals and community health care centers were selected from Guizhou, Yunnan, and Sichuan provinces using a cluster randomized sampling method. Women were followed-up (prenatal care) in regular intervals until they gave birth, and a self-administered questionnaire on socio-demographics, marital and fertile histories was completed once they had been recruited. During prenatal cares, anthropometric measures and clinical examination were routinely conducted as described below and recorded in the medical birth registry. The study was approved by the Ethics Committee of Sichuan University. All participants provided written informed consent for all study content as well as for linkage of their data from the Medical Birth Registry.

Overall, 52,221 pregnant women were recruited from 27 study sites. After exclusion of 1096 participants with missing values of GDM, preeclampsia, stillbirth, and pre-gestational BMI, 51,125 women and their children were included for the final analysis.

### Questionnaire

Detailed maternal information on maternal age, residence (urban/rural), occupation, school education, and personal income at enrollment (gestational week 9–11) were collected by trained investigators. Information on marital (unmarried/married) and fertile (counts of conception and childbirth) histories were also collected. Detailed instruction on collecting height and bodyweight with 3 months before pregnancy were carefully given to participants. Pre-gravid BMI was calculated as weight divided by height squared (kg/m^2^). Specific pre-gravid-BMIs were categorized according to the World Health Organization (WHO) BMI categorization criteria recommended for Asian population [25]: underweight (< 18.5 kg/m^2^), normal weight (18.5–22.9 kg/m^2^), overweight (23–24.9 kg/m^2^), and obese (≥25 kg/m^2^). Overweight and obese categories were combined into one category in this analysis, as limited number of obese participants (2.3%) were recruited [24].

### Anthropometrical measurement

Pre-gravid bodyweight was self-reported, and maternal bodyweight during pregnancy was measured with an ultrasonic meter to the nearest 0.1 kg by medical professionals at registration and at regular intervals (prenatal cares were conducted monthly from recruitment to gestational week 25, every 2 weeks until gestational week 33, and weekly thereafter until birth). Maternal pre-gravid self-reported height was rechecked by medical professionals with electronic stadiometer to the nearest 0.1 cm at the first anthropometric measurement. All anthropometric measurements were performed twice for each participant, which dressed lightly and barefoot during measurements. The overall GWG was calculated by the difference between the bodyweight of a woman at childbirth (the latest measurement of bodyweight) and that within 3 months before the pregnancy. To explore the relevance of GWG on various outcomes in a more precise way, GWG was calculated for each trimester. The first two trimesters were combined as women usually gain a small amount of body weight in the first trimester [26]. Therefore, the weight gain during the first two trimesters was defined as the difference between the weight measured at gestational week 28 and the weight before pregnancy, and GWG in the last trimester was defined as the latest measurement of weight prior to delivery minus the last weight measured at gestational week 28. The weight difference between gestational week 23 and pre-gravid weight was used for GDM risk prediction, as GDM is diagnosed at gestational week 24–28. Pregnant women diagnosed with GDM received dietary treatment followed by insulin treatment for management if the diet alone was insufficient to restore glucose homeostasis. The weekly GWG rate for the first two trimester was calculated using the weight gain during the first two trimesters divided by 28, and the weight gain during the last trimester divided by number of weeks from the gestational week 28 to neonatal delivery was defined as the last trimester GWG rate. For neonates, bodyweight was measured to the nearest 100 g, and recumbent length was assessed to the nearest 0.1 cm with a stadiometer. Scales were checked and calibrated regularly.

### Diagnostic criteria for adverse pregnancy outcomes

Measurements and diagnoses of adverse pregnancy outcomes were performed by professionals in each study center by standard procedure and acknowledged criteria.

Through a two-hour 75 g oral-glucose-tolerance test at gestational week 24–28, patients with GDM were identified if any of the following plasma glucose levels was met: 0 hour (fasting), ≥ 5.1 mmol/L; 1 hour, ≥ 10.0 mmol/L; and 2 hour, ≥ 8.5 mmol/L [27]. Blood pressure was measured by the mercurial blood pressure device. Women with preeclampsia were identified by the following criteria: hypertension (systolic blood pressure ≥ 140 mmHg and/or diastolic blood pressure ≥ 90 mmHg) after 20 weeks of gestation in previously normotensive women, along with the presence of proteinuria [28]. Cesarean deliveries that were performed before or during labor, decided by either doctor or pregnant women, were both included.

Neonates who were delivered earlier than 37 weeks of gestation were defined as preterm delivery [29]. Stillbirth was identified as the death of the fetus after 20 weeks of gestation [30]. Newborn with birthweight of over 4000 g was considered as macrosomia neonate [31]. SGA and LGA were defined as sex- and gestational age-adjusted birth weight < 10th and > 90th percentile, respectively [32].

### Statistical analysis

Mean and standard deviation were expressed for normally distributed continuous variables. Categorical variables were expressed as frequency and proportion. These summary statistics were calculated for all data and each subgroup (normal weight, underweight, and overweight/obese). Statistical models were constructed to estimate the lowest risk at which weight gain causing one of the following adverse events: GDM, preeclampsia, cesarean delivery, stillbirth, preterm delivery, SGA, LGA, and/or macrosomia. GAMs [17] with logit link were used to analyze the relationships between weight gain and adverse pregnancy outcomes, the following function was used for the model’s prediction.

$$\textrm{f}\left(\textrm{x}\right)=\textrm{s}\left(\textrm{Weight}\ \textrm{Gain}\right)+\textrm{s}\left(\textrm{Age}\right)+\left(\textrm{preBMI}\right)+\textrm{s}\left(\textrm{Height}\right)+\textrm{Gender},$$

where s() is smoothed spline function, and “Gender” is dummy function (0: girl, 1: boy). This model was selected based on Akaike’s Information Criteria. Smoothed parameters were calculated using REML method. To estimate the optimal GWG range for Chinese women by keeping a balance between maternal health and fetal growth, the least risky weight gain was calculated using the estimated model by GAMs. The GWG ranges, with respect to the predicted probability, that did not exceed a 2.5% increase from the lowest combined risk of any adverse pregnancy outcomes (the presence of at least one of them: GDM, preeclampsia, cesarean delivery, stillbirth, preterm delivery, SGA, LGA, and macrosomia) were defined as the optimal range in each subgroup of different pre-gravid BMIs or maternal age categories. There is no consensus on the increase criteria to determine the optimal GWG range, and studies used either 1% [7] or 5% [8, 9] increase criteria. Considering that our sample size was in the middle of these studies, 2.5% increases criteria was used. GDM and stillbirth were excluded when estimating the optimal GWG range as these outcomes were identified at gestational week 24–28 and gestational week 20, respectively, medical intervention might be introduced after the occurrence of these outcomes and the association between GWG and the adverse outcomes might not be accurate. Cesarean delivery was excluded since the rate was too high (58.1%). The growth curves for GWG was also constructed using a linear mixed-effects model (fixed effects: gestational weeks, variable effects: subjects). All analyses were performed using the R software, version 3.6.1 (R Development Core Team, Vienna, Austria).

### Quality control

The questionnaire used in this study was reviewed by experts and was then pretested for comprehensibility, acceptability, and logic and flow. The questionnaire was revised on the basis of the results of the pretests. The investigators in each study sites were strictly trained before the formal investigations, including understanding the principles and methods of the investigation and standardizing the interview skills. Physical measurements and clinical examinations were performed by medical professionals using standard methods. All the data were entered by two persons and checked one by one to ensure the accuracy of the entered data.

## Results

### Basic characteristics of study population

The characteristics of the participants are shown in Table 1. Overall, 51,125 mother-child pairs were included with the mean age at 27.6 years and 13.1 kg of GWG on average. 15.3% of pregnant women had underweight pre-gravid BMI and 22.5% were overweight/obese before pregnancy. Around one in five mothers (20.3%) developed GDM, and the prevalence of GDM in overweight/obese mothers (32.1%) was three times higher than that for underweight mothers (11.9%). Children in this study were born after 39.0 weeks of gestation on average, and 1.2% of them suffered from stillbirth and 5.2% were born preterm. The percentage of mothers delivering macrosomia or LGA neonates were higher in overweight/obese mothers than that in underweight mothers, whereas SGA neonates were higher in underweight mothers than overweight/obese mothers. Pre-gravid BMI characteristics of the participants based on maternal age are displayed in Additional file 1: Fig. S1. Most of the participants had a normal weight before pregnancy across all age groups. Notably, the proportion of overweight/obese women increased with maternal age.

**Table 1:** Characteristics^a^ of study participants

Characteristics	Overall	Categories of pre-gravid BMI^b^		
		Normal weight	Underweight	Overweight/obese
Mothers (n (%))	51,125 [100.0]	31,787 [62.2]	7805 [15.3]	11,533 [22.5]
Maternal age (years)	27.6 [4.19]	27.2 [4.08]	25.9 [3.74]	29.6 [4.39]
Primiparous (n (%))	34,876 [68.2]	21,791 [68.6]	5451 [69.8]	7634 [66.2]
Single mother (n (%))	1380 [2.7]	998 [3.1]	197 [2.5]	185 [1.6]
Gestational weight gain (kg)	13.1 [4.17]	13.4 [3.95]	14.1 [3.77]	11.5 [4.55]
GDM (n (%))	10,361 [20.3]	5722 [18.0]	932 [11.9]	3706 [32.1]
Preeclampsia (n (%))	804 [1.6]	368 [1.2]	58 [0.7]	378 [3.3]
Cesarean delivery (n (%))	29,720 [58.1]	18,219 [57.3]	3657 [46.9]	7841 [68.0]
Children				
Gender, girl (n (%))	26,506 [51.9]	16,551 [52.1]	3918 [50.2]	6034 [52.4]
Preterm delivery (n (%))	2645 [5.2]	1514 [4.8]	387 [5.0]	744 [6.5]
Stillbirth (n (%))	588 [1.2]	317 [1.0]	78 [1.0]	193 [1.7]
Gestational age at delivery (wks)	39.0 [1.98]	39.1 [1.89]	39.1 [1.85]	38.8 [2.26]
Birth weight (kg)	3.26 [0.49]	3.26 [0.47]	3.15 [0.45]	3.32 [0.55]
Birth length (cm)	49.4 [2.61]	49.5 [2.48]	49.2 [2.50]	49.4 [3.01]
Macrosomia (n (%))	2104 [4.1]	1186 [3.7]	119 [1.5]	799 [6.9]
Small for gestational age (n (%))	677 [1.3]	405 [1.3]	147 [1.9]	125 [1.1]
Large for gestational age (n (%))	554 [1.1]	310 [1.0]	30 [0.4]	214 [1.9]

^a^ Values are means (SD) or frequencies

^b^ Categorized by WHO Asian [25]

*BMI* body mass index, *GDM* gestational diabetes mellites

### Association between GWG and pregnancy outcomes

The association between GWG and the combination of eight aggregated adverse pregnancy outcomes at each BMI category is displayed in Table 2. A U-shaped association between GWG and predicted probability of composite adverse outcomes was observed. Compared to pregnant women who gained 13.0 kg (mean GWG), those gained 5.0 kg (low level among participants) or 20.0 kg (high level among participants) had higher risks of aggregated adverse pregnancy outcomes for all BMI categories. The association between GWG and the predicted probability of each adverse outcome was also examined (Additional file 2**:** Table S1 and Table S2). For neonatal outcomes, the predicted probabilities of macrosomia and LGA increased whereas the predicted probability of SGA decreased as weight gain increased at each BMI. Compared to pregnant women who gained 20.0 kg, those gained 5.0 kg or 13.0 kg weight had lower predicted probabilities of GDM, preeclampsia and cesarean delivery for all BMI categories.

**Table 2:** GWG^a^ and predicted probability of aggregated adverse pregnancy outcomes stratified by pre-gravid BMI in 51,125 mothers

GWG	Predicted Probability		
	Underweight (*n* = 7805)	Normal weight (*n* = 31,787)	Overweight/obese (*n* = 11,533)
Lower 95% CI of GWG^b^	0.452 [0.414–0.491]	0.576 [0.557–0.594]	0.740 [0.720–0.759]
Mean GWG^c^	0.341 [0.321–0.361]	0.449 [0.437–0.461]	0.688 [0.669–0.706]
Upper 95% CI GWG^d^	0.370 [0.341–0.399]	0.501 [0.483–0.519]	0.711 [0.681–0.739]

^a^ Predicted probability of GWG on GDM was calculated from initial pregnant to 24wks of gestation, since medical intervened in pregnancy once gravid diagnosed GDM

^b^ Lower 95% CI of GWG, 97.5% mother gained over 5.0 kg gestational weight during pregnancy

^c^ Mean GWG, the mean GWG for participants is 13.0 kg

^d^ Upper 95% CI of GWG, 97.5% mother gained less than 20.0 kg gestational weight during pregnancy

*GWG* gestational weight gain, *BMI* body mass index, *WHO* World Health Organization, *CI* confidence interval

The predicted probability of each adverse pregnancy outcome and of the aggregated adverse pregnancy outcome according to GWG by pre-gravid BMI, maternal age and trimester is displayed in Additional file 3. Overall, a rising predicted probability of all adverse pregnancy outcomes was observed with a higher maternal age. The effect of GWG on adverse pregnancy outcomes was varied among trimesters.

### Determination of optimal GWG range

**Fig. 1:**
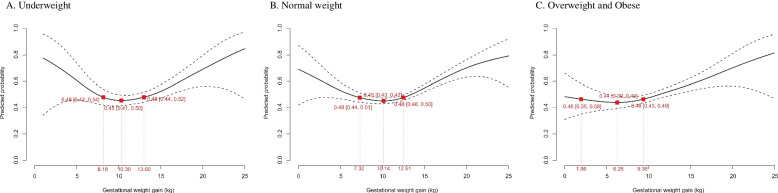
Optimal gestational weight gain for Chinese pregnant women. The predicted probability that did not exceed a 2.5% increase from the lowest aggregated risk of adverse pregnancy outcomes (preeclampsia, preterm delivery, small for gestational age, large for gestational age, and/or macrosomia) was 0.45–0.48 for underweight women, 0.45–0.48 for normal weight women, and 0.44–0.46 for overweight/obese women, which corresponded to 8.2–13.0 kg, 7.3–12.5 kg, and 2.0–9.4 kg of total gestational weight gain, respectively

The optimal GWG, which was proposed by the least risky range for Chinese women developing adverse pregnancy outcomes categorized by pre-gravid BMI, is presented in Fig. 1. Based on the lowest predicted probability of aggregated adverse pregnancy outcomes (0.45–0.48, 0.45–0.48 and 0.44–0.46 for underweight, normal weight and overweight/obese women, respectively), overweight/obese women were recommended to gain the least amount of weight of 6.3 (2.0, 9.4) kg during pregnancy, and normal weight and underweight women were suggested to gain 10.1 (7.3, 12.5) kg and 10.3 (8.2, 13.0) kg, respectively. The growth curve for the suggested weight gain was also estimated and is shown in Additional file 1: Fig. S2.

Table 3 displays the optimal rate of GWG for Chinese pregnant women. For underweight and normal weight women, rate of GWG in the third trimester was slower (32.1 and 20.8% respectively) than that in the first two trimesters, whereas for overweight/obese women, no weight gain was suggested in the first two trimesters.

**Table 3:** Optimal gestational weight gain for Chinese pregnant women

	Maternal pre-gravid BMI category^a^			Overall
	Underweight (*n* = 7805)	Normal weight (*n* = 31,787)	Overweight/obese^b^ (*n* = 11,533)	
Whole pregnancy				
Total weight gain (kg)	10.30 [8.18, 13.00]	10.14 [7.32, 12.51]	6.26 [1.96, 9.38]	9.31 [6.22, 12.25]
1st-2nd trimester				
Total weight gain (kg)	7.36 [5.32, 9.94]	6.36 [4.32, 8.35]	–	5.92 [2.16, 8.44]
Rate of weight gain (kg/w)	0.28 [0.20, 0.38]	0.24 [0.17, 0.32]	–	0.23 [0.08, 0.32]
3rd trimester				
Total weight gain (kg)	2.68 [0.91, 4.46]	2.61 [1.42, 5.30]	2.00 [1.05, 3.49]	2.28 [1.20, 4.88]
Rate of weight gain (kg/w)	0.19 [0.07, 0.32]	0.19 [0.10, 0.38]	0.14 [0.08, 0.25]	0.16 [0.09, 0.35]

^a^ Categorized by WHO Asian [25]

^b^ The curve of impact of GWG in the first two trimesters on adverse pregnancy outcome among pre-gravid overweight or obese women was almost linear, without the lowest risky value which is required to obtain the optimal GWG range, thus an optimal GWG range in this subgroup was inaccessible

*BMI* body mass index

Additional file 2: Table S3 shows the optimal GWG for Chinese women at differed maternal ages. Mothers older than 35 years were recommended to gain 1.4 kg less weight during the first two trimesters, 0.6 kg less weight during the last trimester, and 4.1 kg less weight during the whole pregnancy than mothers younger than 25 years. Due to the limited sample size of subpopulations with different pre-gravid BMIs and maternal ages, the pre-gravid BMI-specific optimal GWG at different maternal ages is not presented.

## Discussion

In this cohort, we figured out a U-shaped prospective association between GWG and the risk of aggregated adverse pregnancy outcomes among 51,125 participants. Based on these associations, an optimal GWG range was suggested as 8.2–13.0 kg, 7.3–12.5 kg, and 2.0–9.4 kg respectively for underweight, normal weight and overweight/obese Chinese women, and a higher rate of GWG in the first two trimesters than the last trimester was suggested.

During gestation, pregnant women and their children are, to varying degree, simultaneously at risk of multiple adverse health outcomes. Our finding of the U-shaped association between GWG and total risk of combined adverse maternal and neonatal outcomes was in line with previous analyses [7–9, 11], which implies the importance of proper GWG. Thus, proposing an optimal GWG range specific for Chinese women to keep the balance between maternal health and neonatal growth is crucial.

Based on prospective data from 51,125 mother-child pairs, we suggested the optimal GWG ranges for Chinese pregnant women, which were generally lower than that recommended by the IOM, for all pre-gravid BMIs. Different from the WHO BMI categories applied in the IOM, the GWG range established in our analysis was based on the Asia-specific BMI categories, which are more proper recommendations for Chinese women. From the aspect of racial or ethnic differences, Asian populations have lower BMI levels but higher body fat levels than Caucasians [33] leading to their different susceptibility to weight gain in pregnancy. Moreover, evidence has shown that Asians have increased risk of obesity-related diseases at lower BMI levels than Caucasians [34, 35], therefore, lower BMI cut-off utilized and lower GWG suggested in the present study should be safer for Asian populations. Besides, various recommendations of GWG for different populations were proposed in Japan [7], Korea [8], Singapore [9], German [36], Belgian [37] and US [18], which implies that the optimal GWG may be population-specific, and each country should consider its own optimal GWG range. Furthermore, compared to several studies in China with small sample size [11–14] or retrospective design [15, 16], the present study was a large, prospective cohort, and the characteristics of the participants (e.g., mean maternal age, the prevalence of underweight or overweight/obese in women, and birth weight) were similar to the data from National Statistical Yearbook [38, 39] and national-wide surveys [32, 40]. Notably, the national GWG guideline for Chinese women has been published in 2021 [19]. The suggested GWG range in our study was slightly lower than the one recommended by the CNS, possibly due to the fact that the methodology was varied.

The 2009 IOM guideline [6] has recommended the weekly GWG rate over the course of the 2nd and 3rd trimesters, and so far, only one US study [18] has tried to analyze it. Actually, only 10–13% of Chinese women gained their weight within the IOM guideline in the last two trimesters [41], and the pattern of GWG would be influenced by maternal ethnicity [6], thus the GWG rate for Caucasians was not appropriate for Chinese women. In contrast to IOM [6] and the US study [18], we suggested a higher GWG rate in the first two trimesters than that in the last trimester, except for pre-gravid overweight/obese women. Our findings may have important public health implications, for providing specific guidance for pregnant women to track their weight over trimesters and achieve the recommend total GWG. Maintaining appropriate weight gain at different trimester is a key to prevent pregnancy complications and improve well-being of mothers and their children, given the impacts of GWG on pregnancy outcomes during various trimesters were observed in the present study.

There is an increasing trend of delayed childbearing in China, given the fertility rate of women aged 30–34 years increased from 5.7‰ in 1995 to 18.6‰ in 2015, whereas the rate declined for younger women [38, 39]. Furthermore, there was a 7.2% monthly increase in multiparous births of mothers older than 35 years [21] since the implementation of the two-child policy in 2015. Considering the alarming rise of older mothers, and they were at higher risks of pregnancy complications as shown in our study, a first attempt on the maternal age-specific optimal GWG range was made. We suggested a lower overall GWG for mothers older than 35 years compared to younger mothers. This might be partially because the proportion of women being overweight/obese before pregnancy increased with maternal age, given the pre-gravid overweight/obesity is a risk factor for multiple adverse pregnancy outcomes [24] and a lower amount of GWG was generally recommended for overweight/obese women [7, 11, 12, 18, 36, 37]. In order to better understand the association between maternal overweight/obesity and AMA, further research on optimal GWG for AMA at different pre-gravid BMIs is warranted.

Our study has several strengths. Our participants and their children were representative of the general population in age, distribution of pre-gravid BMI and birth weight according to National Statistical Yearbooks [38, 39] and national surveys [32]. The prospective nature and large sample size, in conjunction with the ability to consider for a broad range of pre-gravid BMI and maternal age categories of participants represent substantial strengths. Another advantage lies in the use of the non-linear statistical model to provide a more credible reference of optimal GWG. Notably, the maternal age specific GWG reference was considered in our analysis, which might provide new idea on prenatal care of women of AMA, on account of the rising prevalence of this population. The results may also stimulate future investigations to supplement the 2021 CNS guideline by considering different characteristics of population. Limitations are still worth noted. The current study only presented the preliminary results on the optimal GWG for AMA due to the limited sample size of subpopulations, further details with respect to different pre-gravid BMI or parity are needed. GWG and GWG rate in the first two trimesters were combined in this study as women usually gain limited amount of weight during the first trimester. However, the combined GWG rate may mask the effect of individual trimester GWG rate, and future research on optimal weight gain at each trimester is warranted. While the study population was representative of the general population in age, pre-gravid BMI and birth weight, they were recruited from the Southwest China, and the generalizability of the findings to the Chinese population need further investigation.

## Conclusions

In conclusion, optimal GWG ranges of 8.2–13.0 kg, 7.3–12.5 kg and 2.0–9.4 kg were indicated respectively for underweight, normal weight and overweight/obese Chinese women by Asian-specific BMI categories, which was proposed to keep a balance between maternal health and neonatal growth.

## Supplementary Information

**Additional file 1: Figure S1.** The age category specified maternal pre-gravid BMI distributionaccording to WHO classifications for Asian population **Figure S2.** The growth curve for gestational weight gain. The brown area represents GWG growth curves for normal-weight mothers reported in the prospective multi-country study by Ismail et al. (PMID: 26926301). The green area represents the IOM recommendation.

**Additional file 2: Table S1.** Separated predicted probability of whole pregnant GWG on neonatal outcomes in 51,125 offspring. **Table S2** Separated predicted probability of whole pregnant GWG on maternal outcomes in 51,125 mothers. Table S3 Optimal gestational weight gain for Chinese pregnant women at different maternal ages.

**Additional file 3.** Predicted probability of each adverse pregnancy outcome and of aggregated adverse pregnancy outcome with increasing GWG by pre-gravid BMIs, maternal ages and trimesters.

## Data Availability

The datasets used and/or analyzed during the current study are available from the corresponding author on reasonable request.

## Abbreviations

AMA: Advanced maternal age
BMI: Body mass index
CI: confidence interval
GAM: Generalized additive model
GDM: Gestational diabetes mellitus
GWG: Gestational weight gain
IOM: Institute of Medicine
LGA: Large for gestational age
SGA: Small for gestational age
WHO: World Health Organization
