# Supplementary material for: A prospective analysis of optimal total weight gain ranges and trimester-specific weight gain rates for Chinese pregnant women
Source: BMC Pregnancy Childbirth. 2023 Jan 24;23:60. doi: 10.1186/s12884-023-05398-8 (PMC9872325; doi:10.1186/s12884-023-05398-8)
Supplement: Supplementary file 1 — Additional file 1: Figure S1. The age category specified maternal pre-gravid BMI distributionaccording to WHO classifications for Asian population Figure S2. The growth curve for gestational weight gain. The brown area represents GWG growth curves for normal-weight mothers reported in the prospective multi-country study by Ismail et al. (PMID: 26926301). The green area represents the IOM recommendation. [file 12884_2023_5398_MOESM1_ESM.pdf]

Additional file 1: Figure S1. The age category specified maternal pre-gravid BMI distribution according to WHO classifications for Asian population

Figure S2. The growth curve for gestational weight gain

(PDF 143KB)

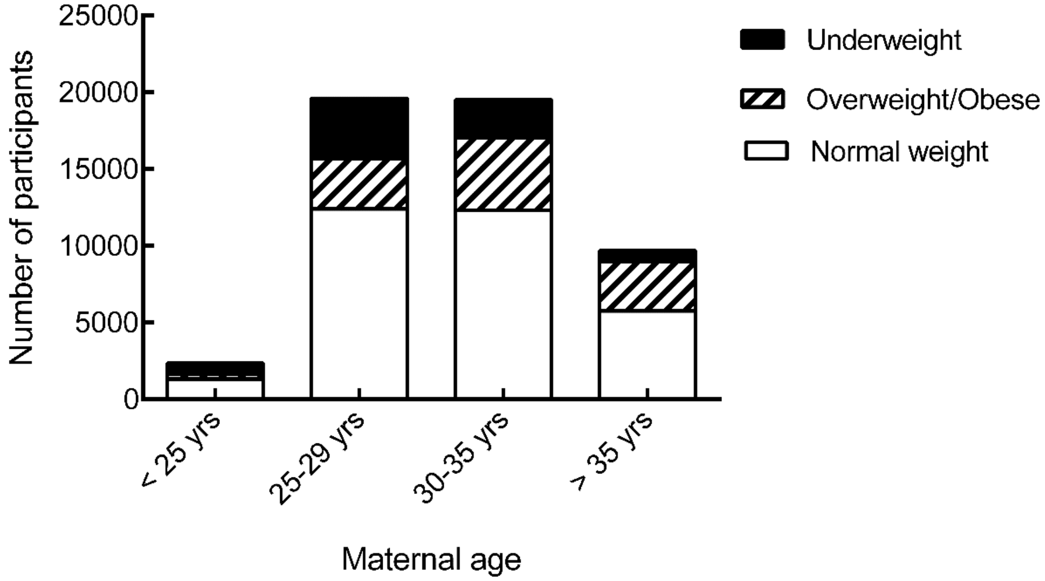

Figure S1. The age category specified maternal pre-gravid BMI distribution according to WHO classifications for Asian population

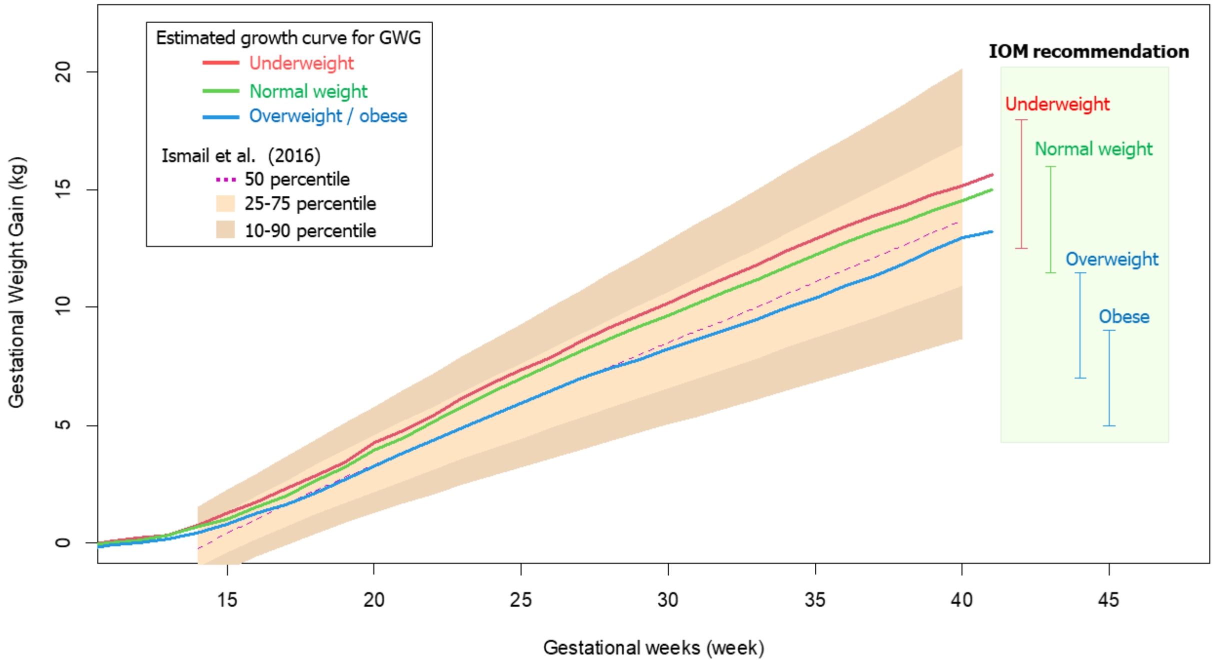

Figure S2. The growth curve for gestational weight gain

The brown area represents GWG growth curves for normal-weight mothers reported in the prospective multi-country study by Ismail et al (PMID: 26926301). The green area represents the IOM recommendation
