# Supplementary material for: A prospective analysis of optimal total weight gain ranges and trimester-specific weight gain rates for Chinese pregnant women
Source: BMC Pregnancy Childbirth. 2023 Jan 24;23:60. doi: 10.1186/s12884-023-05398-8 (PMC9872325; doi:10.1186/s12884-023-05398-8)
Supplement: Supplementary file 2 — Additional file 2: Table S1. Separated predicted probability of whole pregnant GWG on neonatal outcomes in 51,125 offspring. Table S2 Separated predicted probability of whole pregnant GWG on maternal outcomes in 51,125 mothers. Table S3 Optimal gestational weight gain for Chinese pregnant women at different maternal ages. [file 12884_2023_5398_MOESM2_ESM.pdf]

Additional file 2: Table S1. Separated predicted probability of whole pregnant GWG on neonatal outcomes in 51,125 offspring.

Table S2. Separated predicted probability of whole pregnant GWG on maternal outcomes in 51,125 mothers.

Table S3. Optimal gestational weight gain for Chinese pregnant women at different maternal ages.

(PDF 127 KB)

Table S1 Separated predicted probability of whole pregnant GWG on neonatal outcomes in 51,125 offspring

	Maternal Pre-gravid BMI category according to WHO Asian		
	Underweight (n=7805)	Normal weight (n=31787)	Overweight/obese (n=11533)
Stillbirth			
Lower 95% CI of GWG[a]	0.002 [0.000 - 0.007]	0.005 [0.003 - 0.010]	0.010 [0.005 - 0.019]
Mean GWG[b]	0.003 [0.001 - 0.006]	0.003 [0.002 - 0.005]	0.009 [0.006 - 0.015]
Upper 95% CI GWG[c]	0.005 [0.002 - 0.011]	0.002 [0.001 - 0.004]	0.010 [0.004 - 0.024]
Preterm delivery			
Lower 95% CI of GWG	0.132 [0.104 - 0.166]	0.136 [0.121 - 0.153]	0.225 [0.197 - 0.255]
Mean GWG	0.085 [0.073 - 0.098]	0.099 [0.092 - 0.108]	0.199 [0.177 - 0.223]
Upper 95% CI GWG	0.093 [0.076 - 0.113]	0.109 [0.097 - 0.122]	0.240 [0.202 - 0.283]
Macrosomia			
Lower 95% CI of GWG	0.007 [0.004 - 0.011]	0.051 [0.044 - 0.059]	0.216 [0.193 - 0.241]
Mean GWG	0.022 [0.017 - 0.029]	0.095 [0.089 - 0.102]	0.296 [0.278 - 0.315]
Upper 95% CI GWG	0.069 [0.057 - 0.083]	0.176 [0.164 - 0.188]	0.392 [0.358 - 0.428]
Large for gestational age			
Lower 95% CI of GWG	0.073 [0.061 - 0.089]	0.212 [0.195 - 0.230]	0.488 [0.461 - 0.514]
Mean GWG	0.135 [0.123 - 0.148]	0.266 [0.255 - 0.277]	0.542 [0.521 - 0.562]
Upper 95% CI GWG	0.236 [0.216 - 0.258]	0.387 [0.370 - 0.404]	0.622 [0.591 - 0.653]
Small for gestational age			
Lower 95% CI of GWG	0.199 [0.167 - 0.235]	0.163 [0.147 - 0.181]	0.119 [0.100 - 0.143]
Mean GWG	0.115 [0.102 - 0.129]	0.082 [0.075 - 0.089]	0.086 [0.073 - 0.101]
Upper 95% CI GWG	0.082 [0.067 - 0.100]	0.056 [0.048 - 0.066]	0.066 [0.048 - 0.091]

[a] Lower 95% CI of GWG, 97.5% mother gained over 5.0 kg gestational weight during pregnancy

[b] Mean GWG, the mean GWG for participants is 13.0 kg

[c] Higher 95% CI of GWG, 97.5% mother gained less than 20.0 kg gestational weight during pregnancy

GWG gestational weight gain, *BMI* body mass index, *WHO* World Health Organization, *CI* confidence interval

Table S2 Separated predicted probability of whole pregnant GWG on maternal outcomes in 51,125 mothers

	Pre-gravid BMI category according to WHO Asian		
	Underweight (n=7805)	Normal weight (n=31787)	Overweight/obese (n=11533)
GDM[a]			
Lower 95% CI of GWG[b]	0.221 [0.196 - 0.247]	0.330 [0.316 - 0.344]	0.607 [0.589 - 0.626]
Mean GWG[c]	0.214 [0.197 - 0.232]	0.345 [0.334 - 0.356]	0.620 [0.605 - 0.636]
Upper 95% CI GWG[d]	0.230 [0.206 - 0.256]	0.385 [0.369 - 0.401]	0.633 [0.607 - 0.659]
preeclampsia			
Lower 95% CI of GWG	0.012 [0.006 - 0.025]	0.027 [0.020 - 0.035]	0.129 [0.107 - 0.156]
Mean GWG	0.014 [0.010 - 0.020]	0.027 [0.023 - 0.032]	0.129 [0.110 - 0.151]
Upper 95% CI GWG	0.025 [0.017 - 0.036]	0.048 [0.040 - 0.058]	0.211 [0.173 - 0.255]
Cesarean delivery			
Lower 95% CI of GWG	0.513 [0.482 - 0.543]	0.660 [0.646 - 0.674]	0.794 [0.779 - 0.808]
Mean GWG	0.528 [0.513 - 0.543]	0.634 [0.625 - 0.643]	0.783 [0.771 - 0.795]
Upper 95% CI GWG	0.566 [0.546 - 0.587]	0.675 [0.662 - 0.687]	0.800 [0.780 - 0.818]

[a] Predicted probability of GWG on GDM was calculated from initial pregnant to 24wks of gestation, since medical intervened in pregnancy once gravid diagnosed GDM.

[b] Lower 95% CI of GWG, 97.5% mother gained over 5.0 kg gestational weight during pregnancy;

[c] Mean GWG, the mean GWG for participants is 13.0 kg:

[d] Lower 95% CI of GWG, 97.5% mother gained less than 20.0 kg gestational weight during pregnancy.

GWG gestational weight gain, *BMI* body mass index, *WHO* World Health Organization, *CI* confidence interval

Table S3 Optimal gestational weight gain for Chinese pregnant women of different maternal ages

	Maternal age			
	<25 yrs (n=2336)	25-29 yrs (n=19601)	30-35 yrs (n=19513)	≥35 yrs (n=9675)
1st-2nd trimester				
Total weight gain (kg)	7.54 [2.92, 11.26]	6.01 [0.59, 8.90]	5.32 [1.14, 8.13]	6.16 [2.81, 8.35]
Rate of weight gain (kg/w)	0.29 [0.11, 0.43]	0.23 [0.02, 0.34]	0.20 [0.04, 0.31]	0.24 [0.11, 0.32]
3rd trimester				
Total weight gain (kg)	2.99 [1.91, 4.38]	2.34 [1.05, 5.41]	3.00 [1.51, 5.11]	2.37 [1.18, 3.88]
Rate of weight gain (kg/w)	0.21 [0.14, 0.31]	0.17 [0.08, 0.39]	0.21 [0.11, 0.37]	0.17 [0.08, 0.28]
Whole pregnancy				
Total weight gain (kg)	12.77 [10.53, 14.67]	9.70 [5.88. 12.71]	9.28 [6.34, 12.12]	8.63 [6.09, 11.16]
Rate of weight gain (kg/w)	0.23 [0.26, 0.37]	0.24 [0.15, 0.32]	0.23 [0.16, 0.30]	0.22 [0.16, 0.28]
