# Supplementary material for: A prospective analysis of optimal total weight gain ranges and trimester-specific weight gain rates for Chinese pregnant women
Source: BMC Pregnancy Childbirth. 2023 Jan 24;23:60. doi: 10.1186/s12884-023-05398-8 (PMC9872325; doi:10.1186/s12884-023-05398-8)
Supplement: Supplementary file 3 — Additional file 3. Predicted probability of each adverse pregnancy outcome and of aggregated adverse pregnancy outcome with increasing GWG by pre-gravid BMIs, maternal ages and trimesters. [file 12884_2023_5398_MOESM3_ESM.pdf]

Additional file 3: Predicted probability of each adverse pregnancy outcome and of aggregated adverse pregnancy outcome with increasing GWG by pre-gravid BMIs, maternal ages and trimesters. (PDF 1MB)

Statistical Analysis Report
Toshio SHIMOKAWA (Wakayama Medical University)

1. Evaluation of weight gain between pre and 24 weeks after pregnancy

- Generalized additive model (GAM) was used for predictive model of each disease.
- The above statistical model was applied to subsets of each age and BMI (ASIA).
- Reference weight gain = 5kg (Mean weight gain of mothers without any disease).

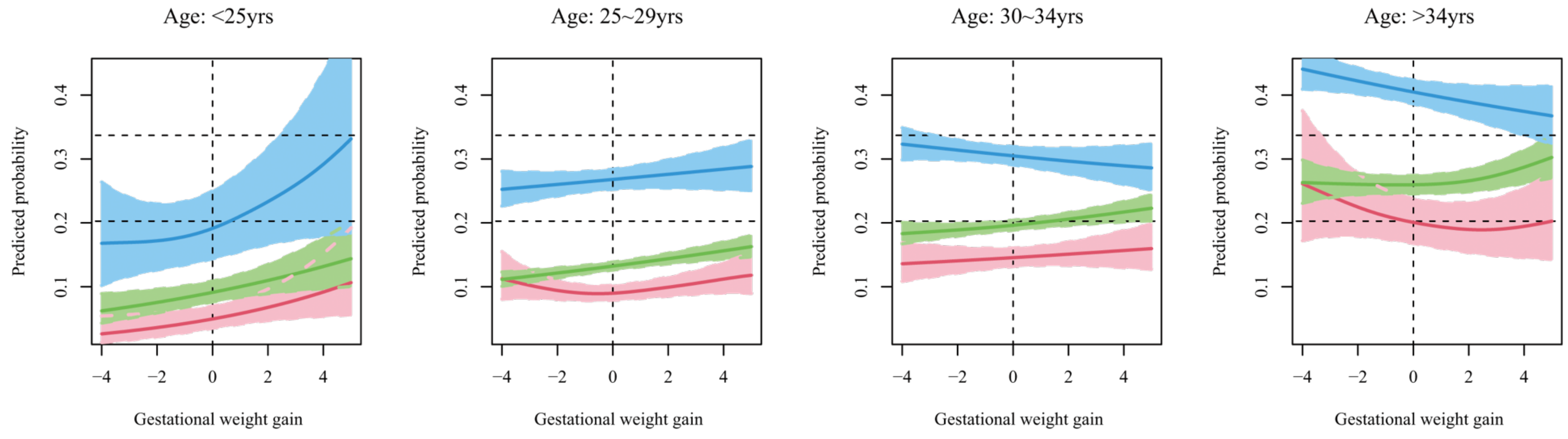

(a) **Gestational diabetes mellitus** (Red: Normal weight, Green: Underweight, Blue: Overweight/obese)

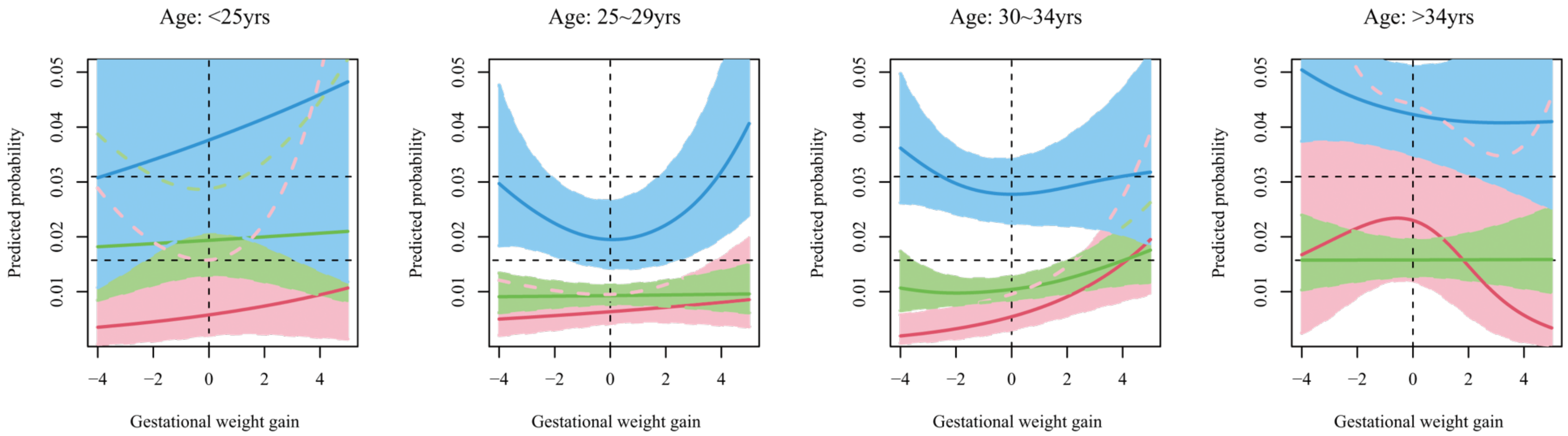

(b) **Preeclampsia** (Red: Normal weight, Green: Underweight, Blue: Overweight/obese)

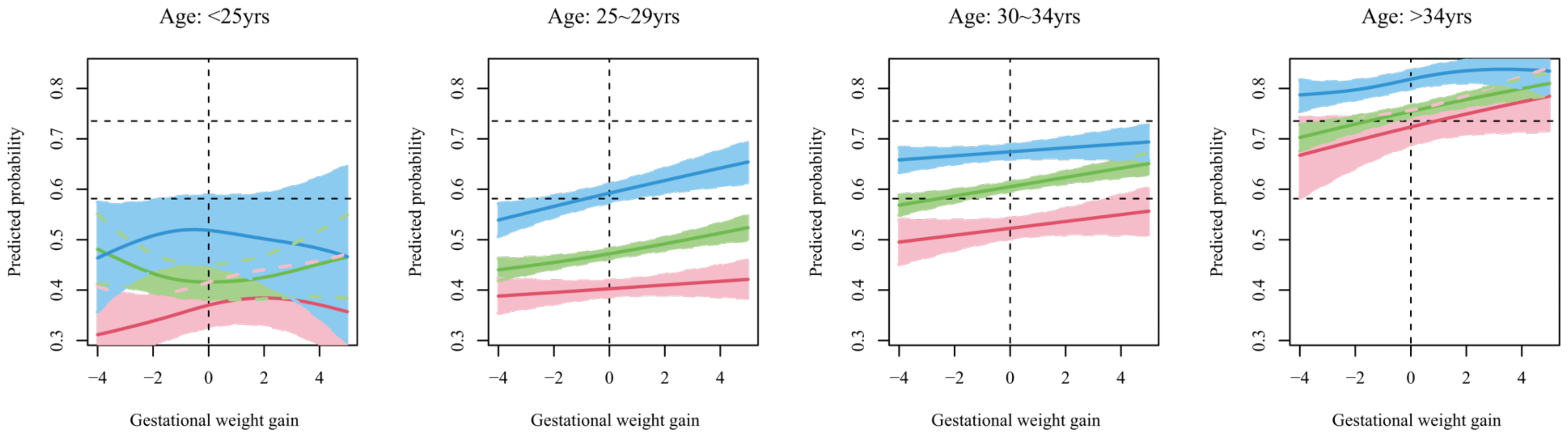

(c) **Cesarean delivery** (Red: Normal weight, Green: Underweight, Blue: Overweight/obese)

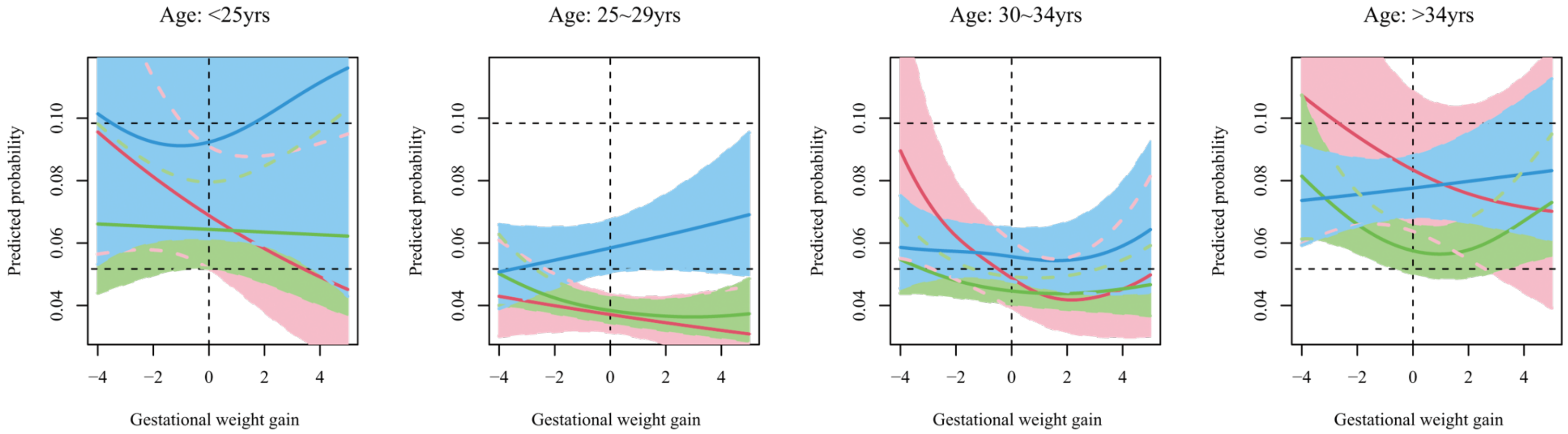

(d) **Preterm delivery** (Red: Normal weight, Green: Underweight, Blue: Overweight/obese)

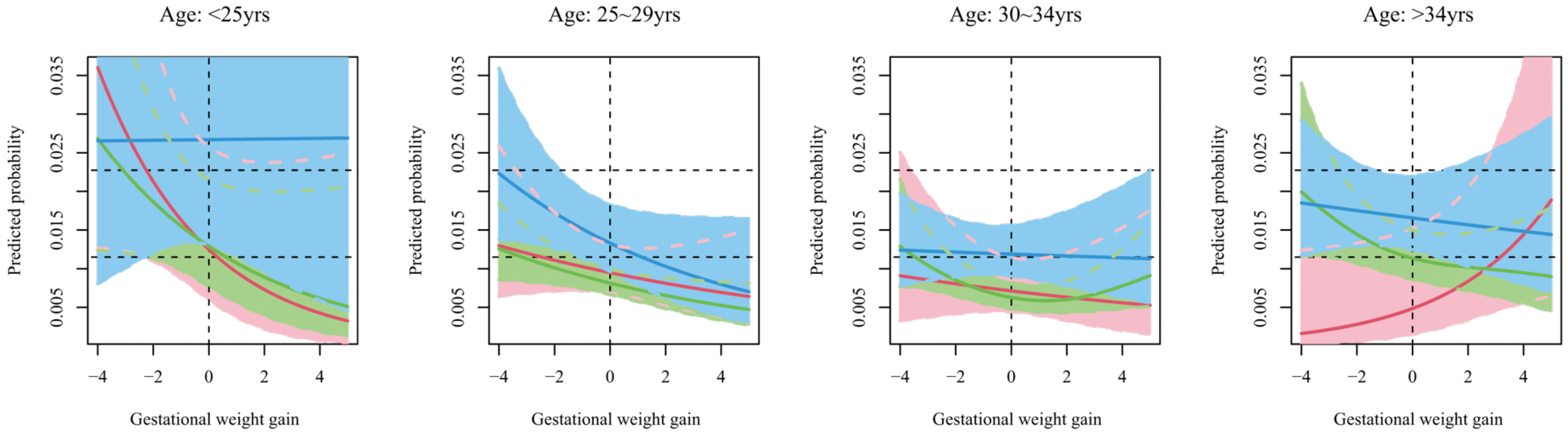

(e) **Stillbirth** (Red: Normal weight, Green: Underweight, Blue: Overweight/obese)

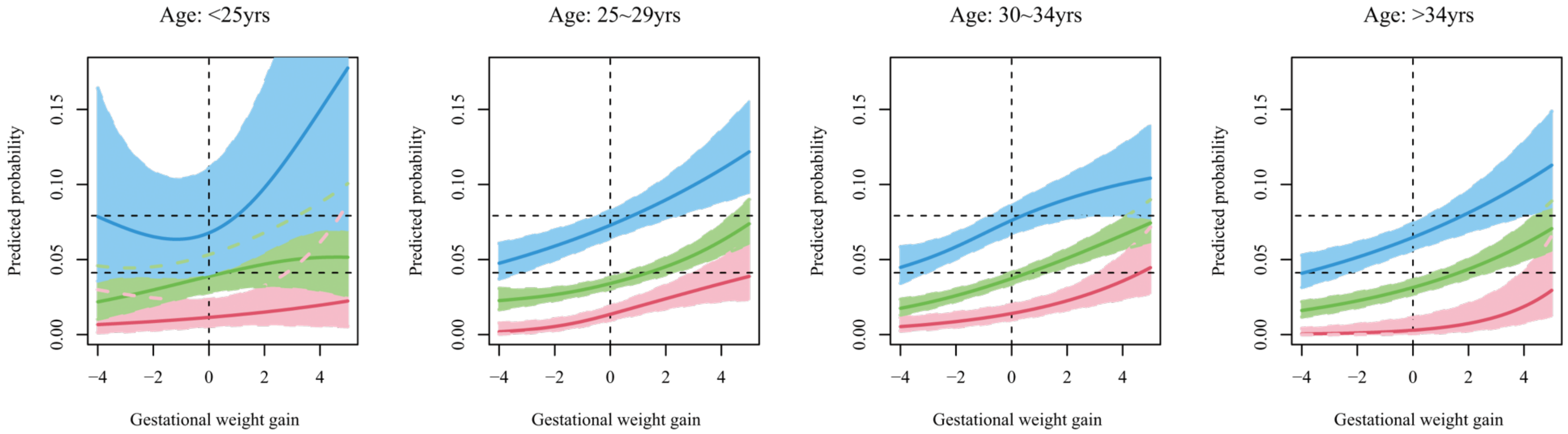

(f) **Macrosomia** (Red: Normal weight, Green: Underweight, Blue: Overweight/obese)

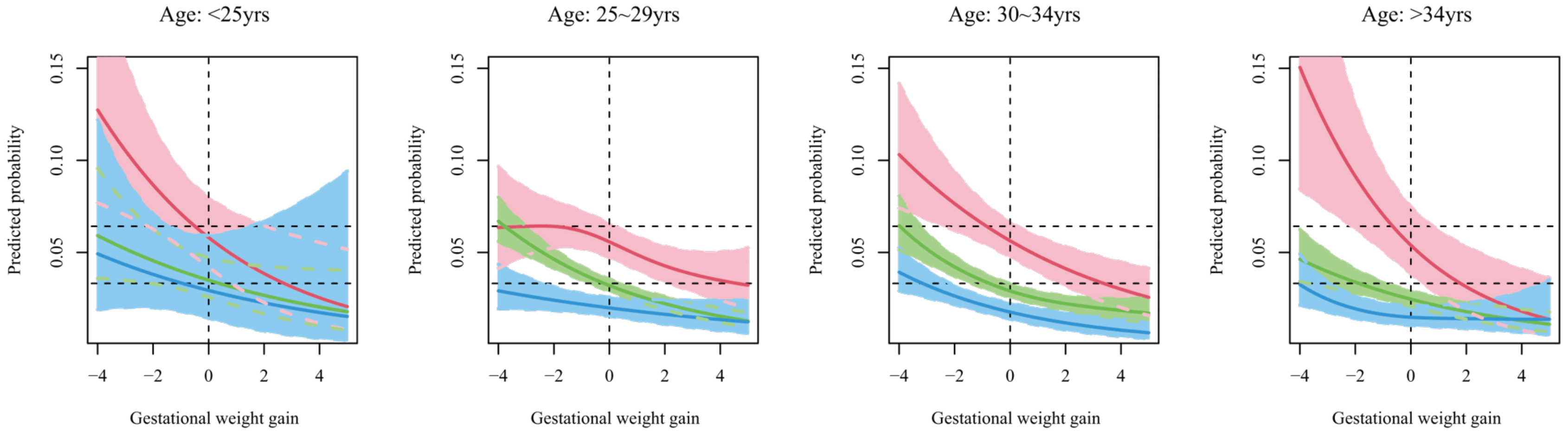

(g) **Small for gestational age** (Red: Normal weight, Green: Underweight, Blue: Overweight/obese)

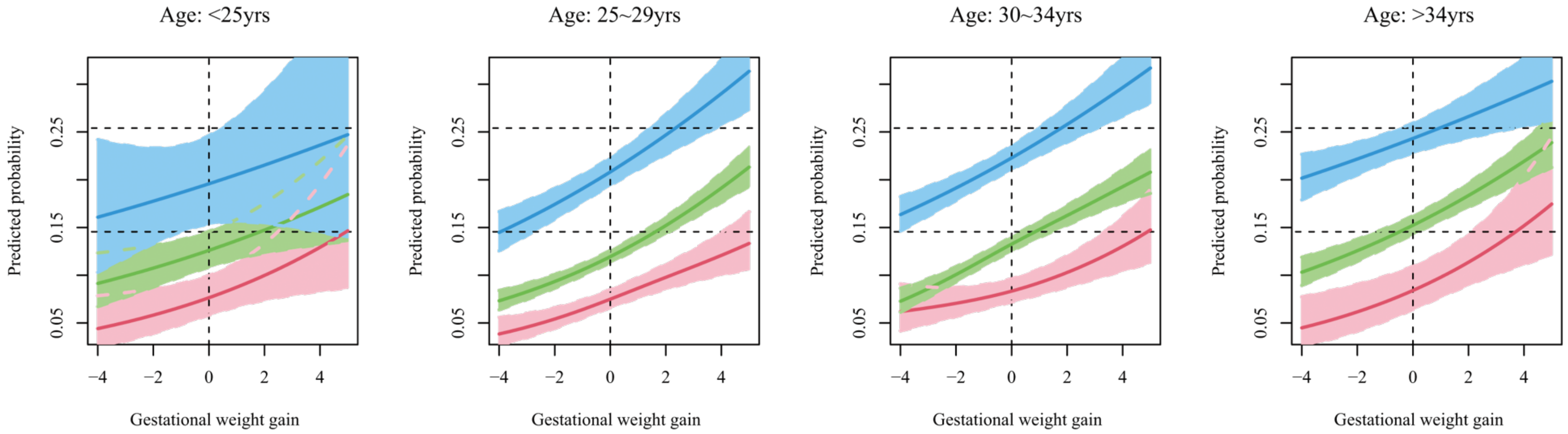

(h) **Large for gestational age** (Red: Normal weight, Green: Underweight, Blue: Overweight/obese)

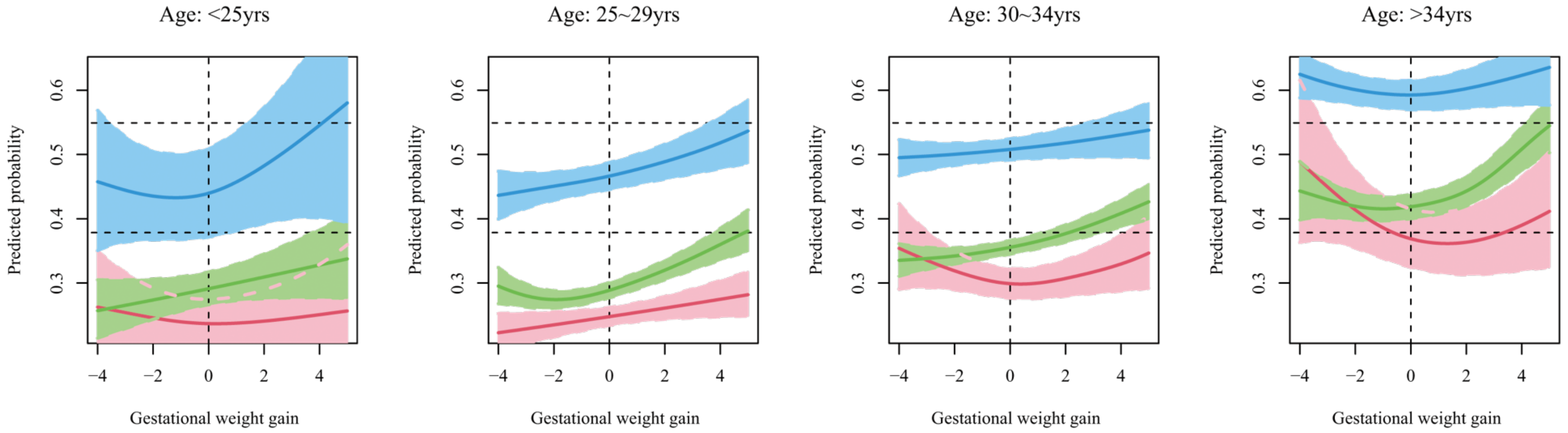

(i) **All disease** (Red: Normal weight, Green: Underweight, Blue: Overweight/obese)

1.1

Gestational diabetes mellitus

Age		<25 yrs			25-29 yrs	
Weight gain	Normal weight (n=1,324)	Underweight (n=698)	Overweight/Obese (n=314)	Normal weight (n=12,406)	Underweight (n=3,943)	Overweight/Obese (n=3,249)
-5.	0.022 [0.009 - 0.052]	0.056 [0.037 - 0.086]	0.164 [0.083 - 0.298]	0.125 [0.076 - 0.198]	0.107 [0.095 - 0.120]	0.249 [0.218 - 0.282]
-4.5	0.024 [0.011 - 0.053]	0.059 [0.040 - 0.088]	0.166 [0.093 - 0.278]	0.119 [0.079 - 0.175]	0.109 [0.098 - 0.122]	0.251 [0.222 - 0.281]
-4	0.026 [0.012 - 0.054]	0.062 [0.043 - 0.089]	0.167 [0.102 - 0.262]	0.113 [0.081 - 0.155]	0.111 [0.101 - 0.123]	0.252 [0.226 - 0.280]
-3.5	0.028 [0.014 - 0.055]	0.065 [0.047 - 0.091]	0.168 [0.110 - 0.249]	0.107 [0.082 - 0.140]	0.114 [0.104 - 0.125]	0.254 [0.231 - 0.280]
-3	0.031 [0.017 - 0.056]	0.069 [0.051 - 0.092]	0.169 [0.116 - 0.239]	0.102 [0.082 - 0.127]	0.116 [0.107 - 0.126]	0.256 [0.235 - 0.279]
-2.5	0.033 [0.019 - 0.058]	0.072 [0.055 - 0.094]	0.170 [0.122 - 0.233]	0.098 [0.081 - 0.117]	0.119 [0.111 - 0.128]	0.258 [0.238 - 0.279]
-2	0.036 [0.022 - 0.059]	0.075 [0.059 - 0.096]	0.172 [0.127 - 0.230]	0.094 [0.080 - 0.110]	0.122 [0.114 - 0.130]	0.260 [0.242 - 0.279]
-1.5	0.039 [0.025 - 0.061]	0.079 [0.063 - 0.098]	0.175 [0.131 - 0.230]	0.091 [0.079 - 0.105]	0.124 [0.117 - 0.132]	0.262 [0.246 - 0.279]
-1	0.042 [0.028 - 0.063]	0.083 [0.068 - 0.101]	0.179 [0.135 - 0.234]	0.090 [0.078 - 0.102]	0.127 [0.120 - 0.134]	0.264 [0.248 - 0.280]
-0.5	0.046 [0.032 - 0.066]	0.087 [0.072 - 0.105]	0.185 [0.140 - 0.240]	0.089 [0.078 - 0.101]	0.130 [0.123 - 0.136]	0.266 [0.251 - 0.282]
Mean	0.050 [0.035 - 0.069]	0.091 [0.076 - 0.109]	0.192 [0.145 - 0.250]	0.090 [0.079 - 0.102]	0.132 [0.126 - 0.138]	0.268 [0.253 - 0.284]
0.5	0.054 [0.039 - 0.074]	0.096 [0.080 - 0.114]	0.201 [0.150 - 0.263]	0.091 [0.080 - 0.104]	0.135 [0.129 - 0.141]	0.270 [0.254 - 0.287]
1	0.058 [0.042 - 0.080]	0.100 [0.083 - 0.120]	0.211 [0.156 - 0.278]	0.094 [0.082 - 0.107]	0.138 [0.132 - 0.145]	0.272 [0.254 - 0.290]
1.5	0.063 [0.045 - 0.087]	0.105 [0.086 - 0.127]	0.222 [0.162 - 0.296]	0.096 [0.084 - 0.111]	0.141 [0.134 - 0.148]	0.274 [0.255 - 0.294]
2	0.068 [0.047 - 0.096]	0.110 [0.089 - 0.135]	0.233 [0.167 - 0.316]	0.099 [0.085 - 0.115]	0.144 [0.136 - 0.152]	0.276 [0.255 - 0.299]
2.5	0.073 [0.049 - 0.107]	0.115 [0.091 - 0.144]	0.246 [0.171 - 0.340]	0.102 [0.087 - 0.120]	0.147 [0.138 - 0.156]	0.278 [0.254 - 0.303]
3	0.079 [0.051 - 0.120]	0.120 [0.093 - 0.154]	0.259 [0.175 - 0.367]	0.105 [0.088 - 0.125]	0.150 [0.140 - 0.161]	0.280 [0.254 - 0.308]
3.5	0.085 [0.052 - 0.135]	0.126 [0.095 - 0.164]	0.274 [0.177 - 0.398]	0.109 [0.090 - 0.131]	0.153 [0.142 - 0.165]	0.282 [0.253 - 0.313]
4	0.092 [0.054 - 0.152]	0.132 [0.097 - 0.176]	0.291 [0.180 - 0.435]	0.112 [0.090 - 0.138]	0.156 [0.143 - 0.170]	0.284 [0.252 - 0.318]
4.5	0.099 [0.055 - 0.171]	0.138 [0.099 - 0.189]	0.309 [0.181 - 0.475]	0.115 [0.091 - 0.146]	0.159 [0.145 - 0.175]	0.286 [0.252 - 0.324]
5	0.106 [0.056 - 0.192]	0.144 [0.100 - 0.202]	0.330 [0.182 - 0.520]	0.118 [0.090 - 0.154]	0.163 [0.147 - 0.180]	0.288 [0.251 - 0.329]
Age		30-34 yrs			>34 yrs	
Weight gain	Normal weight (n=12,302)	Underweight (n=2,460)	Overweight/Obese (n=4,749)	Normal weight (n=5,750)	Underweight (n=704)	Overweight/Obese (n=3,221)
-5	0.133 [0.102 - 0.171]	0.179 [0.160 - 0.200]	0.327 [0.298 - 0.357]	0.277 [0.159 - 0.437]	0.265 [0.219 - 0.316]	0.448 [0.411 - 0.487]
-4.5	0.134 [0.105 - 0.169]	0.181 [0.164 - 0.199]	0.325 [0.299 - 0.352]	0.268 [0.166 - 0.403]	0.264 [0.225 - 0.307]	0.444 [0.410 - 0.479]
-4	0.135 [0.109 - 0.168]	0.182 [0.167 - 0.199]	0.323 [0.299 - 0.347]	0.259 [0.172 - 0.371]	0.263 [0.231 - 0.298]	0.440 [0.409 - 0.471]
-3.5	0.137 [0.112 - 0.166]	0.184 [0.170 - 0.198]	0.320 [0.299 - 0.342]	0.250 [0.176 - 0.342]	0.262 [0.235 - 0.291]	0.435 [0.408 - 0.463]
-3	0.138 [0.115 - 0.164]	0.186 [0.173 - 0.198]	0.318 [0.299 - 0.338]	0.241 [0.178 - 0.317]	0.262 [0.239 - 0.285]	0.431 [0.406 - 0.456]
-2.5	0.139 [0.118 - 0.163]	0.187 [0.176 - 0.198]	0.316 [0.299 - 0.334]	0.232 [0.180 - 0.295]	0.261 [0.242 - 0.281]	0.427 [0.404 - 0.449]
-2	0.140 [0.122 - 0.162]	0.189 [0.179 - 0.199]	0.314 [0.298 - 0.330]	0.224 [0.179 - 0.277]	0.260 [0.244 - 0.278]	0.422 [0.402 - 0.443]
-1.5	0.142 [0.125 - 0.161]	0.191 [0.182 - 0.200]	0.312 [0.298 - 0.326]	0.217 [0.177 - 0.263]	0.260 [0.245 - 0.275]	0.418 [0.399 - 0.437]
-1	0.143 [0.128 - 0.160]	0.192 [0.185 - 0.201]	0.310 [0.296 - 0.323]	0.211 [0.175 - 0.252]	0.260 [0.245 - 0.274]	0.414 [0.396 - 0.432]
-0.5	0.144 [0.130 - 0.160]	0.194 [0.187 - 0.202]	0.307 [0.294 - 0.321]	0.206 [0.172 - 0.244]	0.259 [0.246 - 0.274]	0.410 [0.392 - 0.427]
Mean	0.146 [0.132 - 0.161]	0.197 [0.189 - 0.204]	0.305 [0.292 - 0.319]	0.201 [0.169 - 0.238]	0.260 [0.246 - 0.274]	0.405 [0.387 - 0.424]
0.5	0.147 [0.133 - 0.162]	0.199 [0.191 - 0.207]	0.303 [0.288 - 0.318]	0.197 [0.165 - 0.234]	0.260 [0.246 - 0.275]	0.401 [0.382 - 0.421]
1	0.148 [0.134 - 0.164]	0.201 [0.193 - 0.209]	0.301 [0.285 - 0.318]	0.194 [0.161 - 0.232]	0.261 [0.246 - 0.277]	0.397 [0.376 - 0.419]
1.5	0.150 [0.134 - 0.167]	0.203 [0.195 - 0.213]	0.299 [0.281 - 0.317]	0.192 [0.158 - 0.231]	0.263 [0.247 - 0.280]	0.393 [0.370 - 0.417]
2	0.151 [0.134 - 0.170]	0.206 [0.196 - 0.216]	0.297 [0.277 - 0.317]	0.190 [0.155 - 0.232]	0.266 [0.249 - 0.284]	0.389 [0.364 - 0.415]
2.5	0.152 [0.133 - 0.174]	0.208 [0.198 - 0.220]	0.295 [0.273 - 0.317]	0.190 [0.152 - 0.235]	0.270 [0.251 - 0.289]	0.385 [0.357 - 0.414]
3	0.154 [0.132 - 0.178]	0.211 [0.199 - 0.224]	0.293 [0.269 - 0.318]	0.190 [0.150 - 0.239]	0.274 [0.254 - 0.296]	0.381 [0.350 - 0.413]
3.5	0.155 [0.131 - 0.183]	0.214 [0.200 - 0.228]	0.291 [0.265 - 0.318]	0.192 [0.148 - 0.246]	0.280 [0.258 - 0.304]	0.377 [0.344 - 0.412]
4	0.157 [0.130 - 0.187]	0.217 [0.201 - 0.233]	0.289 [0.260 - 0.319]	0.195 [0.146 - 0.254]	0.287 [0.262 - 0.314]	0.374 [0.337 - 0.412]
4.5	0.158 [0.129 - 0.192]	0.219 [0.202 - 0.238]	0.287 [0.256 - 0.319]	0.198 [0.144 - 0.265]	0.295 [0.266 - 0.325]	0.370 [0.330 - 0.411]
5	0.159 [0.127 - 0.198]	0.222 [0.203 - 0.243]	0.285 [0.252 - 0.320]	0.202 [0.143 - 0.278]	0.302 [0.270 - 0.337]	0.366 [0.323 - 0.411]

1.2

Preeclampsia

Age		<25 yrs			25-29 yrs	
Weight gain	Normal weight (n=1,324)	Underweight (n=698)	Overweight/Obese (n=314)	Normal weight (n=12,406)	Underweight (n=3,943)	Overweight/Obese (n=3,249)
-5	0.003 [0.000 - 0.037]	0.018 [0.007 - 0.044]	0.029 [0.008 - 0.098]	0.005 [0.002 - 0.013]	0.009 [0.006 - 0.014]	0.034 [0.017 - 0.068]
-4.5	0.003 [0.000 - 0.032]	0.018 [0.008 - 0.041]	0.030 [0.009 - 0.091]	0.005 [0.002 - 0.013]	0.009 [0.006 - 0.014]	0.032 [0.018 - 0.056]
-4	0.004 [0.000 - 0.029]	0.018 [0.008 - 0.039]	0.031 [0.011 - 0.085]	0.005 [0.002 - 0.012]	0.009 [0.006 - 0.013]	0.029 [0.018 - 0.047]
-3.5	0.004 [0.001 - 0.026]	0.018 [0.009 - 0.036]	0.032 [0.012 - 0.079]	0.005 [0.002 - 0.012]	0.009 [0.006 - 0.013]	0.027 [0.018 - 0.041]
-3	0.004 [0.001 - 0.023]	0.018 [0.010 - 0.034]	0.032 [0.014 - 0.075]	0.005 [0.003 - 0.011]	0.009 [0.007 - 0.013]	0.026 [0.018 - 0.036]
-2.5	0.004 [0.001 - 0.021]	0.019 [0.011 - 0.033]	0.033 [0.015 - 0.071]	0.005 [0.003 - 0.011]	0.009 [0.007 - 0.012]	0.024 [0.017 - 0.033]
-2	0.005 [0.001 - 0.019]	0.019 [0.011 - 0.031]	0.034 [0.017 - 0.068]	0.006 [0.003 - 0.010]	0.009 [0.007 - 0.012]	0.022 [0.017 - 0.030]
-1.5	0.005 [0.001 - 0.018]	0.019 [0.012 - 0.030]	0.035 [0.018 - 0.066]	0.006 [0.003 - 0.010]	0.009 [0.007 - 0.012]	0.021 [0.016 - 0.028]
-1	0.005 [0.002 - 0.017]	0.019 [0.012 - 0.029]	0.036 [0.020 - 0.065]	0.006 [0.004 - 0.010]	0.009 [0.008 - 0.011]	0.020 [0.015 - 0.027]
-0.5	0.005 [0.002 - 0.016]	0.019 [0.013 - 0.029]	0.037 [0.020 - 0.065]	0.006 [0.004 - 0.010]	0.009 [0.008 - 0.011]	0.020 [0.015 - 0.027]
Mean	0.006 [0.002 - 0.016]	0.019 [0.013 - 0.029]	0.038 [0.021 - 0.067]	0.006 [0.004 - 0.010]	0.009 [0.008 - 0.011]	0.020 [0.014 - 0.027]
0.5	0.006 [0.002 - 0.016]	0.020 [0.013 - 0.029]	0.039 [0.021 - 0.071]	0.007 [0.004 - 0.010]	0.009 [0.008 - 0.011]	0.020 [0.014 - 0.027]
1	0.007 [0.002 - 0.017]	0.020 [0.013 - 0.030]	0.040 [0.020 - 0.076]	0.007 [0.005 - 0.010]	0.009 [0.008 - 0.011]	0.020 [0.014 - 0.028]
1.5	0.007 [0.002 - 0.020]	0.020 [0.012 - 0.032]	0.041 [0.019 - 0.083]	0.007 [0.005 - 0.011]	0.009 [0.008 - 0.012]	0.021 [0.015 - 0.030]
2	0.007 [0.002 - 0.023]	0.020 [0.012 - 0.034]	0.042 [0.018 - 0.091]	0.007 [0.005 - 0.011]	0.009 [0.007 - 0.012]	0.022 [0.015 - 0.032]
2.5	0.008 [0.002 - 0.027]	0.020 [0.011 - 0.036]	0.043 [0.017 - 0.101]	0.007 [0.004 - 0.012]	0.009 [0.007 - 0.012]	0.024 [0.016 - 0.035]
3	0.008 [0.002 - 0.032]	0.020 [0.011 - 0.038]	0.044 [0.016 - 0.113]	0.008 [0.004 - 0.013]	0.009 [0.007 - 0.013]	0.026 [0.017 - 0.039]
3.5	0.009 [0.002 - 0.040]	0.020 [0.010 - 0.041]	0.045 [0.015 - 0.126]	0.008 [0.004 - 0.015]	0.009 [0.007 - 0.013]	0.029 [0.018 - 0.044]
4	0.009 [0.002 - 0.049]	0.021 [0.009 - 0.045]	0.046 [0.014 - 0.141]	0.008 [0.004 - 0.016]	0.010 [0.007 - 0.014]	0.032 [0.020 - 0.051]
4.5	0.010 [0.002 - 0.061]	0.021 [0.009 - 0.048]	0.047 [0.013 - 0.158]	0.008 [0.004 - 0.018]	0.010 [0.006 - 0.014]	0.036 [0.022 - 0.058]
5	0.011 [0.001 - 0.077]	0.021 [0.008 - 0.052]	0.048 [0.012 - 0.177]	0.009 [0.004 - 0.020]	0.010 [0.006 - 0.015]	0.040 [0.024 - 0.068]
Age		30-34 yrs			>34 yrs	
Weight gain	Normal weight (n=12,302)	Underweight (n=2,460)	Overweight/Obese (n=4,749)	Normal weight (n=5,750)	Underweight (n=704)	Overweight/Obese (n=3,221)
-5	0.002 [0.000 - 0.005]	0.011 [0.006 - 0.020]	0.040 [0.025 - 0.062]	0.010 [0.000 - 0.299]	0.016 [0.010 - 0.026]	0.053 [0.036 - 0.076]
-4.5	0.002 [0.001 - 0.005]	0.010 [0.006 - 0.018]	0.038 [0.026 - 0.055]	0.011 [0.001 - 0.192]	0.016 [0.010 - 0.025]	0.051 [0.037 - 0.071]
-4	0.002 [0.001 - 0.006]	0.010 [0.006 - 0.016]	0.036 [0.026 - 0.049]	0.013 [0.001 - 0.126]	0.016 [0.010 - 0.024]	0.050 [0.037 - 0.067]
-3.5	0.002 [0.001 - 0.006]	0.010 [0.007 - 0.015]	0.034 [0.026 - 0.045]	0.015 [0.002 - 0.088]	0.016 [0.011 - 0.023]	0.049 [0.038 - 0.063]
-3	0.003 [0.001 - 0.006]	0.010 [0.007 - 0.014]	0.032 [0.026 - 0.041]	0.016 [0.004 - 0.068]	0.016 [0.011 - 0.022]	0.047 [0.038 - 0.059]
-2.5	0.003 [0.001 - 0.007]	0.010 [0.007 - 0.013]	0.031 [0.025 - 0.038]	0.018 [0.006 - 0.057]	0.016 [0.012 - 0.022]	0.046 [0.038 - 0.057]
-2	0.003 [0.001 - 0.007]	0.010 [0.008 - 0.013]	0.030 [0.024 - 0.036]	0.020 [0.008 - 0.051]	0.016 [0.012 - 0.021]	0.045 [0.038 - 0.055]
-1.5	0.004 [0.002 - 0.008]	0.010 [0.008 - 0.012]	0.029 [0.024 - 0.035]	0.022 [0.010 - 0.050]	0.016 [0.012 - 0.020]	0.045 [0.037 - 0.053]
-1	0.004 [0.002 - 0.008]	0.010 [0.008 - 0.012]	0.028 [0.023 - 0.034]	0.024 [0.011 - 0.049]	0.016 [0.013 - 0.020]	0.044 [0.037 - 0.052]
-0.5	0.005 [0.003 - 0.009]	0.010 [0.008 - 0.012]	0.028 [0.023 - 0.034]	0.025 [0.012 - 0.049]	0.016 [0.013 - 0.020]	0.043 [0.036 - 0.052]
Mean	0.005 [0.003 - 0.010]	0.011 [0.009 - 0.013]	0.028 [0.023 - 0.034]	0.025 [0.012 - 0.050]	0.016 [0.013 - 0.019]	0.043 [0.035 - 0.051]
0.5	0.006 [0.004 - 0.010]	0.011 [0.009 - 0.013]	0.028 [0.022 - 0.035]	0.024 [0.012 - 0.049]	0.016 [0.013 - 0.019]	0.042 [0.034 - 0.051]
1	0.007 [0.004 - 0.012]	0.011 [0.009 - 0.014]	0.028 [0.022 - 0.035]	0.022 [0.010 - 0.048]	0.016 [0.013 - 0.020]	0.042 [0.034 - 0.052]
1.5	0.008 [0.005 - 0.013]	0.012 [0.010 - 0.015]	0.029 [0.022 - 0.037]	0.019 [0.008 - 0.045]	0.016 [0.012 - 0.020]	0.041 [0.033 - 0.052]
2	0.009 [0.006 - 0.015]	0.012 [0.010 - 0.016]	0.029 [0.022 - 0.038]	0.015 [0.005 - 0.041]	0.016 [0.012 - 0.021]	0.041 [0.032 - 0.053]
2.5	0.010 [0.006 - 0.017]	0.013 [0.010 - 0.017]	0.029 [0.022 - 0.040]	0.011 [0.003 - 0.038]	0.016 [0.012 - 0.021]	0.041 [0.031 - 0.054]
3	0.012 [0.007 - 0.020]	0.014 [0.011 - 0.018]	0.030 [0.021 - 0.042]	0.007 [0.001 - 0.037]	0.016 [0.011 - 0.022]	0.041 [0.029 - 0.056]
3.5	0.013 [0.008 - 0.023]	0.015 [0.011 - 0.020]	0.030 [0.021 - 0.044]	0.005 [0.001 - 0.038]	0.016 [0.011 - 0.023]	0.040 [0.028 - 0.057]
4	0.015 [0.008 - 0.027]	0.016 [0.011 - 0.021]	0.031 [0.020 - 0.046]	0.003 [0.000 - 0.043]	0.016 [0.011 - 0.023]	0.040 [0.027 - 0.059]
4.5	0.017 [0.009 - 0.033]	0.016 [0.011 - 0.023]	0.031 [0.019 - 0.049]	0.002 [0.000 - 0.051]	0.016 [0.010 - 0.024]	0.040 [0.026 - 0.062]
5	0.020 [0.010 - 0.039]	0.017 [0.012 - 0.026]	0.031 [0.019 - 0.053]	0.002 [0.000 - 0.067]	0.016 [0.010 - 0.025]	0.040 [0.025 - 0.064]

1.3

Cesarean delivery

Age		<25 yrs			25-29 yrs	
Weight gain	Normal weight (n=1,324)	Underweight (n=698)	Overweight/Obese (n=314)	Normal weight (n=12,406)	Underweight (n=3,943)	Overweight/Obese (n=3,249)
-5	0.299 [0.191 - 0.436]	0.506 [0.406 - 0.606]	0.435 [0.291 - 0.591]	0.384 [0.345 - 0.425]	0.431 [0.405 - 0.458]	0.526 [0.488 - 0.564]
-4.5	0.305 [0.210 - 0.420]	0.494 [0.411 - 0.577]	0.449 [0.324 - 0.580]	0.386 [0.350 - 0.424]	0.435 [0.412 - 0.459]	0.533 [0.498 - 0.568]
-4	0.311 [0.228 - 0.408]	0.481 [0.412 - 0.550]	0.462 [0.355 - 0.574]	0.388 [0.355 - 0.422]	0.439 [0.418 - 0.460]	0.539 [0.508 - 0.571]
-3.5	0.317 [0.246 - 0.398]	0.468 [0.411 - 0.526]	0.475 [0.382 - 0.571]	0.390 [0.359 - 0.421]	0.443 [0.425 - 0.461]	0.546 [0.517 - 0.575]
-3	0.324 [0.263 - 0.392]	0.455 [0.407 - 0.505]	0.488 [0.405 - 0.571]	0.392 [0.364 - 0.420]	0.447 [0.431 - 0.463]	0.553 [0.527 - 0.578]
-2.5	0.331 [0.277 - 0.389]	0.443 [0.401 - 0.486]	0.499 [0.424 - 0.574]	0.393 [0.369 - 0.419]	0.451 [0.437 - 0.465]	0.559 [0.536 - 0.582]
-2	0.338 [0.290 - 0.390]	0.433 [0.395 - 0.472]	0.508 [0.438 - 0.579]	0.395 [0.373 - 0.418]	0.455 [0.442 - 0.467]	0.566 [0.545 - 0.587]
-1.5	0.346 [0.302 - 0.393]	0.425 [0.390 - 0.461]	0.515 [0.447 - 0.583]	0.397 [0.377 - 0.417]	0.459 [0.448 - 0.470]	0.572 [0.553 - 0.591]
-1	0.354 [0.312 - 0.400]	0.420 [0.386 - 0.454]	0.520 [0.452 - 0.587]	0.399 [0.381 - 0.417]	0.463 [0.453 - 0.473]	0.579 [0.561 - 0.597]
-0.5	0.363 [0.320 - 0.408]	0.417 [0.383 - 0.451]	0.521 [0.452 - 0.589]	0.401 [0.384 - 0.417]	0.468 [0.458 - 0.477]	0.585 [0.568 - 0.603]
Mean	0.370 [0.327 - 0.416]	0.416 [0.382 - 0.451]	0.519 [0.449 - 0.589]	0.403 [0.387 - 0.418]	0.472 [0.463 - 0.482]	0.592 [0.574 - 0.609]
0.5	0.377 [0.332 - 0.424]	0.416 [0.381 - 0.453]	0.515 [0.442 - 0.588]	0.404 [0.389 - 0.420]	0.477 [0.467 - 0.487]	0.598 [0.580 - 0.617]
1	0.381 [0.334 - 0.431]	0.418 [0.381 - 0.456]	0.511 [0.433 - 0.588]	0.406 [0.390 - 0.423]	0.482 [0.472 - 0.492]	0.605 [0.585 - 0.625]
1.5	0.384 [0.334 - 0.437]	0.421 [0.381 - 0.462]	0.506 [0.423 - 0.589]	0.408 [0.391 - 0.426]	0.487 [0.476 - 0.498]	0.611 [0.589 - 0.633]
2	0.385 [0.331 - 0.442]	0.425 [0.382 - 0.469]	0.501 [0.411 - 0.592]	0.410 [0.390 - 0.430]	0.492 [0.480 - 0.504]	0.617 [0.593 - 0.641]
2.5	0.384 [0.325 - 0.446]	0.431 [0.384 - 0.479]	0.497 [0.398 - 0.597]	0.412 [0.390 - 0.434]	0.497 [0.483 - 0.510]	0.624 [0.597 - 0.650]
3	0.381 [0.317 - 0.450]	0.437 [0.385 - 0.490]	0.492 [0.382 - 0.604]	0.414 [0.389 - 0.439]	0.502 [0.487 - 0.517]	0.630 [0.600 - 0.659]
3.5	0.376 [0.305 - 0.453]	0.444 [0.387 - 0.504]	0.487 [0.364 - 0.613]	0.416 [0.388 - 0.443]	0.507 [0.490 - 0.524]	0.636 [0.604 - 0.667]
4	0.370 [0.290 - 0.458]	0.452 [0.387 - 0.518]	0.481 [0.342 - 0.623]	0.417 [0.387 - 0.448]	0.512 [0.493 - 0.531]	0.642 [0.607 - 0.676]
4.5	0.363 [0.273 - 0.464]	0.459 [0.386 - 0.533]	0.474 [0.318 - 0.635]	0.419 [0.386 - 0.454]	0.518 [0.496 - 0.539]	0.648 [0.610 - 0.685]
5	0.355 [0.254 - 0.471]	0.465 [0.383 - 0.550]	0.465 [0.291 - 0.647]	0.421 [0.384 - 0.459]	0.523 [0.499 - 0.546]	0.654 [0.613 - 0.694]
Age		30-34 yrs			>34 yrs	
Weight gain	Normal weight (n=12,302)	Underweight (n=2,460)	Overweight/Obese (n=4,749)	Normal weight (n=5,750)	Underweight (n=704)	Overweight/Obese (n=3,221)
-5	0.488 [0.436 - 0.541]	0.558 [0.535 - 0.581]	0.654 [0.624 - 0.683]	0.652 [0.551 - 0.742]	0.711 [0.636 - 0.775]	0.783 [0.732 - 0.826]
-4.5	0.492 [0.444 - 0.540]	0.563 [0.542 - 0.584]	0.656 [0.629 - 0.683]	0.660 [0.567 - 0.742]	0.710 [0.652 - 0.762]	0.785 [0.744 - 0.820]
-4	0.495 [0.451 - 0.539]	0.568 [0.549 - 0.587]	0.658 [0.633 - 0.682]	0.667 [0.583 - 0.742]	0.709 [0.665 - 0.749]	0.787 [0.754 - 0.816]
-3.5	0.499 [0.459 - 0.539]	0.573 [0.555 - 0.590]	0.660 [0.638 - 0.682]	0.675 [0.599 - 0.742]	0.709 [0.676 - 0.741]	0.789 [0.762 - 0.814]
-3	0.502 [0.466 - 0.538]	0.577 [0.562 - 0.592]	0.662 [0.642 - 0.682]	0.682 [0.614 - 0.743]	0.711 [0.684 - 0.737]	0.791 [0.768 - 0.813]
-2.5	0.505 [0.473 - 0.538]	0.582 [0.568 - 0.595]	0.664 [0.646 - 0.682]	0.689 [0.628 - 0.744]	0.715 [0.692 - 0.737]	0.794 [0.773 - 0.813]
-2	0.509 [0.480 - 0.538]	0.587 [0.574 - 0.599]	0.666 [0.650 - 0.682]	0.696 [0.642 - 0.745]	0.722 [0.702 - 0.741]	0.797 [0.778 - 0.816]
-1.5	0.512 [0.486 - 0.538]	0.591 [0.580 - 0.602]	0.668 [0.654 - 0.683]	0.703 [0.655 - 0.747]	0.731 [0.713 - 0.749]	0.802 [0.783 - 0.819]
-1	0.516 [0.492 - 0.539]	0.596 [0.586 - 0.606]	0.670 [0.656 - 0.684]	0.710 [0.667 - 0.749]	0.741 [0.724 - 0.758]	0.807 [0.789 - 0.824]
-0.5	0.519 [0.498 - 0.540]	0.601 [0.591 - 0.610]	0.672 [0.658 - 0.686]	0.717 [0.678 - 0.752]	0.752 [0.735 - 0.768]	0.812 [0.794 - 0.829]
Mean	0.522 [0.502 - 0.543]	0.605 [0.596 - 0.614]	0.674 [0.660 - 0.688]	0.723 [0.688 - 0.757]	0.761 [0.745 - 0.777]	0.818 [0.800 - 0.835]
0.5	0.526 [0.506 - 0.546]	0.610 [0.601 - 0.619]	0.676 [0.661 - 0.691]	0.730 [0.695 - 0.762]	0.768 [0.751 - 0.784]	0.823 [0.804 - 0.841]
1	0.529 [0.508 - 0.550]	0.614 [0.605 - 0.624]	0.678 [0.661 - 0.695]	0.736 [0.701 - 0.769]	0.773 [0.756 - 0.790]	0.828 [0.808 - 0.847]
1.5	0.533 [0.510 - 0.555]	0.619 [0.608 - 0.629]	0.680 [0.661 - 0.698]	0.743 [0.705 - 0.777]	0.776 [0.757 - 0.793]	0.832 [0.810 - 0.851]
2	0.536 [0.511 - 0.561]	0.624 [0.612 - 0.635]	0.682 [0.661 - 0.702]	0.749 [0.709 - 0.786]	0.777 [0.757 - 0.796]	0.835 [0.811 - 0.856]
2.5	0.539 [0.511 - 0.567]	0.628 [0.615 - 0.641]	0.684 [0.661 - 0.706]	0.755 [0.711 - 0.795]	0.778 [0.757 - 0.799]	0.836 [0.811 - 0.859]
3	0.543 [0.511 - 0.574]	0.633 [0.618 - 0.647]	0.686 [0.661 - 0.710]	0.761 [0.712 - 0.804]	0.780 [0.756 - 0.802]	0.837 [0.809 - 0.862]
3.5	0.546 [0.511 - 0.581]	0.637 [0.621 - 0.653]	0.688 [0.660 - 0.715]	0.767 [0.714 - 0.814]	0.783 [0.756 - 0.807]	0.838 [0.805 - 0.865]
4	0.550 [0.510 - 0.588]	0.642 [0.624 - 0.659]	0.690 [0.659 - 0.719]	0.773 [0.714 - 0.823]	0.787 [0.757 - 0.813]	0.837 [0.801 - 0.868]
4.5	0.553 [0.510 - 0.595]	0.646 [0.627 - 0.665]	0.692 [0.659 - 0.723]	0.779 [0.715 - 0.832]	0.792 [0.760 - 0.821]	0.836 [0.795 - 0.871]
5	0.556 [0.509 - 0.603]	0.650 [0.629 - 0.671]	0.694 [0.658 - 0.727]	0.785 [0.715 - 0.841]	0.800 [0.764 - 0.831]	0.835 [0.787 - 0.873]

1.4

Preterm delivery

Age		<25 yrs			25-29 yrs	
Weight gain	Normal weight (n=1,324)	Underweight (n=698)	Overweight/Obese (n=314)	Normal weight (n=12,406)	Underweight (n=3,943)	Overweight/Obese (n=3,249)
-5	0.104 [0.055 - 0.187]	0.067 [0.041 - 0.107]	0.099 [0.045 - 0.205]	0.045 [0.029 - 0.067]	0.054 [0.040 - 0.074]	0.049 [0.036 - 0.067]
-4.5	0.100 [0.056 - 0.172]	0.066 [0.042 - 0.102]	0.098 [0.048 - 0.188]	0.044 [0.030 - 0.064]	0.052 [0.040 - 0.067]	0.050 [0.038 - 0.066]
-4	0.096 [0.056 - 0.157]	0.066 [0.044 - 0.098]	0.097 [0.052 - 0.175]	0.043 [0.030 - 0.061]	0.050 [0.040 - 0.062]	0.051 [0.039 - 0.066]
-3.5	0.092 [0.057 - 0.144]	0.066 [0.046 - 0.094]	0.096 [0.055 - 0.163]	0.042 [0.031 - 0.058]	0.048 [0.040 - 0.057]	0.052 [0.041 - 0.065]
-3	0.088 [0.058 - 0.133]	0.066 [0.047 - 0.091]	0.095 [0.058 - 0.153]	0.041 [0.031 - 0.055]	0.046 [0.039 - 0.054]	0.053 [0.042 - 0.065]
-2.5	0.085 [0.058 - 0.122]	0.065 [0.049 - 0.088]	0.095 [0.060 - 0.145]	0.041 [0.031 - 0.052]	0.044 [0.038 - 0.050]	0.054 [0.044 - 0.065]
-2	0.081 [0.058 - 0.113]	0.065 [0.050 - 0.085]	0.094 [0.062 - 0.140]	0.040 [0.032 - 0.050]	0.042 [0.038 - 0.048]	0.055 [0.046 - 0.065]
-1.5	0.078 [0.057 - 0.105]	0.065 [0.051 - 0.083]	0.094 [0.064 - 0.136]	0.039 [0.032 - 0.048]	0.041 [0.037 - 0.046]	0.055 [0.047 - 0.065]
-1	0.075 [0.056 - 0.099]	0.065 [0.052 - 0.081]	0.094 [0.065 - 0.135]	0.039 [0.032 - 0.046]	0.040 [0.036 - 0.045]	0.056 [0.049 - 0.065]
-0.5	0.072 [0.054 - 0.094]	0.065 [0.052 - 0.080]	0.094 [0.065 - 0.135]	0.038 [0.032 - 0.045]	0.039 [0.035 - 0.043]	0.057 [0.050 - 0.066]
Mean	0.069 [0.052 - 0.091]	0.064 [0.052 - 0.080]	0.095 [0.065 - 0.137]	0.037 [0.032 - 0.044]	0.038 [0.035 - 0.043]	0.058 [0.051 - 0.067]
0.5	0.066 [0.049 - 0.089]	0.064 [0.051 - 0.080]	0.096 [0.064 - 0.141]	0.036 [0.031 - 0.043]	0.038 [0.034 - 0.042]	0.059 [0.051 - 0.069]
1	0.063 [0.045 - 0.088]	0.064 [0.050 - 0.081]	0.097 [0.063 - 0.147]	0.036 [0.030 - 0.043]	0.037 [0.033 - 0.042]	0.060 [0.052 - 0.071]
1.5	0.061 [0.042 - 0.088]	0.064 [0.049 - 0.083]	0.098 [0.061 - 0.154]	0.035 [0.029 - 0.043]	0.037 [0.033 - 0.042]	0.061 [0.052 - 0.073]
2	0.058 [0.038 - 0.088]	0.064 [0.047 - 0.085]	0.100 [0.059 - 0.163]	0.034 [0.028 - 0.043]	0.037 [0.032 - 0.042]	0.063 [0.052 - 0.075]
2.5	0.056 [0.035 - 0.089]	0.063 [0.046 - 0.087]	0.101 [0.057 - 0.174]	0.034 [0.027 - 0.043]	0.036 [0.032 - 0.042]	0.064 [0.052 - 0.078]
3	0.054 [0.031 - 0.090]	0.063 [0.044 - 0.090]	0.103 [0.054 - 0.186]	0.033 [0.025 - 0.044]	0.036 [0.031 - 0.043]	0.065 [0.051 - 0.081]
3.5	0.051 [0.028 - 0.091]	0.063 [0.042 - 0.093]	0.104 [0.051 - 0.200]	0.033 [0.024 - 0.044]	0.036 [0.030 - 0.044]	0.066 [0.051 - 0.084]
4	0.049 [0.026 - 0.092]	0.063 [0.040 - 0.096]	0.106 [0.048 - 0.216]	0.032 [0.023 - 0.045]	0.037 [0.030 - 0.045]	0.067 [0.051 - 0.088]
4.5	0.047 [0.023 - 0.093]	0.062 [0.039 - 0.099]	0.107 [0.045 - 0.233]	0.031 [0.022 - 0.045]	0.037 [0.029 - 0.046]	0.068 [0.050 - 0.091]
5	0.045 [0.021 - 0.095]	0.062 [0.037 - 0.103]	0.108 [0.042 - 0.252]	0.031 [0.021 - 0.046]	0.037 [0.029 - 0.048]	0.069 [0.050 - 0.095]
Age		30-34 yrs			>34 yrs	
Weight gain	Normal weight (n=12,302)	Underweight (n=2,460)	Overweight/Obese (n=4,749)	Normal weight (n=5,750)	Underweight (n=704)	Overweight/Obese (n=3,221)
-5	0.111 [0.054 - 0.214]	0.058 [0.043 - 0.079]	0.060 [0.042 - 0.085]	0.109 [0.056 - 0.203]	0.090 [0.059 - 0.135]	0.073 [0.056 - 0.094]
-4.5	0.100 [0.055 - 0.174]	0.056 [0.044 - 0.073]	0.059 [0.044 - 0.079]	0.106 [0.058 - 0.188]	0.086 [0.061 - 0.120]	0.073 [0.058 - 0.092]
-4	0.089 [0.055 - 0.142]	0.054 [0.044 - 0.067]	0.059 [0.046 - 0.075]	0.104 [0.060 - 0.174]	0.081 [0.061 - 0.107]	0.074 [0.059 - 0.091]
-3.5	0.081 [0.054 - 0.119]	0.053 [0.044 - 0.063]	0.058 [0.047 - 0.071]	0.101 [0.061 - 0.162]	0.077 [0.061 - 0.097]	0.074 [0.061 - 0.090]
-3	0.073 [0.052 - 0.101]	0.051 [0.044 - 0.059]	0.058 [0.048 - 0.069]	0.098 [0.063 - 0.151]	0.073 [0.060 - 0.088]	0.075 [0.063 - 0.089]
-2.5	0.067 [0.050 - 0.089]	0.049 [0.043 - 0.056]	0.057 [0.049 - 0.067]	0.096 [0.064 - 0.141]	0.069 [0.059 - 0.082]	0.075 [0.064 - 0.088]
-2	0.062 [0.048 - 0.080]	0.048 [0.043 - 0.054]	0.057 [0.049 - 0.066]	0.093 [0.065 - 0.132]	0.066 [0.057 - 0.076]	0.076 [0.065 - 0.087]
-1.5	0.058 [0.046 - 0.073]	0.047 [0.042 - 0.052]	0.057 [0.049 - 0.066]	0.091 [0.066 - 0.124]	0.063 [0.055 - 0.072]	0.076 [0.067 - 0.087]
-1	0.055 [0.044 - 0.068]	0.046 [0.042 - 0.051]	0.057 [0.049 - 0.065]	0.089 [0.066 - 0.118]	0.061 [0.053 - 0.069]	0.077 [0.068 - 0.087]
-0.5	0.052 [0.042 - 0.064]	0.045 [0.041 - 0.050]	0.056 [0.049 - 0.065]	0.086 [0.066 - 0.113]	0.059 [0.052 - 0.067]	0.077 [0.068 - 0.087]
Mean	0.049 [0.039 - 0.061]	0.045 [0.041 - 0.049]	0.056 [0.048 - 0.065]	0.084 [0.065 - 0.109]	0.058 [0.050 - 0.066]	0.078 [0.068 - 0.088]
0.5	0.046 [0.037 - 0.058]	0.044 [0.040 - 0.049]	0.055 [0.047 - 0.065]	0.082 [0.063 - 0.106]	0.057 [0.049 - 0.065]	0.078 [0.068 - 0.089]
1	0.044 [0.034 - 0.056]	0.044 [0.040 - 0.049]	0.055 [0.046 - 0.065]	0.080 [0.061 - 0.105]	0.057 [0.049 - 0.065]	0.079 [0.068 - 0.091]
1.5	0.042 [0.033 - 0.055]	0.044 [0.039 - 0.049]	0.055 [0.046 - 0.066]	0.079 [0.059 - 0.104]	0.057 [0.049 - 0.066]	0.079 [0.067 - 0.093]
2	0.042 [0.032 - 0.055]	0.044 [0.039 - 0.050]	0.055 [0.045 - 0.067]	0.077 [0.056 - 0.105]	0.058 [0.049 - 0.068]	0.080 [0.067 - 0.095]
2.5	0.042 [0.031 - 0.057]	0.044 [0.039 - 0.050]	0.055 [0.045 - 0.069]	0.075 [0.053 - 0.106]	0.059 [0.050 - 0.071]	0.080 [0.066 - 0.098]
3	0.043 [0.030 - 0.059]	0.044 [0.038 - 0.051]	0.056 [0.044 - 0.071]	0.074 [0.050 - 0.108]	0.062 [0.051 - 0.074]	0.081 [0.065 - 0.100]
3.5	0.044 [0.030 - 0.063]	0.045 [0.038 - 0.053]	0.058 [0.044 - 0.075]	0.073 [0.047 - 0.110]	0.064 [0.052 - 0.078]	0.081 [0.064 - 0.103]
4	0.045 [0.030 - 0.068]	0.045 [0.038 - 0.054]	0.059 [0.044 - 0.079]	0.071 [0.044 - 0.113]	0.067 [0.054 - 0.083]	0.082 [0.063 - 0.106]
4.5	0.047 [0.030 - 0.074]	0.046 [0.037 - 0.056]	0.062 [0.044 - 0.085]	0.070 [0.041 - 0.116]	0.070 [0.055 - 0.089]	0.083 [0.062 - 0.109]
5	0.050 [0.030 - 0.081]	0.046 [0.037 - 0.059]	0.064 [0.044 - 0.092]	0.069 [0.039 - 0.120]	0.073 [0.056 - 0.095]	0.083 [0.061 - 0.112]

1.5

Stillbirth

Age		<25 yrs			25-29 yrs	
Weight gain	Normal weight (n=1,324)	Underweight (n=698)	Overweight/Obese (n=314)	Normal weight (n=12,406)	Underweight (n=3,943)	Overweight/Obese (n=3,249)
-5	0.047 [0.013 - 0.157]	0.032 [0.012 - 0.081]	0.026 [0.006 - 0.104]	0.014 [0.006 - 0.032]	0.014 [0.009 - 0.022]	0.025 [0.014 - 0.045]
-4.5	0.041 [0.013 - 0.124]	0.029 [0.012 - 0.068]	0.027 [0.007 - 0.093]	0.013 [0.006 - 0.029]	0.013 [0.009 - 0.020]	0.024 [0.014 - 0.040]
-4	0.036 [0.013 - 0.098]	0.027 [0.012 - 0.057]	0.027 [0.008 - 0.084]	0.013 [0.006 - 0.026]	0.013 [0.009 - 0.018]	0.022 [0.014 - 0.036]
-3.5	0.032 [0.013 - 0.078]	0.025 [0.012 - 0.048]	0.027 [0.009 - 0.075]	0.012 [0.007 - 0.023]	0.012 [0.008 - 0.017]	0.021 [0.014 - 0.032]
-3	0.028 [0.012 - 0.062]	0.022 [0.012 - 0.041]	0.027 [0.010 - 0.068]	0.012 [0.007 - 0.021]	0.011 [0.008 - 0.015]	0.020 [0.013 - 0.029]
-2.5	0.024 [0.012 - 0.050]	0.020 [0.012 - 0.035]	0.027 [0.011 - 0.062]	0.012 [0.007 - 0.019]	0.011 [0.008 - 0.014]	0.018 [0.013 - 0.026]
-2	0.021 [0.011 - 0.041]	0.019 [0.011 - 0.031]	0.027 [0.012 - 0.058]	0.011 [0.007 - 0.017]	0.010 [0.008 - 0.013]	0.017 [0.013 - 0.024]
-1.5	0.019 [0.010 - 0.034]	0.017 [0.011 - 0.027]	0.027 [0.013 - 0.055]	0.011 [0.007 - 0.016]	0.010 [0.008 - 0.012]	0.016 [0.012 - 0.022]
-1	0.016 [0.009 - 0.030]	0.016 [0.010 - 0.024]	0.027 [0.013 - 0.053]	0.010 [0.007 - 0.015]	0.009 [0.007 - 0.011]	0.015 [0.011 - 0.020]
-0.5	0.014 [0.007 - 0.027]	0.014 [0.009 - 0.023]	0.027 [0.013 - 0.052]	0.010 [0.007 - 0.014]	0.009 [0.007 - 0.010]	0.014 [0.011 - 0.019]
Mean	0.012 [0.006 - 0.026]	0.013 [0.008 - 0.022]	0.027 [0.013 - 0.054]	0.009 [0.007 - 0.013]	0.008 [0.007 - 0.010]	0.013 [0.010 - 0.018]
0.5	0.011 [0.005 - 0.025]	0.012 [0.007 - 0.021]	0.027 [0.012 - 0.056]	0.009 [0.007 - 0.013]	0.008 [0.006 - 0.010]	0.012 [0.009 - 0.018]
1	0.010 [0.004 - 0.024]	0.011 [0.006 - 0.020]	0.027 [0.012 - 0.061]	0.009 [0.006 - 0.013]	0.007 [0.006 - 0.009]	0.012 [0.008 - 0.017]
1.5	0.008 [0.003 - 0.024]	0.010 [0.005 - 0.020]	0.027 [0.011 - 0.066]	0.008 [0.006 - 0.013]	0.007 [0.005 - 0.009]	0.011 [0.007 - 0.017]
2	0.007 [0.002 - 0.024]	0.009 [0.004 - 0.020]	0.027 [0.010 - 0.073]	0.008 [0.005 - 0.013]	0.007 [0.005 - 0.009]	0.010 [0.006 - 0.017]
2.5	0.006 [0.002 - 0.024]	0.008 [0.003 - 0.020]	0.027 [0.009 - 0.081]	0.008 [0.005 - 0.013]	0.006 [0.004 - 0.009]	0.010 [0.006 - 0.017]
3	0.006 [0.001 - 0.024]	0.007 [0.003 - 0.020]	0.027 [0.008 - 0.090]	0.008 [0.004 - 0.013]	0.006 [0.004 - 0.009]	0.009 [0.005 - 0.017]
3.5	0.005 [0.001 - 0.024]	0.007 [0.002 - 0.020]	0.027 [0.007 - 0.101]	0.007 [0.004 - 0.014]	0.006 [0.004 - 0.008]	0.008 [0.004 - 0.017]
4	0.004 [0.001 - 0.024]	0.006 [0.002 - 0.020]	0.027 [0.006 - 0.113]	0.007 [0.003 - 0.014]	0.005 [0.003 - 0.008]	0.008 [0.004 - 0.016]
4.5	0.004 [0.001 - 0.025]	0.006 [0.002 - 0.020]	0.027 [0.005 - 0.127]	0.007 [0.003 - 0.015]	0.005 [0.003 - 0.008]	0.007 [0.003 - 0.016]
5	0.003 [0.000 - 0.025]	0.005 [0.001 - 0.021]	0.027 [0.005 - 0.143]	0.006 [0.003 - 0.015]	0.005 [0.003 - 0.008]	0.007 [0.003 - 0.016]
Age		30-34 yrs			>34 yrs	
Weight gain	Normal weight (n=12,302)	Underweight (n=2,460)	Overweight/Obese (n=4,749)	Normal weight (n=5,750)	Underweight (n=704)	Overweight/Obese (n=3,221)
-5	0.010 [0.003 - 0.032]	0.016 [0.007 - 0.033]	0.013 [0.007 - 0.022]	0.001 [0.000 - 0.012]	0.022 [0.011 - 0.045]	0.019 [0.011 - 0.033]
-4.5	0.009 [0.003 - 0.028]	0.014 [0.008 - 0.026]	0.012 [0.007 - 0.021]	0.001 [0.000 - 0.012]	0.021 [0.011 - 0.038]	0.019 [0.011 - 0.031]
-4	0.009 [0.003 - 0.025]	0.013 [0.008 - 0.021]	0.012 [0.008 - 0.020]	0.002 [0.000 - 0.012]	0.019 [0.011 - 0.032]	0.019 [0.012 - 0.029]
-3.5	0.009 [0.004 - 0.022]	0.011 [0.008 - 0.018]	0.012 [0.008 - 0.019]	0.002 [0.000 - 0.013]	0.018 [0.011 - 0.028]	0.018 [0.012 - 0.028]
-3	0.009 [0.004 - 0.019]	0.010 [0.007 - 0.015]	0.012 [0.008 - 0.018]	0.002 [0.000 - 0.013]	0.017 [0.011 - 0.025]	0.018 [0.012 - 0.026]
-2.5	0.008 [0.004 - 0.017]	0.009 [0.007 - 0.013]	0.012 [0.009 - 0.017]	0.002 [0.000 - 0.013]	0.016 [0.011 - 0.022]	0.018 [0.013 - 0.025]
-2	0.008 [0.004 - 0.015]	0.009 [0.006 - 0.011]	0.012 [0.009 - 0.016]	0.003 [0.001 - 0.013]	0.014 [0.011 - 0.020]	0.018 [0.013 - 0.024]
-1.5	0.008 [0.004 - 0.014]	0.008 [0.006 - 0.010]	0.012 [0.009 - 0.016]	0.003 [0.001 - 0.014]	0.014 [0.010 - 0.018]	0.017 [0.013 - 0.023]
-1	0.008 [0.004 - 0.013]	0.007 [0.006 - 0.009]	0.012 [0.009 - 0.016]	0.004 [0.001 - 0.014]	0.013 [0.010 - 0.017]	0.017 [0.013 - 0.022]
-0.5	0.007 [0.005 - 0.012]	0.007 [0.005 - 0.009]	0.012 [0.009 - 0.016]	0.004 [0.001 - 0.015]	0.012 [0.009 - 0.016]	0.017 [0.013 - 0.022]
Mean	0.007 [0.004 - 0.011]	0.006 [0.005 - 0.008]	0.012 [0.009 - 0.016]	0.005 [0.002 - 0.015]	0.012 [0.009 - 0.015]	0.017 [0.012 - 0.022]
0.5	0.007 [0.004 - 0.011]	0.006 [0.005 - 0.008]	0.012 [0.009 - 0.016]	0.006 [0.002 - 0.016]	0.011 [0.008 - 0.015]	0.016 [0.012 - 0.022]
1	0.007 [0.004 - 0.011]	0.006 [0.004 - 0.008]	0.012 [0.008 - 0.016]	0.006 [0.002 - 0.017]	0.011 [0.008 - 0.014]	0.016 [0.011 - 0.023]
1.5	0.007 [0.004 - 0.012]	0.006 [0.004 - 0.008]	0.012 [0.008 - 0.017]	0.007 [0.003 - 0.019]	0.010 [0.008 - 0.014]	0.016 [0.011 - 0.023]
2	0.006 [0.003 - 0.012]	0.006 [0.004 - 0.008]	0.012 [0.008 - 0.017]	0.008 [0.003 - 0.020]	0.010 [0.007 - 0.014]	0.016 [0.010 - 0.024]
2.5	0.006 [0.003 - 0.013]	0.006 [0.004 - 0.009]	0.012 [0.007 - 0.018]	0.010 [0.004 - 0.023]	0.010 [0.007 - 0.015]	0.015 [0.010 - 0.025]
3	0.006 [0.003 - 0.013]	0.007 [0.005 - 0.010]	0.012 [0.007 - 0.019]	0.011 [0.005 - 0.026]	0.010 [0.006 - 0.015]	0.015 [0.009 - 0.025]
3.5	0.006 [0.002 - 0.014]	0.007 [0.005 - 0.011]	0.011 [0.007 - 0.020]	0.013 [0.005 - 0.031]	0.009 [0.006 - 0.015]	0.015 [0.009 - 0.026]
4	0.006 [0.002 - 0.015]	0.008 [0.005 - 0.012]	0.011 [0.006 - 0.021]	0.014 [0.006 - 0.037]	0.009 [0.005 - 0.016]	0.015 [0.008 - 0.027]
4.5	0.005 [0.002 - 0.016]	0.008 [0.005 - 0.014]	0.011 [0.006 - 0.022]	0.017 [0.006 - 0.044]	0.009 [0.005 - 0.016]	0.015 [0.007 - 0.028]
5	0.005 [0.002 - 0.018]	0.009 [0.005 - 0.016]	0.011 [0.006 - 0.023]	0.019 [0.006 - 0.054]	0.009 [0.004 - 0.017]	0.014 [0.007 - 0.030]

1.6

Macrosomia

Age		<25 yrs			25-29 yrs	
Weight gain	Normal weight (n=1,317)	Underweight (n=697)	Overweight/Obese (n=313)	Normal weight (n=12,390)	Underweight (n=3,940)	Overweight/Obese (n=3,236)
-5	0.006 [0.001 - 0.034]	0.017 [0.005 - 0.052]	0.083 [0.028 - 0.222]	0.001 [0.000 - 0.008]	0.032 [0.017 - 0.058]	0.043 [0.032 - 0.057]
-4.5	0.006 [0.001 - 0.032]	0.018 [0.007 - 0.049]	0.080 [0.032 - 0.186]	0.001 [0.000 - 0.008]	0.030 [0.018 - 0.047]	0.045 [0.034 - 0.059]
-4	0.007 [0.001 - 0.030]	0.020 [0.009 - 0.046]	0.076 [0.035 - 0.160]	0.002 [0.000 - 0.008]	0.028 [0.019 - 0.040]	0.048 [0.037 - 0.061]
-3.5	0.007 [0.002 - 0.028]	0.022 [0.011 - 0.045]	0.073 [0.037 - 0.140]	0.002 [0.001 - 0.008]	0.026 [0.019 - 0.035]	0.050 [0.040 - 0.063]
-3	0.008 [0.002 - 0.027]	0.025 [0.014 - 0.044]	0.070 [0.038 - 0.125]	0.003 [0.001 - 0.008]	0.025 [0.019 - 0.032]	0.053 [0.043 - 0.065]
-2.5	0.008 [0.003 - 0.026]	0.027 [0.016 - 0.044]	0.068 [0.039 - 0.115]	0.004 [0.002 - 0.009]	0.024 [0.020 - 0.030]	0.056 [0.046 - 0.067]
-2	0.009 [0.003 - 0.024]	0.029 [0.019 - 0.045]	0.066 [0.039 - 0.109]	0.005 [0.003 - 0.010]	0.025 [0.021 - 0.030]	0.059 [0.050 - 0.070]
-1.5	0.009 [0.004 - 0.024]	0.032 [0.022 - 0.047]	0.065 [0.039 - 0.105]	0.007 [0.004 - 0.012]	0.026 [0.022 - 0.031]	0.062 [0.053 - 0.072]
-1	0.010 [0.004 - 0.023]	0.034 [0.024 - 0.049]	0.065 [0.040 - 0.105]	0.009 [0.006 - 0.014]	0.029 [0.024 - 0.033]	0.066 [0.057 - 0.075]
-0.5	0.011 [0.005 - 0.023]	0.037 [0.026 - 0.052]	0.066 [0.041 - 0.107]	0.011 [0.008 - 0.016]	0.031 [0.027 - 0.036]	0.069 [0.061 - 0.079]
Mean	0.011 [0.006 - 0.023]	0.039 [0.028 - 0.055]	0.069 [0.042 - 0.112]	0.014 [0.010 - 0.019]	0.035 [0.030 - 0.040]	0.073 [0.064 - 0.083]
0.5	0.012 [0.006 - 0.024]	0.042 [0.030 - 0.059]	0.074 [0.045 - 0.120]	0.016 [0.012 - 0.023]	0.038 [0.033 - 0.043]	0.077 [0.068 - 0.087]
1	0.013 [0.007 - 0.026]	0.044 [0.031 - 0.062]	0.080 [0.048 - 0.132]	0.019 [0.014 - 0.026]	0.040 [0.035 - 0.046]	0.081 [0.071 - 0.092]
1.5	0.014 [0.007 - 0.029]	0.046 [0.032 - 0.066]	0.088 [0.051 - 0.147]	0.022 [0.016 - 0.030]	0.042 [0.036 - 0.049]	0.085 [0.074 - 0.098]
2	0.015 [0.007 - 0.033]	0.048 [0.033 - 0.071]	0.098 [0.056 - 0.165]	0.024 [0.017 - 0.033]	0.044 [0.038 - 0.052]	0.090 [0.077 - 0.104]
2.5	0.016 [0.007 - 0.038]	0.050 [0.033 - 0.076]	0.108 [0.060 - 0.188]	0.027 [0.019 - 0.037]	0.047 [0.039 - 0.055]	0.095 [0.080 - 0.111]
3	0.017 [0.007 - 0.044]	0.051 [0.032 - 0.080]	0.120 [0.064 - 0.215]	0.029 [0.020 - 0.042]	0.050 [0.042 - 0.060]	0.099 [0.083 - 0.119]
3.5	0.018 [0.006 - 0.052]	0.051 [0.030 - 0.085]	0.133 [0.066 - 0.247]	0.031 [0.021 - 0.046]	0.055 [0.046 - 0.067]	0.105 [0.086 - 0.127]
4	0.020 [0.006 - 0.061]	0.051 [0.028 - 0.090]	0.146 [0.068 - 0.284]	0.034 [0.022 - 0.051]	0.062 [0.050 - 0.077]	0.110 [0.089 - 0.136]
4.5	0.021 [0.006 - 0.073]	0.050 [0.026 - 0.096]	0.159 [0.069 - 0.326]	0.036 [0.023 - 0.057]	0.070 [0.056 - 0.088]	0.116 [0.092 - 0.145]
5	0.022 [0.005 - 0.087]	0.049 [0.023 - 0.101]	0.173 [0.068 - 0.374]	0.038 [0.023 - 0.064]	0.080 [0.062 - 0.102]	0.122 [0.094 - 0.156]
Age		30-34 yrs			>34 yrs	
Weight gain	Normal weight (n=12,281)	Underweight (n=2,458)	Overweight/Obese (n=4,738)	Normal weight (n=5,735)	Underweight (n=703)	Overweight/Obese (n=3,211)
-5	0.004 [0.001 - 0.011]	0.014 [0.009 - 0.021]	0.039 [0.027 - 0.058]	0.000 [0.000 - 0.008]	0.014 [0.010 - 0.019]	0.036 [0.027 - 0.049]
-4.5	0.004 [0.002 - 0.011]	0.016 [0.011 - 0.022]	0.042 [0.030 - 0.058]	0.000 [0.000 - 0.008]	0.015 [0.011 - 0.021]	0.039 [0.029 - 0.051]
-4	0.005 [0.002 - 0.012]	0.017 [0.013 - 0.023]	0.045 [0.034 - 0.058]	0.000 [0.000 - 0.008]	0.016 [0.012 - 0.022]	0.041 [0.032 - 0.053]
-3.5	0.006 [0.003 - 0.012]	0.019 [0.015 - 0.025]	0.047 [0.038 - 0.060]	0.000 [0.000 - 0.008]	0.018 [0.013 - 0.023]	0.043 [0.034 - 0.055]
-3	0.006 [0.003 - 0.013]	0.021 [0.017 - 0.026]	0.051 [0.042 - 0.061]	0.000 [0.000 - 0.008]	0.019 [0.015 - 0.025]	0.046 [0.037 - 0.057]
-2.5	0.007 [0.004 - 0.014]	0.024 [0.020 - 0.028]	0.054 [0.046 - 0.064]	0.001 [0.000 - 0.008]	0.021 [0.016 - 0.026]	0.049 [0.040 - 0.059]
-2	0.008 [0.005 - 0.015]	0.026 [0.022 - 0.030]	0.058 [0.050 - 0.068]	0.001 [0.000 - 0.008]	0.023 [0.018 - 0.028]	0.052 [0.043 - 0.061]
-1.5	0.010 [0.006 - 0.016]	0.029 [0.025 - 0.033]	0.063 [0.055 - 0.072]	0.001 [0.000 - 0.009]	0.024 [0.020 - 0.030]	0.055 [0.046 - 0.064]
-1	0.011 [0.007 - 0.017]	0.031 [0.028 - 0.035]	0.067 [0.059 - 0.077]	0.001 [0.000 - 0.010]	0.027 [0.022 - 0.032]	0.058 [0.050 - 0.067]
-0.5	0.012 [0.008 - 0.018]	0.034 [0.031 - 0.038]	0.072 [0.063 - 0.082]	0.002 [0.000 - 0.010]	0.029 [0.025 - 0.034]	0.061 [0.053 - 0.070]
Mean	0.014 [0.010 - 0.020]	0.037 [0.034 - 0.042]	0.076 [0.067 - 0.087]	0.003 [0.001 - 0.012]	0.031 [0.027 - 0.037]	0.065 [0.056 - 0.074]
0.5	0.016 [0.011 - 0.022]	0.041 [0.037 - 0.045]	0.080 [0.070 - 0.092]	0.003 [0.001 - 0.013]	0.034 [0.030 - 0.039]	0.069 [0.060 - 0.079]
1	0.018 [0.013 - 0.025]	0.044 [0.040 - 0.049]	0.084 [0.073 - 0.097]	0.004 [0.001 - 0.016]	0.037 [0.032 - 0.043]	0.073 [0.063 - 0.084]
1.5	0.020 [0.015 - 0.028]	0.047 [0.043 - 0.053]	0.087 [0.075 - 0.101]	0.006 [0.002 - 0.019]	0.040 [0.035 - 0.046]	0.077 [0.066 - 0.090]
2	0.023 [0.017 - 0.032]	0.051 [0.045 - 0.057]	0.090 [0.077 - 0.106]	0.008 [0.003 - 0.023]	0.044 [0.038 - 0.050]	0.081 [0.068 - 0.096]
2.5	0.026 [0.019 - 0.036]	0.055 [0.048 - 0.062]	0.093 [0.078 - 0.111]	0.010 [0.004 - 0.028]	0.047 [0.041 - 0.055]	0.086 [0.071 - 0.103]
3	0.029 [0.020 - 0.041]	0.059 [0.051 - 0.067]	0.096 [0.079 - 0.116]	0.013 [0.005 - 0.035]	0.051 [0.043 - 0.060]	0.091 [0.074 - 0.111]
3.5	0.033 [0.022 - 0.048]	0.062 [0.054 - 0.072]	0.098 [0.079 - 0.121]	0.017 [0.007 - 0.043]	0.056 [0.046 - 0.066]	0.096 [0.077 - 0.119]
4	0.036 [0.024 - 0.055]	0.066 [0.057 - 0.077]	0.100 [0.079 - 0.126]	0.021 [0.008 - 0.053]	0.060 [0.049 - 0.073]	0.101 [0.079 - 0.128]
4.5	0.040 [0.026 - 0.063]	0.070 [0.059 - 0.083]	0.102 [0.078 - 0.132]	0.027 [0.010 - 0.066]	0.065 [0.053 - 0.081]	0.107 [0.082 - 0.138]
5	0.045 [0.027 - 0.073]	0.074 [0.061 - 0.090]	0.104 [0.076 - 0.139]	0.033 [0.013 - 0.082]	0.071 [0.056 - 0.089]	0.113 [0.085 - 0.149]

1.7

Small for gestational age

Age		<25 yrs			25-29 yrs	
Weight gain	Normal weight (n=1,316)	Underweight (n=697)	Overweight/Obese (n=313)	Normal weight (n=12,386)	Underweight (n=3,938)	Overweight/Obese (n=3,236)
-5	0.153 [0.083 - 0.266]	0.067 [0.037 - 0.120]	0.056 [0.017 - 0.167]	0.061 [0.031 - 0.116]	0.080 [0.064 - 0.098]	0.032 [0.019 - 0.052]
-4.5	0.140 [0.080 - 0.233]	0.063 [0.036 - 0.107]	0.052 [0.018 - 0.143]	0.061 [0.035 - 0.105]	0.073 [0.060 - 0.088]	0.031 [0.019 - 0.048]
-4	0.127 [0.077 - 0.204]	0.059 [0.036 - 0.096]	0.049 [0.019 - 0.122]	0.062 [0.040 - 0.096]	0.067 [0.056 - 0.079]	0.029 [0.019 - 0.044]
-3.5	0.116 [0.074 - 0.178]	0.055 [0.035 - 0.086]	0.046 [0.020 - 0.105]	0.063 [0.044 - 0.089]	0.061 [0.052 - 0.071]	0.028 [0.019 - 0.040]
-3	0.105 [0.070 - 0.155]	0.052 [0.035 - 0.077]	0.043 [0.020 - 0.091]	0.064 [0.047 - 0.084]	0.056 [0.048 - 0.064]	0.026 [0.019 - 0.037]
-2.5	0.096 [0.067 - 0.135]	0.049 [0.034 - 0.069]	0.041 [0.020 - 0.080]	0.064 [0.051 - 0.081]	0.051 [0.045 - 0.058]	0.025 [0.019 - 0.034]
-2	0.087 [0.063 - 0.119]	0.046 [0.033 - 0.063]	0.038 [0.020 - 0.071]	0.064 [0.053 - 0.079]	0.046 [0.041 - 0.052]	0.024 [0.018 - 0.031]
-1.5	0.079 [0.058 - 0.105]	0.043 [0.032 - 0.057]	0.036 [0.019 - 0.065]	0.064 [0.053 - 0.076]	0.042 [0.038 - 0.047]	0.023 [0.018 - 0.029]
-1	0.071 [0.053 - 0.095]	0.040 [0.030 - 0.053]	0.033 [0.018 - 0.061]	0.062 [0.052 - 0.073]	0.039 [0.035 - 0.042]	0.022 [0.017 - 0.028]
-0.5	0.064 [0.048 - 0.086]	0.037 [0.028 - 0.050]	0.031 [0.016 - 0.060]	0.059 [0.050 - 0.070]	0.035 [0.032 - 0.039]	0.021 [0.016 - 0.027]
Mean	0.058 [0.042 - 0.080]	0.035 [0.026 - 0.047]	0.029 [0.014 - 0.059]	0.056 [0.047 - 0.066]	0.032 [0.029 - 0.036]	0.020 [0.015 - 0.026]
0.5	0.052 [0.037 - 0.075]	0.033 [0.023 - 0.045]	0.028 [0.012 - 0.060]	0.052 [0.044 - 0.062]	0.029 [0.026 - 0.033]	0.019 [0.014 - 0.025]
1	0.047 [0.031 - 0.071]	0.031 [0.021 - 0.044]	0.026 [0.010 - 0.062]	0.049 [0.040 - 0.059]	0.027 [0.024 - 0.030]	0.018 [0.013 - 0.025]
1.5	0.043 [0.027 - 0.067]	0.029 [0.019 - 0.043]	0.024 [0.009 - 0.064]	0.045 [0.037 - 0.056]	0.024 [0.021 - 0.028]	0.017 [0.012 - 0.024]
2	0.039 [0.023 - 0.065]	0.027 [0.017 - 0.042]	0.023 [0.007 - 0.067]	0.042 [0.034 - 0.053]	0.022 [0.019 - 0.026]	0.016 [0.011 - 0.024]
2.5	0.035 [0.019 - 0.062]	0.025 [0.015 - 0.042]	0.021 [0.006 - 0.071]	0.040 [0.031 - 0.051]	0.020 [0.017 - 0.024]	0.016 [0.010 - 0.024]
3	0.031 [0.016 - 0.060]	0.023 [0.013 - 0.041]	0.020 [0.005 - 0.075]	0.038 [0.028 - 0.050]	0.018 [0.015 - 0.022]	0.015 [0.009 - 0.024]
3.5	0.028 [0.014 - 0.057]	0.022 [0.011 - 0.041]	0.019 [0.004 - 0.079]	0.036 [0.026 - 0.050]	0.017 [0.013 - 0.021]	0.014 [0.008 - 0.024]
4	0.025 [0.011 - 0.055]	0.020 [0.010 - 0.041]	0.017 [0.003 - 0.083]	0.035 [0.024 - 0.050]	0.015 [0.012 - 0.019]	0.014 [0.008 - 0.024]
4.5	0.023 [0.010 - 0.053]	0.019 [0.009 - 0.040]	0.016 [0.003 - 0.089]	0.033 [0.022 - 0.051]	0.014 [0.011 - 0.018]	0.013 [0.007 - 0.024]
5	0.021 [0.008 - 0.052]	0.018 [0.008 - 0.040]	0.015 [0.002 - 0.094]	0.032 [0.019 - 0.053]	0.013 [0.009 - 0.017]	0.012 [0.006 - 0.024]
Age		30-34 yrs			>34 yrs	
Weight gain	Normal weight (n=12,275)	Underweight (n=2,458)	Overweight/Obese (n=4,735)	Normal weight (n=5,730)	Underweight (n=703)	Overweight/Obese (n=3,208)
-5	0.119 [0.080 - 0.174]	0.080 [0.058 - 0.110]	0.048 [0.033 - 0.069]	0.190 [0.095 - 0.344]	0.054 [0.037 - 0.078]	0.042 [0.023 - 0.076]
-4.5	0.111 [0.077 - 0.157]	0.072 [0.054 - 0.094]	0.043 [0.031 - 0.060]	0.170 [0.090 - 0.297]	0.050 [0.036 - 0.070]	0.037 [0.022 - 0.060]
-4	0.103 [0.074 - 0.142]	0.064 [0.051 - 0.080]	0.039 [0.029 - 0.053]	0.151 [0.084 - 0.254]	0.046 [0.034 - 0.062]	0.032 [0.021 - 0.048]
-3.5	0.096 [0.071 - 0.128]	0.058 [0.048 - 0.069]	0.036 [0.027 - 0.046]	0.133 [0.079 - 0.216]	0.043 [0.033 - 0.056]	0.028 [0.019 - 0.040]
-3	0.089 [0.068 - 0.115]	0.052 [0.044 - 0.061]	0.032 [0.025 - 0.041]	0.118 [0.074 - 0.183]	0.040 [0.031 - 0.050]	0.025 [0.018 - 0.034]
-2.5	0.083 [0.065 - 0.104]	0.047 [0.041 - 0.054]	0.029 [0.024 - 0.036]	0.104 [0.068 - 0.155]	0.037 [0.029 - 0.045]	0.022 [0.016 - 0.030]
-2	0.077 [0.062 - 0.094]	0.042 [0.037 - 0.048]	0.026 [0.022 - 0.032]	0.091 [0.063 - 0.131]	0.034 [0.028 - 0.041]	0.020 [0.014 - 0.027]
-1.5	0.071 [0.059 - 0.085]	0.038 [0.034 - 0.043]	0.024 [0.020 - 0.029]	0.080 [0.057 - 0.112]	0.031 [0.026 - 0.037]	0.018 [0.013 - 0.024]
-1	0.066 [0.055 - 0.078]	0.035 [0.031 - 0.039]	0.022 [0.018 - 0.026]	0.070 [0.051 - 0.096]	0.029 [0.024 - 0.034]	0.017 [0.012 - 0.023]
-0.5	0.061 [0.052 - 0.072]	0.032 [0.028 - 0.036]	0.019 [0.016 - 0.024]	0.062 [0.045 - 0.084]	0.027 [0.023 - 0.031]	0.016 [0.011 - 0.022]
Mean	0.056 [0.048 - 0.066]	0.029 [0.026 - 0.033]	0.018 [0.014 - 0.022]	0.054 [0.039 - 0.075]	0.025 [0.021 - 0.029]	0.015 [0.011 - 0.021]
0.5	0.052 [0.044 - 0.062]	0.027 [0.024 - 0.031]	0.016 [0.012 - 0.021]	0.047 [0.033 - 0.068]	0.023 [0.019 - 0.027]	0.015 [0.010 - 0.021]
1	0.048 [0.040 - 0.059]	0.025 [0.022 - 0.029]	0.014 [0.011 - 0.020]	0.041 [0.027 - 0.062]	0.021 [0.017 - 0.026]	0.014 [0.010 - 0.021]
1.5	0.045 [0.036 - 0.056]	0.023 [0.020 - 0.027]	0.013 [0.009 - 0.018]	0.036 [0.022 - 0.057]	0.019 [0.015 - 0.024]	0.014 [0.009 - 0.022]
2	0.041 [0.032 - 0.053]	0.022 [0.019 - 0.026]	0.012 [0.008 - 0.017]	0.031 [0.018 - 0.053]	0.018 [0.014 - 0.023]	0.014 [0.009 - 0.022]
2.5	0.038 [0.028 - 0.051]	0.021 [0.017 - 0.026]	0.011 [0.007 - 0.016]	0.027 [0.015 - 0.050]	0.016 [0.012 - 0.022]	0.014 [0.008 - 0.023]
3	0.035 [0.025 - 0.049]	0.020 [0.016 - 0.025]	0.010 [0.006 - 0.015]	0.024 [0.012 - 0.046]	0.015 [0.011 - 0.021]	0.014 [0.008 - 0.024]
3.5	0.033 [0.023 - 0.047]	0.019 [0.015 - 0.025]	0.009 [0.005 - 0.015]	0.021 [0.010 - 0.044]	0.014 [0.010 - 0.020]	0.013 [0.007 - 0.026]
4	0.030 [0.020 - 0.045]	0.018 [0.014 - 0.025]	0.008 [0.004 - 0.014]	0.018 [0.008 - 0.041]	0.013 [0.009 - 0.019]	0.013 [0.006 - 0.028]
4.5	0.028 [0.018 - 0.043]	0.018 [0.012 - 0.025]	0.007 [0.004 - 0.013]	0.016 [0.006 - 0.038]	0.012 [0.008 - 0.018]	0.013 [0.006 - 0.030]
5	0.026 [0.016 - 0.041]	0.017 [0.011 - 0.025]	0.006 [0.003 - 0.012]	0.014 [0.005 - 0.036]	0.011 [0.007 - 0.018]	0.013 [0.005 - 0.034]

1.8

Large for gestational age

Age		<25 yrs			25-29 yrs	
Weight gain	Normal weight (n=1,316)	Underweight (n=697)	Overweight/Obese (n=313)	Normal weight (n=12,386)	Underweight (n=3,938)	Overweight/Obese (n=3,236)
-5	0.038 [0.019 - 0.076]	0.084 [0.058 - 0.120]	0.153 [0.089 - 0.250]	0.032 [0.019 - 0.053]	0.064 [0.056 - 0.073]	0.132 [0.111 - 0.156]
-4.5	0.041 [0.022 - 0.077]	0.088 [0.063 - 0.122]	0.157 [0.096 - 0.246]	0.035 [0.022 - 0.054]	0.068 [0.060 - 0.077]	0.138 [0.118 - 0.161]
-4	0.044 [0.024 - 0.079]	0.091 [0.067 - 0.123]	0.161 [0.103 - 0.242]	0.038 [0.026 - 0.056]	0.073 [0.065 - 0.081]	0.145 [0.126 - 0.166]
-3.5	0.047 [0.028 - 0.080]	0.095 [0.072 - 0.125]	0.165 [0.111 - 0.239]	0.042 [0.030 - 0.058]	0.078 [0.070 - 0.086]	0.152 [0.133 - 0.172]
-3	0.051 [0.031 - 0.082]	0.099 [0.077 - 0.127]	0.169 [0.118 - 0.236]	0.045 [0.034 - 0.060]	0.083 [0.075 - 0.091]	0.159 [0.142 - 0.178]
-2.5	0.054 [0.035 - 0.084]	0.103 [0.082 - 0.129]	0.173 [0.126 - 0.234]	0.049 [0.039 - 0.062]	0.088 [0.081 - 0.096]	0.166 [0.150 - 0.184]
-2	0.058 [0.039 - 0.086]	0.108 [0.088 - 0.131]	0.178 [0.133 - 0.233]	0.054 [0.044 - 0.066]	0.094 [0.087 - 0.101]	0.174 [0.159 - 0.191]
-1.5	0.062 [0.044 - 0.088]	0.112 [0.093 - 0.134]	0.182 [0.140 - 0.234]	0.059 [0.050 - 0.070]	0.100 [0.093 - 0.106]	0.182 [0.168 - 0.198]
-1	0.067 [0.048 - 0.091]	0.116 [0.098 - 0.137]	0.187 [0.146 - 0.236]	0.064 [0.055 - 0.074]	0.106 [0.100 - 0.112]	0.191 [0.177 - 0.206]
-0.5	0.071 [0.053 - 0.095]	0.121 [0.104 - 0.141]	0.191 [0.150 - 0.240]	0.069 [0.061 - 0.079]	0.113 [0.107 - 0.119]	0.199 [0.185 - 0.214]
Mean	0.076 [0.058 - 0.100]	0.126 [0.109 - 0.146]	0.196 [0.154 - 0.246]	0.075 [0.066 - 0.085]	0.120 [0.114 - 0.126]	0.208 [0.194 - 0.223]
0.5	0.082 [0.063 - 0.106]	0.131 [0.113 - 0.152]	0.201 [0.155 - 0.255]	0.081 [0.071 - 0.092]	0.127 [0.121 - 0.133]	0.217 [0.203 - 0.233]
1	0.087 [0.067 - 0.113]	0.136 [0.117 - 0.158]	0.205 [0.156 - 0.266]	0.087 [0.076 - 0.098]	0.135 [0.129 - 0.141]	0.227 [0.211 - 0.244]
1.5	0.093 [0.071 - 0.122]	0.142 [0.120 - 0.166]	0.210 [0.155 - 0.278]	0.092 [0.081 - 0.105]	0.143 [0.136 - 0.150]	0.237 [0.219 - 0.256]
2	0.100 [0.074 - 0.133]	0.147 [0.123 - 0.175]	0.215 [0.154 - 0.292]	0.098 [0.086 - 0.112]	0.152 [0.144 - 0.160]	0.247 [0.227 - 0.268]
2.5	0.107 [0.077 - 0.146]	0.153 [0.126 - 0.185]	0.220 [0.152 - 0.308]	0.104 [0.090 - 0.120]	0.161 [0.151 - 0.170]	0.257 [0.234 - 0.282]
3	0.114 [0.079 - 0.161]	0.159 [0.128 - 0.196]	0.226 [0.150 - 0.324]	0.109 [0.093 - 0.127]	0.170 [0.159 - 0.182]	0.268 [0.242 - 0.296]
3.5	0.121 [0.081 - 0.177]	0.165 [0.130 - 0.207]	0.231 [0.148 - 0.342]	0.115 [0.097 - 0.136]	0.180 [0.168 - 0.193]	0.279 [0.250 - 0.310]
4	0.129 [0.083 - 0.195]	0.171 [0.132 - 0.220]	0.236 [0.145 - 0.361]	0.121 [0.100 - 0.145]	0.190 [0.176 - 0.206]	0.290 [0.257 - 0.325]
4.5	0.138 [0.085 - 0.215]	0.178 [0.134 - 0.232]	0.242 [0.142 - 0.380]	0.127 [0.103 - 0.155]	0.201 [0.185 - 0.219]	0.302 [0.265 - 0.341]
5	0.147 [0.087 - 0.237]	0.185 [0.136 - 0.246]	0.247 [0.139 - 0.400]	0.133 [0.105 - 0.166]	0.212 [0.194 - 0.233]	0.313 [0.273 - 0.357]
Age		30-34 yrs			>34 yrs	
Weight gain	Normal weight (n=12,275)	Underweight (n=2,458)	Overweight/Obese (n=4,735)	Normal weight (n=5,730)	Underweight (n=703)	Overweight/Obese (n=3,208)
-5	0.057 [0.035 - 0.093]	0.061 [0.048 - 0.078]	0.151 [0.132 - 0.172]	0.038 [0.020 - 0.072]	0.093 [0.079 - 0.110]	0.192 [0.166 - 0.221]
-4.5	0.059 [0.038 - 0.091]	0.067 [0.054 - 0.082]	0.157 [0.139 - 0.177]	0.041 [0.023 - 0.074]	0.098 [0.084 - 0.114]	0.197 [0.173 - 0.224]
-4	0.061 [0.042 - 0.089]	0.073 [0.061 - 0.086]	0.163 [0.146 - 0.182]	0.045 [0.026 - 0.077]	0.103 [0.090 - 0.118]	0.202 [0.179 - 0.226]
-3.5	0.063 [0.045 - 0.088]	0.079 [0.068 - 0.091]	0.170 [0.154 - 0.187]	0.049 [0.029 - 0.080]	0.108 [0.096 - 0.123]	0.207 [0.186 - 0.229]
-3	0.065 [0.049 - 0.087]	0.086 [0.076 - 0.096]	0.177 [0.162 - 0.193]	0.053 [0.033 - 0.083]	0.114 [0.102 - 0.128]	0.212 [0.192 - 0.232]
-2.5	0.068 [0.053 - 0.087]	0.093 [0.085 - 0.102]	0.184 [0.170 - 0.199]	0.057 [0.037 - 0.087]	0.120 [0.108 - 0.133]	0.217 [0.199 - 0.236]
-2	0.070 [0.057 - 0.087]	0.101 [0.093 - 0.109]	0.191 [0.178 - 0.205]	0.062 [0.042 - 0.091]	0.126 [0.115 - 0.138]	0.222 [0.205 - 0.239]
-1.5	0.073 [0.061 - 0.088]	0.109 [0.101 - 0.116]	0.199 [0.187 - 0.212]	0.067 [0.047 - 0.094]	0.132 [0.121 - 0.143]	0.227 [0.211 - 0.243]
-1	0.076 [0.065 - 0.090]	0.117 [0.110 - 0.124]	0.207 [0.195 - 0.219]	0.072 [0.052 - 0.099]	0.138 [0.129 - 0.149]	0.232 [0.217 - 0.248]
-0.5	0.080 [0.068 - 0.093]	0.125 [0.118 - 0.132]	0.215 [0.203 - 0.227]	0.078 [0.058 - 0.104]	0.145 [0.136 - 0.155]	0.238 [0.223 - 0.253]
Mean	0.084 [0.072 - 0.096]	0.133 [0.125 - 0.140]	0.223 [0.211 - 0.236]	0.084 [0.064 - 0.109]	0.152 [0.143 - 0.162]	0.243 [0.228 - 0.259]
0.5	0.088 [0.076 - 0.101]	0.140 [0.133 - 0.149]	0.231 [0.218 - 0.245]	0.091 [0.071 - 0.116]	0.160 [0.150 - 0.170]	0.249 [0.233 - 0.266]
1	0.092 [0.080 - 0.106]	0.148 [0.140 - 0.157]	0.240 [0.225 - 0.255]	0.098 [0.077 - 0.123]	0.167 [0.157 - 0.178]	0.255 [0.237 - 0.273]
1.5	0.098 [0.084 - 0.113]	0.155 [0.146 - 0.165]	0.249 [0.233 - 0.266]	0.105 [0.084 - 0.132]	0.175 [0.164 - 0.187]	0.260 [0.240 - 0.281]
2	0.103 [0.088 - 0.120]	0.163 [0.152 - 0.173]	0.258 [0.239 - 0.277]	0.114 [0.090 - 0.143]	0.183 [0.171 - 0.196]	0.266 [0.244 - 0.290]
2.5	0.109 [0.092 - 0.129]	0.170 [0.159 - 0.182]	0.267 [0.246 - 0.289]	0.122 [0.096 - 0.155]	0.192 [0.178 - 0.206]	0.272 [0.247 - 0.299]
3	0.116 [0.096 - 0.138]	0.178 [0.165 - 0.191]	0.277 [0.253 - 0.302]	0.132 [0.101 - 0.169]	0.201 [0.185 - 0.217]	0.278 [0.250 - 0.308]
3.5	0.123 [0.101 - 0.149]	0.185 [0.170 - 0.201]	0.286 [0.260 - 0.315]	0.141 [0.106 - 0.186]	0.210 [0.192 - 0.229]	0.284 [0.253 - 0.318]
4	0.130 [0.105 - 0.161]	0.193 [0.176 - 0.211]	0.296 [0.267 - 0.328]	0.152 [0.111 - 0.203]	0.219 [0.199 - 0.241]	0.290 [0.256 - 0.328]
4.5	0.138 [0.109 - 0.174]	0.200 [0.181 - 0.221]	0.307 [0.273 - 0.342]	0.163 [0.116 - 0.223]	0.229 [0.206 - 0.254]	0.297 [0.259 - 0.338]
5	0.147 [0.113 - 0.189]	0.208 [0.185 - 0.232]	0.317 [0.280 - 0.356]	0.175 [0.121 - 0.245]	0.239 [0.213 - 0.267]	0.303 [0.262 - 0.348]

1.9

All disease

Age		<25 yrs			25-29 yrs	
Weight gain	Normal weight (n=1,324)	Underweight (n=698)	Overweight/Obese (n=314)	Normal weight (n=12,406)	Underweight (n=3,943)	Overweight/Obese (n=3,249)
-5	0.268 [0.177 - 0.384]	0.249 [0.200 - 0.305]	0.473 [0.324 - 0.628]	0.217 [0.186 - 0.251]	0.319 [0.270 - 0.372]	0.429 [0.379 - 0.481]
-4.5	0.264 [0.184 - 0.364]	0.253 [0.207 - 0.305]	0.466 [0.339 - 0.597]	0.219 [0.191 - 0.252]	0.307 [0.270 - 0.347]	0.433 [0.390 - 0.477]
-4	0.260 [0.190 - 0.345]	0.257 [0.214 - 0.305]	0.459 [0.351 - 0.570]	0.223 [0.196 - 0.252]	0.297 [0.269 - 0.326]	0.436 [0.400 - 0.474]
-3.5	0.257 [0.196 - 0.329]	0.261 [0.222 - 0.305]	0.451 [0.359 - 0.547]	0.226 [0.201 - 0.253]	0.287 [0.266 - 0.310]	0.440 [0.409 - 0.472]
-3	0.253 [0.200 - 0.314]	0.265 [0.229 - 0.305]	0.445 [0.364 - 0.529]	0.229 [0.206 - 0.253]	0.280 [0.262 - 0.299]	0.444 [0.416 - 0.471]
-2.5	0.249 [0.203 - 0.302]	0.269 [0.236 - 0.305]	0.439 [0.366 - 0.515]	0.232 [0.211 - 0.254]	0.275 [0.260 - 0.291]	0.447 [0.423 - 0.471]
-2	0.246 [0.205 - 0.292]	0.274 [0.243 - 0.306]	0.435 [0.367 - 0.506]	0.235 [0.216 - 0.255]	0.274 [0.260 - 0.288]	0.451 [0.429 - 0.473]
-1.5	0.243 [0.205 - 0.284]	0.278 [0.250 - 0.308]	0.433 [0.366 - 0.501]	0.238 [0.221 - 0.256]	0.275 [0.262 - 0.288]	0.454 [0.434 - 0.475]
-1	0.240 [0.205 - 0.279]	0.282 [0.256 - 0.310]	0.432 [0.367 - 0.500]	0.241 [0.226 - 0.257]	0.278 [0.266 - 0.290]	0.458 [0.438 - 0.478]
-0.5	0.239 [0.205 - 0.276]	0.287 [0.262 - 0.313]	0.434 [0.368 - 0.503]	0.244 [0.230 - 0.259]	0.283 [0.271 - 0.295]	0.462 [0.442 - 0.482]
Mean	0.238 [0.204 - 0.275]	0.291 [0.266 - 0.317]	0.439 [0.370 - 0.510]	0.248 [0.234 - 0.262]	0.289 [0.277 - 0.301]	0.466 [0.446 - 0.487]
0.5	0.238 [0.203 - 0.276]	0.296 [0.270 - 0.322]	0.446 [0.375 - 0.520]	0.251 [0.237 - 0.265]	0.296 [0.283 - 0.308]	0.471 [0.450 - 0.493]
1	0.238 [0.202 - 0.279]	0.300 [0.273 - 0.329]	0.457 [0.380 - 0.535]	0.254 [0.240 - 0.269]	0.303 [0.290 - 0.316]	0.477 [0.454 - 0.500]
1.5	0.239 [0.200 - 0.283]	0.305 [0.274 - 0.337]	0.469 [0.386 - 0.553]	0.258 [0.242 - 0.274]	0.311 [0.297 - 0.326]	0.483 [0.458 - 0.508]
2	0.241 [0.198 - 0.289]	0.309 [0.276 - 0.345]	0.482 [0.392 - 0.574]	0.261 [0.244 - 0.279]	0.320 [0.305 - 0.336]	0.489 [0.462 - 0.516]
2.5	0.243 [0.196 - 0.296]	0.314 [0.276 - 0.354]	0.497 [0.397 - 0.598]	0.264 [0.245 - 0.284]	0.329 [0.312 - 0.347]	0.495 [0.466 - 0.525]
3	0.245 [0.193 - 0.305]	0.318 [0.277 - 0.363]	0.513 [0.401 - 0.624]	0.268 [0.246 - 0.291]	0.339 [0.320 - 0.359]	0.502 [0.470 - 0.535]
3.5	0.247 [0.190 - 0.315]	0.323 [0.277 - 0.373]	0.530 [0.403 - 0.653]	0.271 [0.247 - 0.297]	0.349 [0.328 - 0.372]	0.510 [0.474 - 0.546]
4	0.249 [0.185 - 0.327]	0.328 [0.277 - 0.383]	0.547 [0.402 - 0.685]	0.275 [0.248 - 0.304]	0.360 [0.335 - 0.386]	0.518 [0.478 - 0.558]
4.5	0.252 [0.180 - 0.341]	0.333 [0.277 - 0.394]	0.565 [0.399 - 0.717]	0.278 [0.248 - 0.310]	0.371 [0.343 - 0.401]	0.527 [0.482 - 0.572]
5	0.255 [0.174 - 0.356]	0.337 [0.276 - 0.404]	0.583 [0.395 - 0.749]	0.282 [0.249 - 0.317]	0.382 [0.350 - 0.415]	0.537 [0.486 - 0.586]
Age		30-34 yrs			>34 yrs	
Weight gain	Normal weight (n=12,302)	Underweight (n=2,460)	Overweight/Obese (n=4,749)	Normal weight (n=5,750)	Underweight (n=704)	Overweight/Obese (n=3,221)
-5	0.374 [0.281 - 0.478]	0.333 [0.298 - 0.371]	0.492 [0.456 - 0.528]	0.529 [0.348 - 0.703]	0.457 [0.383 - 0.533]	0.641 [0.586 - 0.691]
-4.5	0.364 [0.286 - 0.450]	0.334 [0.304 - 0.366]	0.493 [0.462 - 0.525]	0.509 [0.357 - 0.659]	0.450 [0.392 - 0.510]	0.633 [0.588 - 0.675]
-4	0.354 [0.291 - 0.423]	0.336 [0.311 - 0.361]	0.494 [0.467 - 0.522]	0.489 [0.363 - 0.616]	0.443 [0.398 - 0.489]	0.625 [0.588 - 0.660]
-3.5	0.345 [0.293 - 0.400]	0.337 [0.316 - 0.358]	0.496 [0.472 - 0.520]	0.469 [0.366 - 0.575]	0.437 [0.402 - 0.472]	0.618 [0.587 - 0.648]
-3	0.335 [0.293 - 0.380]	0.338 [0.321 - 0.356]	0.497 [0.476 - 0.518]	0.450 [0.365 - 0.537]	0.430 [0.402 - 0.459]	0.611 [0.584 - 0.637]
-2.5	0.327 [0.292 - 0.364]	0.340 [0.326 - 0.355]	0.499 [0.480 - 0.518]	0.431 [0.361 - 0.503]	0.424 [0.400 - 0.448]	0.605 [0.581 - 0.629]
-2	0.319 [0.290 - 0.350]	0.342 [0.330 - 0.355]	0.500 [0.483 - 0.518]	0.414 [0.355 - 0.475]	0.419 [0.398 - 0.441]	0.601 [0.578 - 0.623]
-1.5	0.312 [0.286 - 0.339]	0.345 [0.333 - 0.356]	0.502 [0.486 - 0.518]	0.399 [0.347 - 0.453]	0.416 [0.397 - 0.436]	0.597 [0.575 - 0.619]
-1	0.306 [0.282 - 0.331]	0.348 [0.337 - 0.359]	0.504 [0.489 - 0.520]	0.386 [0.339 - 0.436]	0.415 [0.397 - 0.435]	0.595 [0.573 - 0.616]
-0.5	0.302 [0.279 - 0.325]	0.351 [0.341 - 0.362]	0.506 [0.490 - 0.522]	0.376 [0.331 - 0.423]	0.416 [0.398 - 0.435]	0.593 [0.571 - 0.615]
Mean	0.299 [0.277 - 0.322]	0.355 [0.345 - 0.366]	0.508 [0.492 - 0.524]	0.369 [0.325 - 0.415]	0.419 [0.400 - 0.438]	0.593 [0.570 - 0.615]
0.5	0.298 [0.276 - 0.322]	0.360 [0.349 - 0.371]	0.510 [0.493 - 0.528]	0.364 [0.320 - 0.411]	0.422 [0.403 - 0.442]	0.593 [0.569 - 0.617]
1	0.300 [0.276 - 0.325]	0.365 [0.353 - 0.376]	0.513 [0.494 - 0.531]	0.362 [0.316 - 0.410]	0.427 [0.407 - 0.447]	0.595 [0.570 - 0.621]
1.5	0.303 [0.278 - 0.329]	0.370 [0.358 - 0.383]	0.515 [0.495 - 0.536]	0.362 [0.314 - 0.412]	0.434 [0.412 - 0.455]	0.599 [0.571 - 0.626]
2	0.307 [0.280 - 0.336]	0.377 [0.363 - 0.390]	0.518 [0.496 - 0.540]	0.363 [0.313 - 0.417]	0.443 [0.420 - 0.466]	0.602 [0.572 - 0.632]
2.5	0.312 [0.282 - 0.344]	0.384 [0.369 - 0.399]	0.521 [0.496 - 0.546]	0.367 [0.313 - 0.425]	0.455 [0.430 - 0.481]	0.607 [0.574 - 0.639]
3	0.317 [0.284 - 0.352]	0.392 [0.375 - 0.408]	0.524 [0.496 - 0.551]	0.373 [0.315 - 0.435]	0.471 [0.443 - 0.499]	0.612 [0.575 - 0.648]
3.5	0.323 [0.286 - 0.363]	0.400 [0.381 - 0.419]	0.527 [0.496 - 0.557]	0.381 [0.317 - 0.449]	0.489 [0.458 - 0.520]	0.618 [0.577 - 0.657]
4	0.330 [0.288 - 0.374]	0.408 [0.387 - 0.430]	0.530 [0.496 - 0.564]	0.390 [0.320 - 0.465]	0.508 [0.474 - 0.543]	0.623 [0.577 - 0.667]
4.5	0.337 [0.290 - 0.389]	0.417 [0.393 - 0.442]	0.533 [0.495 - 0.571]	0.400 [0.322 - 0.484]	0.527 [0.490 - 0.565]	0.629 [0.577 - 0.679]
5	0.347 [0.292 - 0.405]	0.427 [0.399 - 0.455]	0.537 [0.495 - 0.578]	0.412 [0.324 - 0.505]	0.545 [0.503 - 0.586]	0.636 [0.577 - 0.691]

2. Evaluation of weight gain between 24 and 34 weeks after pregnancy

- Generalized additive model (GAM) was used for predictive model of each disease.
- The above statistical model was applied to subsets of each age and BMI (ASIA).
- Reference weight gain = 5kg (Mean weight gain of mothers without any disease).

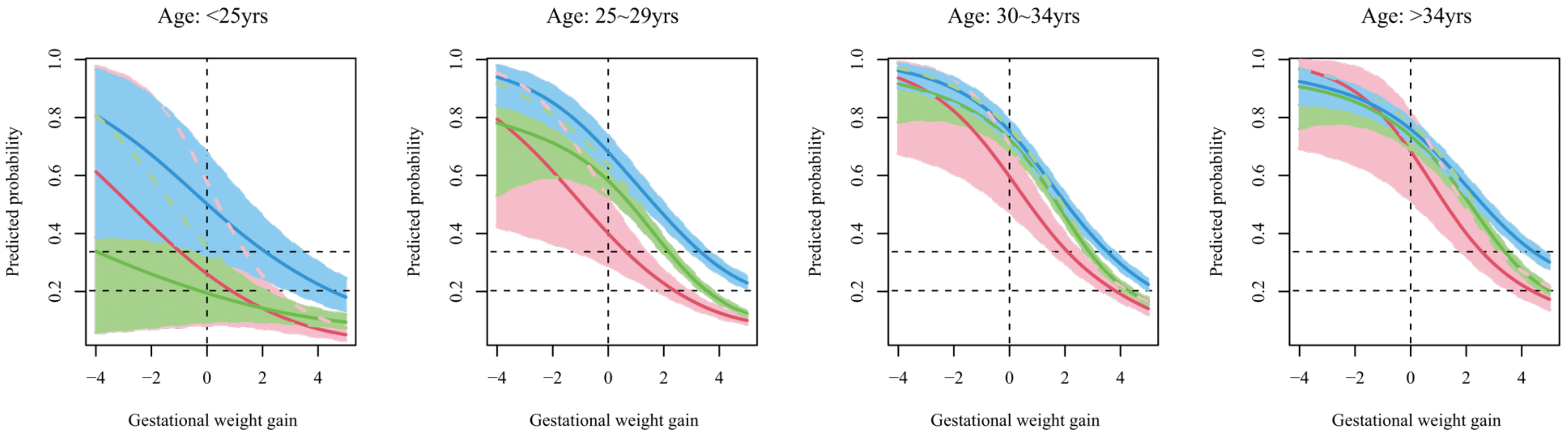

(a) **Gestational diabetes mellitus** (Red: Normal weight, Green: Underweight, Blue: Overweight/obese)

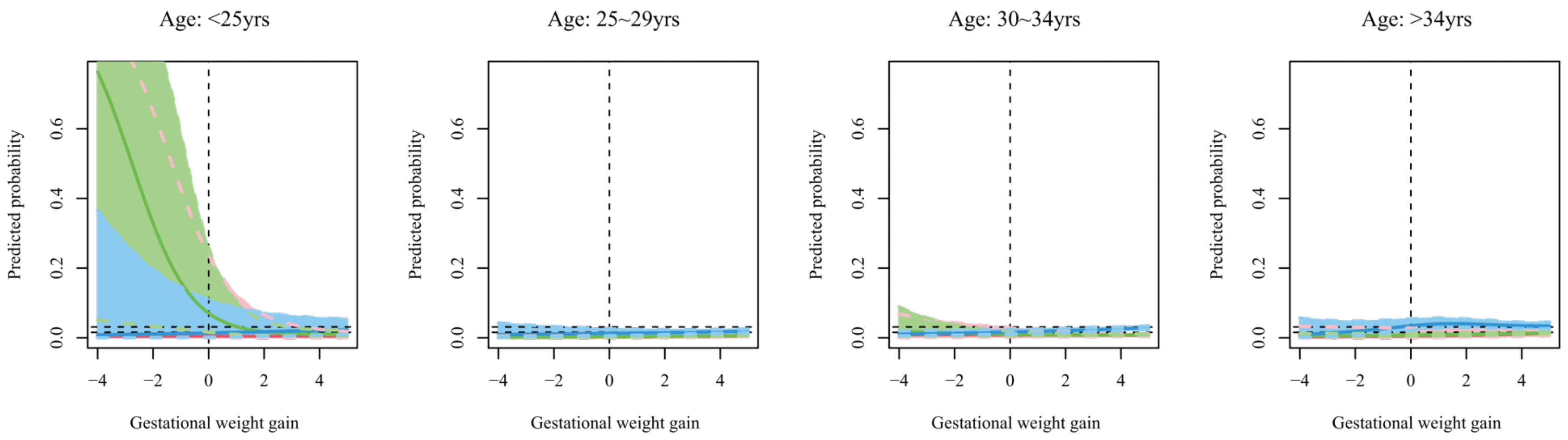

(b) **Preeclampsia** (Red: Normal weight, Green: Underweight, Blue: Overweight/obese)

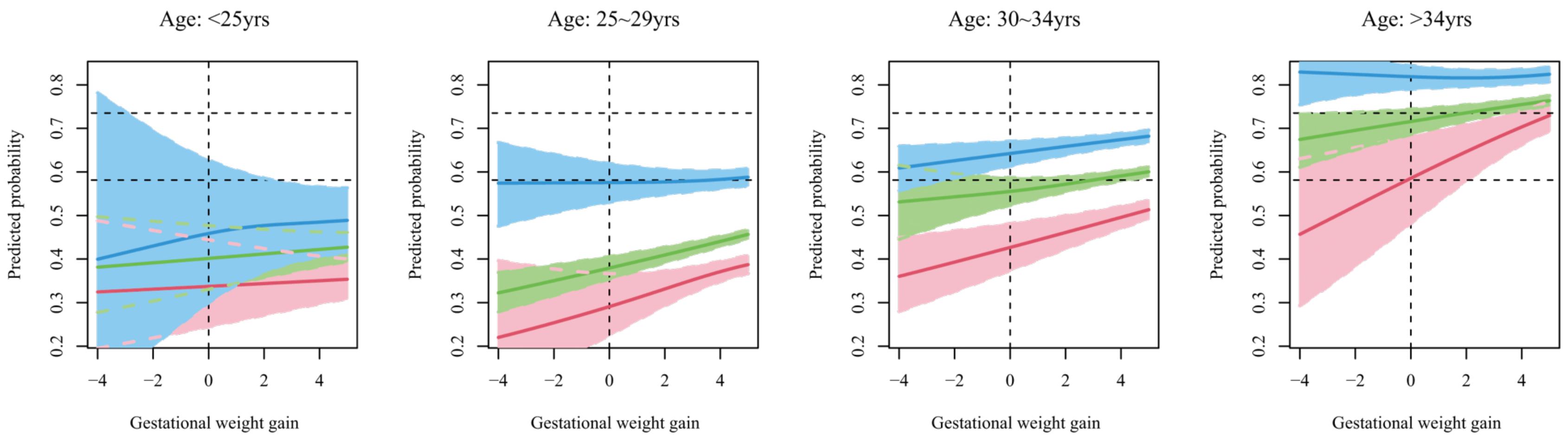

(c) **Cesarean delivery** (Red: Normal weight, Green: Underweight, Blue: Overweight/obese)

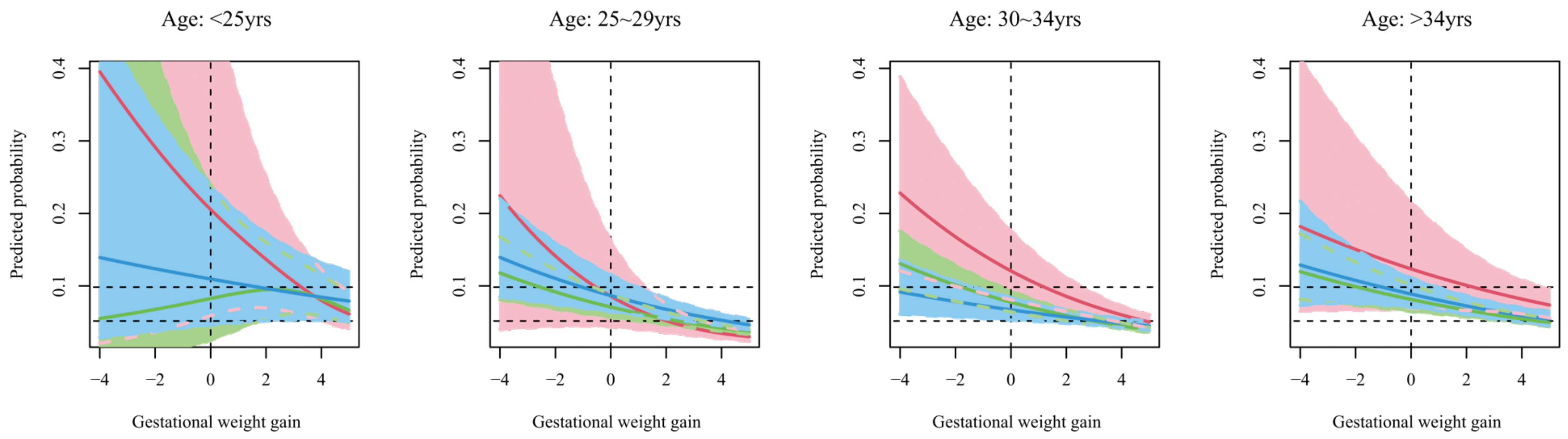

(d) **Preterm delivery** (Red: Normal weight, Green: Underweight, Blue: Overweight/obese)

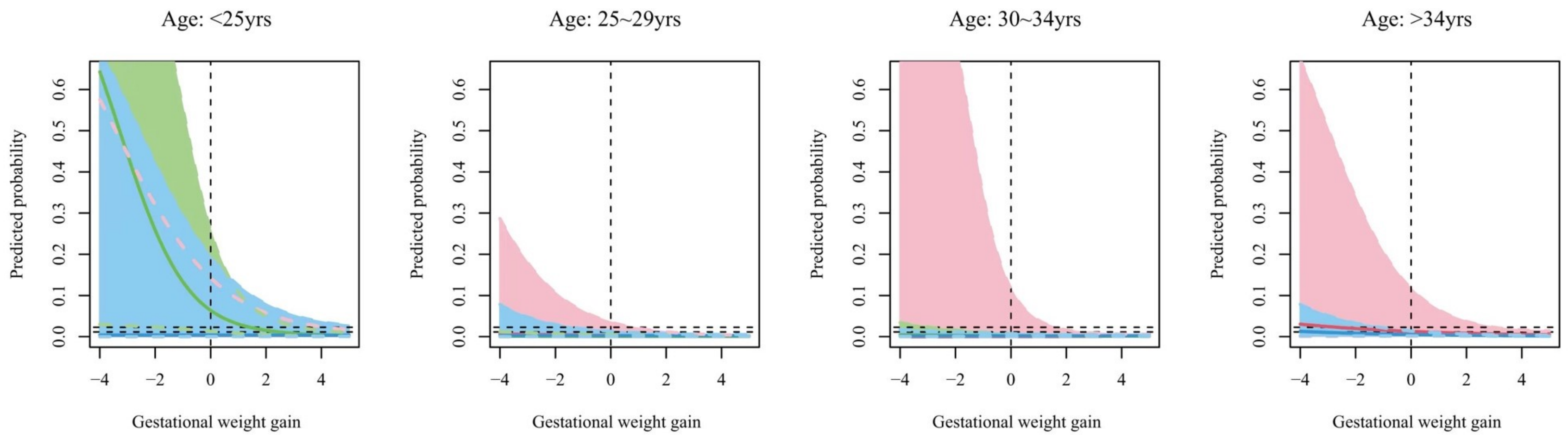

(e) **Stillbirth** (Red: Normal weight, Green: Underweight, Blue: Overweight/obese)

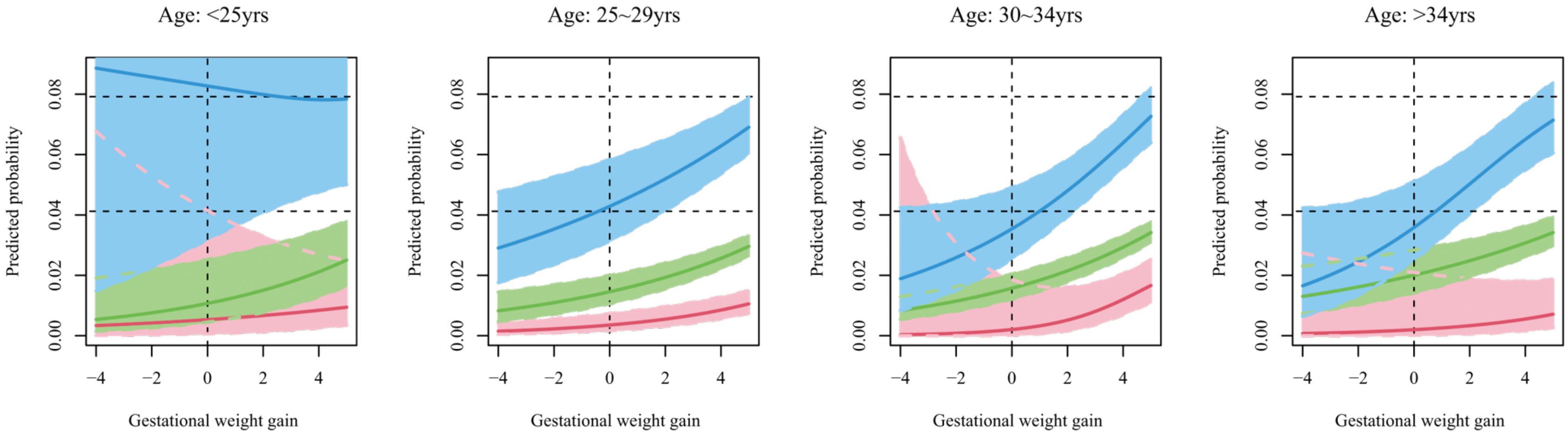

(f) **Macrosomia** (Red: Normal weight, Green: Underweight, Blue: Overweight/obese)

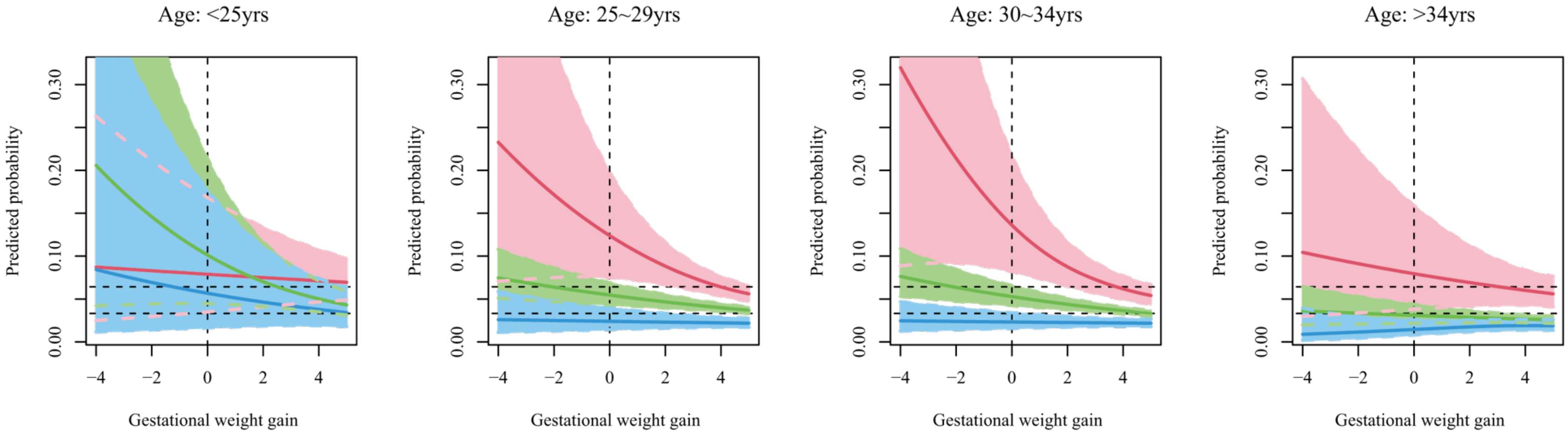

(g) **Small for gestational age** (Red: Normal weight, Green: Underweight, Blue: Overweight/obese)

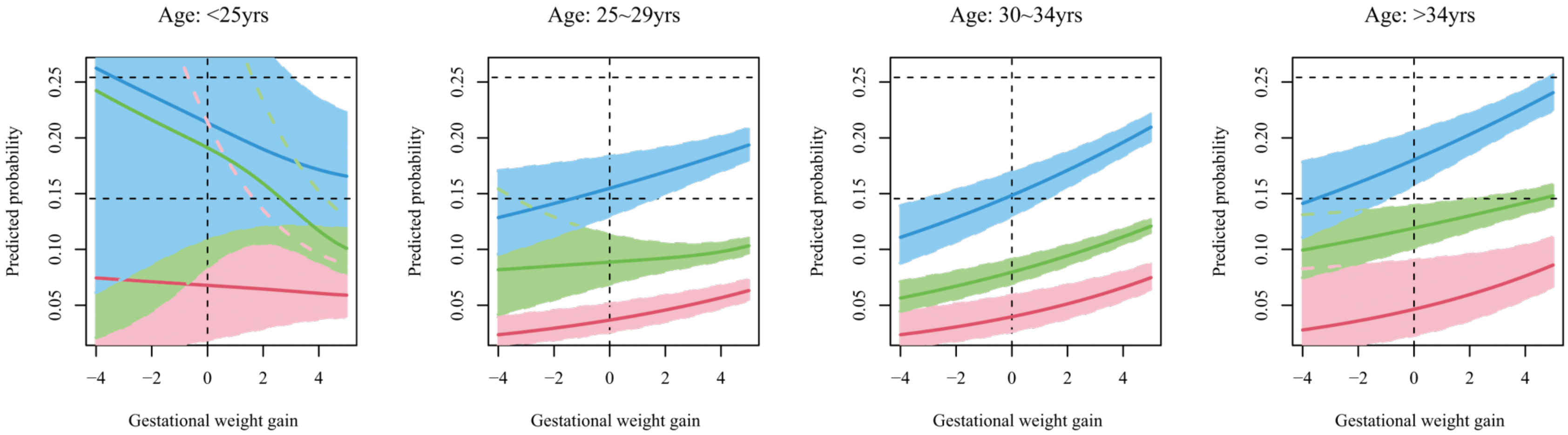

(h) **Large for gestational age** (Red: Normal weight, Green: Underweight, Blue: Overweight/obese)

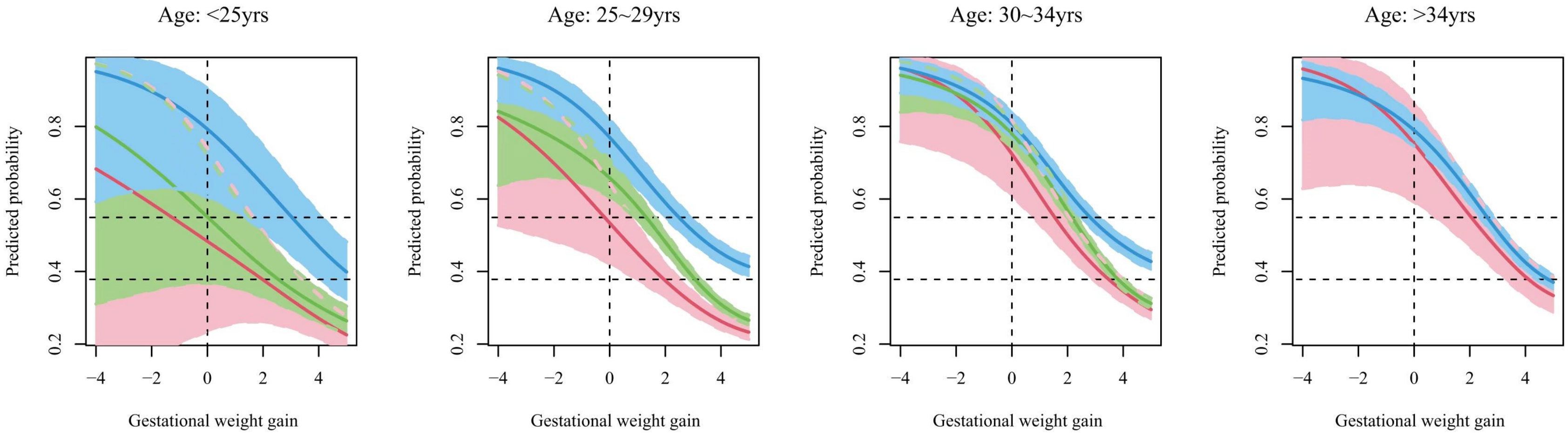

(i) **All disease** (Red: Normal weight, Green: Underweight, Blue: Overweight/obese)

2.1

Gestational diabetes mellitus

Age		<25 yrs			25-29 yrs	
Weight gain	Normal weight (n=1,324)	Underweight (n=698)	Overweight/Obese (n=314)	Normal weight (n=12,406)	Underweight (n=3,943)	Overweight/Obese (n=3,249)
-5	0.681 [0.059 - 0.986]	0.376 [0.053 - 0.867]	0.851 [0.391 - 0.981]	0.849 [0.461 - 0.974]	0.810 [0.485 - 0.951]	0.962 [0.867 - 0.990]
-4.5	0.640 [0.062 - 0.979]	0.354 [0.056 - 0.834]	0.827 [0.392 - 0.973]	0.819 [0.446 - 0.962]	0.797 [0.511 - 0.936]	0.952 [0.856 - 0.985]
-4	0.596 [0.065 - 0.969]	0.333 [0.060 - 0.795]	0.800 [0.392 - 0.961]	0.785 [0.431 - 0.946]	0.783 [0.536 - 0.919]	0.940 [0.844 - 0.978]
-3.5	0.551 [0.067 - 0.954]	0.313 [0.065 - 0.750]	0.771 [0.391 - 0.946]	0.747 [0.416 - 0.924]	0.768 [0.559 - 0.897]	0.924 [0.830 - 0.968]
-3	0.505 [0.070 - 0.933]	0.294 [0.069 - 0.699]	0.738 [0.388 - 0.926]	0.704 [0.400 - 0.894]	0.752 [0.577 - 0.871]	0.904 [0.813 - 0.954]
-2.5	0.460 [0.073 - 0.902]	0.275 [0.074 - 0.643]	0.703 [0.385 - 0.900]	0.657 [0.384 - 0.854]	0.734 [0.589 - 0.842]	0.880 [0.793 - 0.934]
-2	0.414 [0.076 - 0.859]	0.257 [0.078 - 0.584]	0.665 [0.378 - 0.867]	0.607 [0.368 - 0.804]	0.713 [0.594 - 0.809]	0.851 [0.768 - 0.908]
-1.5	0.370 [0.078 - 0.803]	0.240 [0.083 - 0.523]	0.625 [0.369 - 0.826]	0.554 [0.350 - 0.741]	0.689 [0.590 - 0.773]	0.817 [0.738 - 0.876]
-1	0.329 [0.080 - 0.732]	0.223 [0.087 - 0.463]	0.584 [0.357 - 0.780]	0.501 [0.331 - 0.670]	0.659 [0.577 - 0.733]	0.776 [0.703 - 0.836]
-0.5	0.289 [0.082 - 0.649]	0.208 [0.091 - 0.406]	0.541 [0.342 - 0.728]	0.447 [0.311 - 0.593]	0.624 [0.556 - 0.688]	0.731 [0.663 - 0.789]
Mean	0.253 [0.083 - 0.558]	0.193 [0.094 - 0.354]	0.499 [0.325 - 0.673]	0.396 [0.287 - 0.516]	0.582 [0.525 - 0.636]	0.681 [0.619 - 0.736]
0.5	0.220 [0.083 - 0.466]	0.179 [0.096 - 0.308]	0.457 [0.306 - 0.616]	0.348 [0.263 - 0.444]	0.533 [0.486 - 0.579]	0.627 [0.571 - 0.680]
1	0.190 [0.081 - 0.382]	0.166 [0.097 - 0.268]	0.417 [0.286 - 0.560]	0.303 [0.237 - 0.378]	0.477 [0.438 - 0.517]	0.573 [0.522 - 0.622]
1.5	0.163 [0.078 - 0.310]	0.153 [0.097 - 0.235]	0.378 [0.265 - 0.507]	0.263 [0.213 - 0.321]	0.418 [0.385 - 0.453]	0.518 [0.473 - 0.562]
2	0.139 [0.072 - 0.250]	0.142 [0.095 - 0.206]	0.343 [0.244 - 0.457]	0.227 [0.189 - 0.271]	0.359 [0.331 - 0.387]	0.464 [0.425 - 0.504]
2.5	0.118 [0.066 - 0.201]	0.131 [0.093 - 0.183]	0.310 [0.224 - 0.412]	0.196 [0.166 - 0.230]	0.302 [0.279 - 0.325]	0.413 [0.379 - 0.449]
3	0.100 [0.059 - 0.163]	0.122 [0.089 - 0.165]	0.279 [0.203 - 0.371]	0.169 [0.145 - 0.196]	0.250 [0.232 - 0.270]	0.365 [0.335 - 0.397]
3.5	0.084 [0.052 - 0.133]	0.114 [0.086 - 0.150]	0.251 [0.183 - 0.334]	0.146 [0.126 - 0.168]	0.207 [0.191 - 0.223]	0.322 [0.295 - 0.351]
4	0.071 [0.045 - 0.110]	0.107 [0.082 - 0.138]	0.225 [0.165 - 0.300]	0.127 [0.111 - 0.146]	0.172 [0.159 - 0.186]	0.285 [0.261 - 0.311]
4.5	0.060 [0.039 - 0.093]	0.100 [0.078 - 0.128]	0.202 [0.147 - 0.271]	0.112 [0.098 - 0.129]	0.145 [0.134 - 0.156]	0.254 [0.232 - 0.278]
5	0.052 [0.033 - 0.080]	0.095 [0.074 - 0.120]	0.182 [0.132 - 0.245]	0.101 [0.088 - 0.115]	0.124 [0.114 - 0.134]	0.230 [0.209 - 0.252]
Age		30-34 yrs			>34 yrs	
Weight gain	Normal weight (n=12,302)	Underweight (n=2,460)	Overweight/Obese (n=4,749)	Normal weight (n=5,750)	Underweight (n=704)	Overweight/Obese (n=3,221)
-5	0.963 [0.703 - 0.996]	0.937 [0.771 - 0.985]	0.976 [0.918 - 0.993]	0.983 [0.715 - 0.999]	0.924 [0.734 - 0.981]	0.942 [0.847 - 0.979]
-4.5	0.951 [0.691 - 0.994]	0.928 [0.780 - 0.979]	0.970 [0.912 - 0.990]	0.976 [0.705 - 0.999]	0.917 [0.754 - 0.975]	0.934 [0.847 - 0.973]
-4	0.935 [0.678 - 0.990]	0.917 [0.788 - 0.970]	0.961 [0.904 - 0.985]	0.967 [0.694 - 0.997]	0.908 [0.768 - 0.967]	0.925 [0.846 - 0.965]
-3.5	0.915 [0.664 - 0.983]	0.905 [0.792 - 0.959]	0.951 [0.894 - 0.978]	0.955 [0.682 - 0.995]	0.899 [0.778 - 0.958]	0.914 [0.843 - 0.955]
-3	0.890 [0.649 - 0.973]	0.891 [0.794 - 0.945]	0.937 [0.881 - 0.968]	0.939 [0.669 - 0.991]	0.887 [0.782 - 0.945]	0.902 [0.837 - 0.943]
-2.5	0.859 [0.630 - 0.956]	0.874 [0.791 - 0.928]	0.920 [0.866 - 0.954]	0.917 [0.655 - 0.985]	0.873 [0.780 - 0.930]	0.888 [0.829 - 0.928]
-2	0.821 [0.608 - 0.931]	0.855 [0.783 - 0.906]	0.899 [0.847 - 0.935]	0.888 [0.637 - 0.973]	0.855 [0.774 - 0.910]	0.870 [0.817 - 0.910]
-1.5	0.774 [0.582 - 0.895]	0.832 [0.769 - 0.880]	0.873 [0.823 - 0.911]	0.851 [0.615 - 0.953]	0.833 [0.763 - 0.886]	0.850 [0.801 - 0.888]
-1	0.721 [0.550 - 0.845]	0.803 [0.749 - 0.848]	0.841 [0.794 - 0.879]	0.804 [0.587 - 0.922]	0.806 [0.745 - 0.856]	0.825 [0.780 - 0.863]
-0.5	0.661 [0.513 - 0.782]	0.768 [0.721 - 0.810]	0.801 [0.757 - 0.839]	0.748 [0.552 - 0.877]	0.775 [0.720 - 0.821]	0.795 [0.753 - 0.831]
Mean	0.596 [0.473 - 0.707]	0.725 [0.684 - 0.762]	0.754 [0.712 - 0.791]	0.683 [0.512 - 0.815]	0.737 [0.688 - 0.780]	0.759 [0.720 - 0.794]
0.5	0.529 [0.430 - 0.625]	0.671 [0.635 - 0.706]	0.699 [0.660 - 0.735]	0.611 [0.467 - 0.739]	0.692 [0.649 - 0.732]	0.717 [0.680 - 0.752]
1	0.463 [0.385 - 0.543]	0.608 [0.575 - 0.640]	0.637 [0.602 - 0.671]	0.538 [0.418 - 0.653]	0.641 [0.603 - 0.678]	0.670 [0.634 - 0.704]
1.5	0.402 [0.340 - 0.467]	0.536 [0.507 - 0.566]	0.573 [0.540 - 0.605]	0.466 [0.369 - 0.567]	0.583 [0.549 - 0.617]	0.619 [0.585 - 0.652]
2	0.347 [0.297 - 0.400]	0.461 [0.435 - 0.486]	0.508 [0.478 - 0.538]	0.401 [0.321 - 0.486]	0.519 [0.488 - 0.550]	0.565 [0.533 - 0.597]
2.5	0.299 [0.258 - 0.343]	0.387 [0.366 - 0.409]	0.447 [0.419 - 0.475]	0.344 [0.278 - 0.416]	0.451 [0.423 - 0.479]	0.511 [0.480 - 0.542]
3	0.257 [0.224 - 0.295]	0.321 [0.303 - 0.340]	0.390 [0.366 - 0.416]	0.296 [0.240 - 0.358]	0.385 [0.360 - 0.410]	0.459 [0.429 - 0.489]
3.5	0.222 [0.193 - 0.254]	0.267 [0.251 - 0.283]	0.340 [0.317 - 0.363]	0.256 [0.208 - 0.310]	0.325 [0.302 - 0.348]	0.411 [0.383 - 0.439]
4	0.191 [0.167 - 0.219]	0.224 [0.211 - 0.238]	0.295 [0.274 - 0.318]	0.223 [0.181 - 0.272]	0.274 [0.255 - 0.295]	0.369 [0.342 - 0.396]
4.5	0.165 [0.143 - 0.189]	0.191 [0.179 - 0.203]	0.257 [0.237 - 0.278]	0.196 [0.158 - 0.241]	0.235 [0.217 - 0.254]	0.332 [0.307 - 0.359]
5	0.141 [0.122 - 0.163]	0.165 [0.154 - 0.177]	0.225 [0.206 - 0.244]	0.173 [0.138 - 0.215]	0.204 [0.188 - 0.222]	0.302 [0.277 - 0.328]

2.2 Preeclampsia

Age		<25 yrs			25-29 yrs	
Weight gain	Normal weight (n=1,324)	Underweight (n=698)	Overweight/Obese (n=314)	Normal weight (n=12,406)	Underweight (n=3,943)	Overweight/Obese (n=3,249)
-5	0.001 [0.000 - 0.429]	0.842 [0.043 - 0.998]	0.005 [0.000 - 0.183]	0.001 [0.000 - 0.005]	0.001 [0.000 - 0.003]	0.008 [0.001 - 0.040]
-4.5	0.001 [0.000 - 0.372]	0.775 [0.041 - 0.996]	0.006 [0.000 - 0.166]	0.001 [0.000 - 0.005]	0.001 [0.001 - 0.003]	0.008 [0.002 - 0.037]
-4	0.001 [0.000 - 0.319]	0.691 [0.038 - 0.992]	0.006 [0.000 - 0.150]	0.001 [0.000 - 0.005]	0.001 [0.001 - 0.003]	0.008 [0.002 - 0.035]
-3.5	0.001 [0.000 - 0.270]	0.592 [0.035 - 0.983]	0.007 [0.000 - 0.136]	0.001 [0.000 - 0.005]	0.002 [0.001 - 0.004]	0.009 [0.002 - 0.033]
-3	0.002 [0.000 - 0.227]	0.485 [0.033 - 0.963]	0.007 [0.000 - 0.123]	0.001 [0.000 - 0.005]	0.002 [0.001 - 0.004]	0.009 [0.003 - 0.031]
-2.5	0.002 [0.000 - 0.189]	0.379 [0.030 - 0.923]	0.008 [0.000 - 0.112]	0.001 [0.000 - 0.005]	0.002 [0.001 - 0.004]	0.010 [0.003 - 0.030]
-2	0.002 [0.000 - 0.157]	0.283 [0.027 - 0.847]	0.008 [0.001 - 0.102]	0.001 [0.000 - 0.005]	0.002 [0.001 - 0.004]	0.010 [0.004 - 0.028]
-1.5	0.002 [0.000 - 0.129]	0.204 [0.025 - 0.723]	0.009 [0.001 - 0.093]	0.001 [0.000 - 0.005]	0.002 [0.001 - 0.004]	0.011 [0.004 - 0.027]
-1	0.002 [0.000 - 0.107]	0.143 [0.022 - 0.558]	0.010 [0.001 - 0.086]	0.002 [0.000 - 0.006]	0.002 [0.001 - 0.005]	0.011 [0.005 - 0.026]
-0.5	0.002 [0.000 - 0.088]	0.098 [0.018 - 0.387]	0.010 [0.001 - 0.079]	0.002 [0.001 - 0.006]	0.003 [0.001 - 0.005]	0.012 [0.005 - 0.025]
Mean	0.002 [0.000 - 0.073]	0.067 [0.015 - 0.251]	0.011 [0.002 - 0.074]	0.002 [0.001 - 0.006]	0.003 [0.002 - 0.005]	0.012 [0.006 - 0.024]
0.5	0.002 [0.000 - 0.060]	0.046 [0.012 - 0.160]	0.012 [0.002 - 0.069]	0.002 [0.001 - 0.006]	0.003 [0.002 - 0.005]	0.013 [0.007 - 0.024]
1	0.003 [0.000 - 0.050]	0.032 [0.010 - 0.104]	0.013 [0.002 - 0.065]	0.002 [0.001 - 0.006]	0.003 [0.002 - 0.006]	0.013 [0.007 - 0.023]
1.5	0.003 [0.000 - 0.042]	0.024 [0.008 - 0.070]	0.014 [0.003 - 0.061]	0.003 [0.001 - 0.006]	0.004 [0.002 - 0.006]	0.014 [0.008 - 0.023]
2	0.003 [0.000 - 0.036]	0.018 [0.006 - 0.050]	0.015 [0.004 - 0.059]	0.003 [0.001 - 0.007]	0.004 [0.003 - 0.006]	0.014 [0.009 - 0.023]
2.5	0.003 [0.000 - 0.031]	0.015 [0.006 - 0.038]	0.016 [0.004 - 0.056]	0.003 [0.001 - 0.007]	0.004 [0.003 - 0.006]	0.015 [0.010 - 0.023]
3	0.003 [0.000 - 0.027]	0.013 [0.005 - 0.030]	0.017 [0.005 - 0.055]	0.003 [0.002 - 0.007]	0.005 [0.003 - 0.007]	0.016 [0.011 - 0.023]
3.5	0.003 [0.000 - 0.024]	0.011 [0.005 - 0.026]	0.018 [0.006 - 0.053]	0.004 [0.002 - 0.007]	0.005 [0.004 - 0.007]	0.017 [0.012 - 0.023]
4	0.004 [0.001 - 0.021]	0.011 [0.005 - 0.023]	0.019 [0.007 - 0.052]	0.004 [0.002 - 0.007]	0.006 [0.004 - 0.008]	0.017 [0.013 - 0.024]
4.5	0.004 [0.001 - 0.019]	0.011 [0.005 - 0.022]	0.021 [0.008 - 0.052]	0.004 [0.002 - 0.008]	0.006 [0.005 - 0.008]	0.018 [0.014 - 0.025]
5	0.004 [0.001 - 0.018]	0.011 [0.005 - 0.022]	0.022 [0.009 - 0.052]	0.005 [0.003 - 0.008]	0.007 [0.005 - 0.008]	0.019 [0.015 - 0.026]
Age		30-34 yrs			>34 yrs	
Weight gain	Normal weight (n=12,302)	Underweight (n=2,460)	Overweight/Obese (n=4,749)	Normal weight (n=5,750)	Underweight (n=704)	Overweight/Obese (n=3,221)
-5	0.009 [0.001 - 0.084]	0.019 [0.003 - 0.113]	0.009 [0.005 - 0.019]	0.001 [0.000 - 0.159]	0.004 [0.002 - 0.010]	0.005 [0.000 - 0.069]
-4.5	0.009 [0.001 - 0.075]	0.018 [0.003 - 0.092]	0.010 [0.005 - 0.019]	0.001 [0.000 - 0.136]	0.004 [0.002 - 0.011]	0.007 [0.001 - 0.062]
-4	0.009 [0.001 - 0.068]	0.016 [0.003 - 0.075]	0.011 [0.006 - 0.020]	0.001 [0.000 - 0.116]	0.005 [0.002 - 0.011]	0.008 [0.001 - 0.056]
-3.5	0.009 [0.001 - 0.061]	0.015 [0.004 - 0.061]	0.011 [0.006 - 0.020]	0.002 [0.000 - 0.099]	0.005 [0.002 - 0.011]	0.010 [0.002 - 0.052]
-3	0.009 [0.001 - 0.054]	0.014 [0.004 - 0.050]	0.012 [0.007 - 0.021]	0.002 [0.000 - 0.084]	0.005 [0.002 - 0.011]	0.012 [0.003 - 0.049]
-2.5	0.009 [0.002 - 0.049]	0.013 [0.004 - 0.041]	0.012 [0.007 - 0.021]	0.002 [0.000 - 0.072]	0.005 [0.003 - 0.012]	0.014 [0.004 - 0.047]
-2	0.009 [0.002 - 0.043]	0.013 [0.005 - 0.034]	0.013 [0.008 - 0.022]	0.002 [0.000 - 0.062]	0.006 [0.003 - 0.012]	0.018 [0.006 - 0.047]
-1.5	0.008 [0.002 - 0.039]	0.012 [0.005 - 0.029]	0.014 [0.009 - 0.022]	0.003 [0.000 - 0.054]	0.006 [0.003 - 0.012]	0.021 [0.009 - 0.048]
-1	0.008 [0.002 - 0.035]	0.011 [0.005 - 0.024]	0.015 [0.009 - 0.023]	0.003 [0.000 - 0.047]	0.007 [0.004 - 0.012]	0.025 [0.013 - 0.050]
-0.5	0.008 [0.002 - 0.031]	0.010 [0.005 - 0.021]	0.016 [0.010 - 0.023]	0.003 [0.000 - 0.042]	0.007 [0.004 - 0.013]	0.030 [0.017 - 0.052]
Mean	0.008 [0.002 - 0.028]	0.010 [0.005 - 0.018]	0.016 [0.011 - 0.024]	0.004 [0.000 - 0.037]	0.007 [0.004 - 0.013]	0.034 [0.021 - 0.055]
0.5	0.008 [0.002 - 0.025]	0.009 [0.005 - 0.016]	0.017 [0.012 - 0.025]	0.004 [0.001 - 0.034]	0.008 [0.005 - 0.013]	0.038 [0.025 - 0.057]
1	0.008 [0.003 - 0.023]	0.009 [0.006 - 0.014]	0.018 [0.013 - 0.025]	0.005 [0.001 - 0.031]	0.009 [0.005 - 0.014]	0.041 [0.028 - 0.058]
1.5	0.008 [0.003 - 0.020]	0.009 [0.006 - 0.013]	0.019 [0.014 - 0.026]	0.006 [0.001 - 0.029]	0.009 [0.006 - 0.014]	0.041 [0.030 - 0.057]
2	0.008 [0.003 - 0.018]	0.008 [0.006 - 0.012]	0.020 [0.016 - 0.027]	0.006 [0.001 - 0.027]	0.010 [0.007 - 0.014]	0.041 [0.030 - 0.055]
2.5	0.007 [0.003 - 0.017]	0.008 [0.006 - 0.012]	0.022 [0.017 - 0.028]	0.007 [0.002 - 0.026]	0.010 [0.007 - 0.015]	0.039 [0.029 - 0.052]
3	0.007 [0.004 - 0.015]	0.008 [0.006 - 0.011]	0.023 [0.018 - 0.029]	0.008 [0.003 - 0.025]	0.011 [0.008 - 0.015]	0.037 [0.028 - 0.049]
3.5	0.007 [0.004 - 0.014]	0.008 [0.006 - 0.011]	0.024 [0.020 - 0.030]	0.009 [0.003 - 0.025]	0.012 [0.009 - 0.016]	0.035 [0.027 - 0.047]
4	0.007 [0.004 - 0.013]	0.009 [0.007 - 0.011]	0.025 [0.021 - 0.031]	0.010 [0.004 - 0.025]	0.012 [0.009 - 0.016]	0.034 [0.026 - 0.045]
4.5	0.007 [0.004 - 0.012]	0.009 [0.007 - 0.011]	0.027 [0.022 - 0.032]	0.012 [0.005 - 0.026]	0.013 [0.010 - 0.017]	0.034 [0.025 - 0.045]
5	0.007 [0.004 - 0.011]	0.009 [0.007 - 0.012]	0.028 [0.024 - 0.034]	0.013 [0.006 - 0.027]	0.014 [0.011 - 0.018]	0.034 [0.025 - 0.045]

2.3

Cesarean delivery

Age		<25 yrs			25-29 yrs	
Weight gain	Normal weight (n=1,324)	Underweight (n=698)	Overweight/Obese (n=314)	Normal weight (n=12,406)	Underweight (n=3,943)	Overweight/Obese (n=3,249)
-5	0.321 [0.184 - 0.499]	0.375 [0.267 - 0.497]	0.362 [0.057 - 0.841]	0.200 [0.080 - 0.416]	0.304 [0.267 - 0.343]	0.574 [0.459 - 0.682]
-4.5	0.323 [0.190 - 0.493]	0.378 [0.273 - 0.495]	0.371 [0.071 - 0.821]	0.207 [0.090 - 0.410]	0.311 [0.275 - 0.348]	0.574 [0.467 - 0.675]
-4	0.325 [0.195 - 0.487]	0.380 [0.279 - 0.493]	0.381 [0.087 - 0.799]	0.216 [0.100 - 0.403]	0.318 [0.284 - 0.354]	0.574 [0.476 - 0.667]
-3.5	0.326 [0.201 - 0.482]	0.383 [0.286 - 0.490]	0.391 [0.107 - 0.776]	0.224 [0.112 - 0.397]	0.325 [0.292 - 0.359]	0.574 [0.484 - 0.660]
-3	0.328 [0.207 - 0.476]	0.385 [0.292 - 0.488]	0.401 [0.129 - 0.752]	0.232 [0.125 - 0.391]	0.332 [0.301 - 0.365]	0.575 [0.492 - 0.653]
-2.5	0.329 [0.213 - 0.471]	0.388 [0.298 - 0.486]	0.411 [0.154 - 0.728]	0.241 [0.138 - 0.386]	0.339 [0.309 - 0.371]	0.575 [0.500 - 0.647]
-2	0.331 [0.219 - 0.465]	0.391 [0.305 - 0.484]	0.421 [0.182 - 0.704]	0.250 [0.153 - 0.381]	0.347 [0.318 - 0.377]	0.575 [0.507 - 0.640]
-1.5	0.332 [0.226 - 0.460]	0.393 [0.311 - 0.482]	0.431 [0.211 - 0.683]	0.259 [0.169 - 0.376]	0.354 [0.327 - 0.383]	0.575 [0.513 - 0.635]
-1	0.334 [0.232 - 0.455]	0.396 [0.318 - 0.479]	0.441 [0.239 - 0.664]	0.269 [0.186 - 0.372]	0.362 [0.336 - 0.388]	0.575 [0.520 - 0.629]
-0.5	0.336 [0.238 - 0.449]	0.398 [0.324 - 0.477]	0.450 [0.267 - 0.648]	0.279 [0.203 - 0.369]	0.369 [0.345 - 0.395]	0.575 [0.525 - 0.624]
Mean	0.337 [0.245 - 0.444]	0.401 [0.331 - 0.475]	0.459 [0.294 - 0.633]	0.288 [0.221 - 0.367]	0.377 [0.354 - 0.401]	0.576 [0.531 - 0.619]
0.5	0.339 [0.251 - 0.439]	0.404 [0.338 - 0.473]	0.467 [0.318 - 0.621]	0.299 [0.238 - 0.367]	0.385 [0.363 - 0.407]	0.576 [0.536 - 0.615]
1	0.340 [0.258 - 0.434]	0.406 [0.344 - 0.471]	0.473 [0.339 - 0.611]	0.309 [0.256 - 0.367]	0.393 [0.373 - 0.413]	0.576 [0.540 - 0.611]
1.5	0.342 [0.265 - 0.429]	0.409 [0.351 - 0.469]	0.478 [0.357 - 0.601]	0.320 [0.274 - 0.369]	0.401 [0.382 - 0.419]	0.577 [0.544 - 0.608]
2	0.344 [0.271 - 0.424]	0.411 [0.357 - 0.468]	0.481 [0.371 - 0.593]	0.331 [0.291 - 0.373]	0.408 [0.391 - 0.426]	0.577 [0.548 - 0.606]
2.5	0.345 [0.278 - 0.419]	0.414 [0.364 - 0.466]	0.483 [0.382 - 0.586]	0.341 [0.308 - 0.377]	0.416 [0.401 - 0.432]	0.578 [0.552 - 0.604]
3	0.347 [0.285 - 0.415]	0.417 [0.370 - 0.465]	0.485 [0.391 - 0.580]	0.352 [0.323 - 0.383]	0.424 [0.410 - 0.439]	0.579 [0.555 - 0.603]
3.5	0.349 [0.291 - 0.411]	0.419 [0.377 - 0.463]	0.486 [0.398 - 0.575]	0.362 [0.337 - 0.389]	0.433 [0.420 - 0.446]	0.581 [0.559 - 0.603]
4	0.350 [0.298 - 0.407]	0.422 [0.383 - 0.462]	0.488 [0.405 - 0.572]	0.372 [0.349 - 0.396]	0.441 [0.429 - 0.452]	0.583 [0.562 - 0.604]
4.5	0.352 [0.304 - 0.403]	0.425 [0.389 - 0.461]	0.490 [0.410 - 0.570]	0.380 [0.359 - 0.402]	0.449 [0.438 - 0.460]	0.585 [0.565 - 0.605]
5	0.354 [0.309 - 0.400]	0.427 [0.394 - 0.461]	0.492 [0.415 - 0.569]	0.388 [0.368 - 0.409]	0.457 [0.447 - 0.467]	0.588 [0.568 - 0.607]
Age		30-34 yrs			>34 yrs	
Weight gain	Normal weight (n=12,302)	Underweight (n=2,460)	Overweight/Obese (n=4,749)	Normal weight (n=5,750)	Underweight (n=704)	Overweight/Obese (n=3,221)
-5	0.342 [0.263 - 0.432]	0.528 [0.420 - 0.634]	0.601 [0.545 - 0.654]	0.425 [0.253 - 0.618]	0.666 [0.606 - 0.722]	0.834 [0.738 - 0.899]
-4.5	0.350 [0.273 - 0.437]	0.531 [0.432 - 0.627]	0.605 [0.552 - 0.656]	0.441 [0.272 - 0.624]	0.672 [0.615 - 0.724]	0.832 [0.746 - 0.893]
-4	0.359 [0.283 - 0.441]	0.533 [0.444 - 0.621]	0.609 [0.559 - 0.657]	0.457 [0.293 - 0.630]	0.677 [0.623 - 0.726]	0.831 [0.754 - 0.887]
-3.5	0.367 [0.294 - 0.446]	0.536 [0.456 - 0.615]	0.614 [0.566 - 0.659]	0.473 [0.315 - 0.637]	0.682 [0.632 - 0.728]	0.829 [0.761 - 0.881]
-3	0.375 [0.305 - 0.450]	0.539 [0.467 - 0.609]	0.618 [0.573 - 0.660]	0.489 [0.337 - 0.643]	0.687 [0.640 - 0.730]	0.828 [0.768 - 0.875]
-2.5	0.383 [0.316 - 0.455]	0.541 [0.478 - 0.604]	0.622 [0.580 - 0.662]	0.505 [0.360 - 0.650]	0.692 [0.649 - 0.733]	0.826 [0.773 - 0.869]
-2	0.392 [0.328 - 0.460]	0.544 [0.488 - 0.599]	0.626 [0.587 - 0.664]	0.521 [0.384 - 0.656]	0.697 [0.657 - 0.735]	0.825 [0.778 - 0.864]
-1.5	0.400 [0.339 - 0.464]	0.547 [0.497 - 0.595]	0.630 [0.594 - 0.665]	0.538 [0.408 - 0.662]	0.702 [0.665 - 0.737]	0.823 [0.782 - 0.858]
-1	0.409 [0.351 - 0.469]	0.550 [0.506 - 0.592]	0.634 [0.601 - 0.667]	0.554 [0.432 - 0.669]	0.707 [0.673 - 0.739]	0.822 [0.785 - 0.853]
-0.5	0.417 [0.363 - 0.474]	0.552 [0.515 - 0.589]	0.638 [0.607 - 0.668]	0.570 [0.457 - 0.675]	0.712 [0.681 - 0.742]	0.820 [0.788 - 0.849]
Mean	0.426 [0.375 - 0.479]	0.556 [0.523 - 0.587]	0.642 [0.614 - 0.670]	0.585 [0.482 - 0.682]	0.717 [0.688 - 0.744]	0.819 [0.790 - 0.845]
0.5	0.434 [0.387 - 0.483]	0.559 [0.531 - 0.587]	0.647 [0.620 - 0.672]	0.601 [0.507 - 0.688]	0.722 [0.696 - 0.747]	0.818 [0.792 - 0.841]
1	0.443 [0.399 - 0.488]	0.563 [0.539 - 0.587]	0.651 [0.627 - 0.674]	0.616 [0.532 - 0.695]	0.727 [0.703 - 0.749]	0.817 [0.793 - 0.838]
1.5	0.452 [0.411 - 0.493]	0.567 [0.547 - 0.588]	0.655 [0.633 - 0.676]	0.632 [0.556 - 0.702]	0.731 [0.710 - 0.752]	0.816 [0.794 - 0.836]
2	0.461 [0.423 - 0.499]	0.572 [0.554 - 0.590]	0.659 [0.639 - 0.678]	0.647 [0.579 - 0.709]	0.736 [0.717 - 0.754]	0.816 [0.796 - 0.834]
2.5	0.469 [0.435 - 0.504]	0.577 [0.561 - 0.593]	0.663 [0.645 - 0.680]	0.661 [0.602 - 0.716]	0.741 [0.724 - 0.757]	0.816 [0.797 - 0.834]
3	0.478 [0.447 - 0.509]	0.582 [0.567 - 0.596]	0.666 [0.650 - 0.682]	0.676 [0.624 - 0.723]	0.745 [0.730 - 0.760]	0.816 [0.798 - 0.833]
3.5	0.487 [0.459 - 0.515]	0.586 [0.573 - 0.599]	0.670 [0.655 - 0.685]	0.690 [0.644 - 0.732]	0.750 [0.736 - 0.763]	0.818 [0.800 - 0.834]
4	0.496 [0.471 - 0.521]	0.591 [0.579 - 0.603]	0.674 [0.660 - 0.688]	0.703 [0.663 - 0.741]	0.754 [0.742 - 0.766]	0.819 [0.802 - 0.835]
4.5	0.505 [0.482 - 0.528]	0.596 [0.584 - 0.607]	0.678 [0.664 - 0.692]	0.717 [0.679 - 0.751]	0.759 [0.747 - 0.770]	0.821 [0.804 - 0.838]
5	0.513 [0.492 - 0.535]	0.600 [0.589 - 0.611]	0.682 [0.668 - 0.696]	0.730 [0.694 - 0.762]	0.763 [0.752 - 0.774]	0.824 [0.806 - 0.840]

2.4

Preterm delivery

Age		<25 yrs			25-29 yrs	
Weight gain	Normal weight (n=1,324)	Underweight (n=698)	Overweight/Obese (n=314)	Normal weight (n=12,406)	Underweight (n=3,943)	Overweight/Obese (n=3,249)
-5	0.455 [0.018 - 0.974]	0.046 [0.001 - 0.768]	0.130 [0.031 - 0.409]	0.265 [0.038 - 0.766]	0.132 [0.090 - 0.190]	0.156 [0.092 - 0.251]
-4.5	0.426 [0.021 - 0.963]	0.049 [0.001 - 0.720]	0.127 [0.032 - 0.387]	0.239 [0.039 - 0.708]	0.124 [0.086 - 0.177]	0.147 [0.089 - 0.234]
-4	0.397 [0.024 - 0.947]	0.052 [0.001 - 0.666]	0.124 [0.034 - 0.366]	0.215 [0.040 - 0.642]	0.117 [0.082 - 0.164]	0.139 [0.086 - 0.217]
-3.5	0.369 [0.027 - 0.925]	0.055 [0.002 - 0.608]	0.121 [0.035 - 0.344]	0.193 [0.041 - 0.571]	0.110 [0.078 - 0.152]	0.131 [0.083 - 0.201]
-3	0.342 [0.031 - 0.895]	0.058 [0.003 - 0.547]	0.119 [0.036 - 0.324]	0.172 [0.042 - 0.498]	0.103 [0.075 - 0.141]	0.124 [0.080 - 0.186]
-2.5	0.316 [0.035 - 0.856]	0.062 [0.005 - 0.485]	0.116 [0.038 - 0.304]	0.154 [0.043 - 0.425]	0.097 [0.072 - 0.131]	0.117 [0.077 - 0.172]
-2	0.291 [0.039 - 0.805]	0.065 [0.007 - 0.425]	0.113 [0.039 - 0.286]	0.137 [0.043 - 0.356]	0.091 [0.068 - 0.121]	0.110 [0.075 - 0.159]
-1.5	0.267 [0.044 - 0.743]	0.069 [0.009 - 0.369]	0.111 [0.041 - 0.268]	0.121 [0.044 - 0.294]	0.086 [0.065 - 0.112]	0.104 [0.072 - 0.147]
-1	0.245 [0.049 - 0.670]	0.073 [0.013 - 0.318]	0.108 [0.042 - 0.251]	0.108 [0.044 - 0.240]	0.080 [0.062 - 0.103]	0.098 [0.069 - 0.136]
-0.5	0.224 [0.055 - 0.590]	0.077 [0.018 - 0.275]	0.106 [0.043 - 0.234]	0.095 [0.044 - 0.195]	0.075 [0.059 - 0.095]	0.092 [0.067 - 0.125]
Mean	0.204 [0.060 - 0.507]	0.081 [0.024 - 0.239]	0.103 [0.045 - 0.219]	0.084 [0.043 - 0.159]	0.071 [0.057 - 0.088]	0.087 [0.065 - 0.115]
0.5	0.185 [0.065 - 0.428]	0.085 [0.031 - 0.212]	0.101 [0.046 - 0.205]	0.074 [0.041 - 0.130]	0.066 [0.054 - 0.081]	0.081 [0.062 - 0.106]
1	0.168 [0.068 - 0.357]	0.089 [0.039 - 0.191]	0.098 [0.048 - 0.192]	0.066 [0.040 - 0.107]	0.062 [0.051 - 0.075]	0.077 [0.060 - 0.098]
1.5	0.151 [0.070 - 0.297]	0.093 [0.047 - 0.174]	0.096 [0.049 - 0.179]	0.058 [0.038 - 0.089]	0.058 [0.049 - 0.069]	0.072 [0.057 - 0.090]
2	0.135 [0.069 - 0.247]	0.095 [0.054 - 0.160]	0.094 [0.050 - 0.168]	0.052 [0.036 - 0.075]	0.055 [0.047 - 0.064]	0.068 [0.055 - 0.083]
2.5	0.120 [0.067 - 0.205]	0.095 [0.060 - 0.149]	0.091 [0.051 - 0.158]	0.046 [0.033 - 0.064]	0.051 [0.044 - 0.059]	0.064 [0.052 - 0.077]
3	0.105 [0.063 - 0.171]	0.093 [0.062 - 0.138]	0.089 [0.052 - 0.148]	0.042 [0.031 - 0.056]	0.048 [0.042 - 0.055]	0.060 [0.050 - 0.071]
3.5	0.092 [0.057 - 0.144]	0.089 [0.062 - 0.127]	0.087 [0.053 - 0.140]	0.038 [0.029 - 0.049]	0.045 [0.040 - 0.050]	0.056 [0.047 - 0.066]
4	0.080 [0.051 - 0.122]	0.083 [0.059 - 0.116]	0.085 [0.054 - 0.133]	0.035 [0.027 - 0.044]	0.042 [0.038 - 0.047]	0.053 [0.045 - 0.062]
4.5	0.070 [0.046 - 0.105]	0.075 [0.054 - 0.103]	0.083 [0.054 - 0.126]	0.032 [0.025 - 0.041]	0.039 [0.035 - 0.043]	0.050 [0.042 - 0.058]
5	0.062 [0.041 - 0.092]	0.067 [0.048 - 0.092]	0.081 [0.053 - 0.121]	0.030 [0.024 - 0.038]	0.037 [0.033 - 0.040]	0.047 [0.039 - 0.055]
Age		30-34 yrs			>34 yrs	
Weight gain	Normal weight (n=12,302)	Underweight (n=2,460)	Overweight/Obese (n=4,749)	Normal weight (n=5,750)	Underweight (n=704)	Overweight/Obese (n=3,221)
-5	0.262 [0.134 - 0.450]	0.148 [0.106 - 0.203]	0.099 [0.064 - 0.151]	0.200 [0.065 - 0.472]	0.132 [0.087 - 0.195]	0.140 [0.070 - 0.261]
-4.5	0.244 [0.128 - 0.416]	0.139 [0.101 - 0.189]	0.095 [0.063 - 0.142]	0.191 [0.065 - 0.443]	0.126 [0.084 - 0.183]	0.134 [0.072 - 0.237]
-4	0.227 [0.122 - 0.384]	0.131 [0.096 - 0.175]	0.092 [0.061 - 0.134]	0.182 [0.066 - 0.414]	0.120 [0.082 - 0.172]	0.129 [0.074 - 0.215]
-3.5	0.211 [0.116 - 0.353]	0.123 [0.092 - 0.163]	0.088 [0.060 - 0.127]	0.174 [0.066 - 0.385]	0.114 [0.080 - 0.161]	0.123 [0.075 - 0.196]
-3	0.195 [0.110 - 0.322]	0.115 [0.087 - 0.151]	0.085 [0.059 - 0.120]	0.166 [0.066 - 0.357]	0.109 [0.077 - 0.151]	0.118 [0.076 - 0.179]
-2.5	0.181 [0.105 - 0.294]	0.108 [0.083 - 0.139]	0.082 [0.058 - 0.113]	0.158 [0.066 - 0.331]	0.104 [0.075 - 0.142]	0.112 [0.076 - 0.163]
-2	0.167 [0.100 - 0.267]	0.101 [0.079 - 0.129]	0.078 [0.057 - 0.107]	0.150 [0.067 - 0.305]	0.099 [0.073 - 0.133]	0.107 [0.076 - 0.150]
-1.5	0.154 [0.095 - 0.241]	0.095 [0.075 - 0.119]	0.075 [0.056 - 0.101]	0.143 [0.067 - 0.281]	0.094 [0.071 - 0.124]	0.102 [0.075 - 0.139]
-1	0.142 [0.090 - 0.218]	0.089 [0.071 - 0.110]	0.073 [0.055 - 0.095]	0.136 [0.067 - 0.258]	0.090 [0.069 - 0.116]	0.098 [0.074 - 0.128]
-0.5	0.131 [0.085 - 0.196]	0.083 [0.068 - 0.101]	0.070 [0.054 - 0.089]	0.130 [0.067 - 0.236]	0.086 [0.067 - 0.109]	0.093 [0.072 - 0.119]
Mean	0.121 [0.081 - 0.176]	0.078 [0.064 - 0.093]	0.067 [0.053 - 0.084]	0.123 [0.067 - 0.216]	0.081 [0.065 - 0.102]	0.089 [0.070 - 0.111]
0.5	0.111 [0.077 - 0.157]	0.073 [0.061 - 0.086]	0.064 [0.052 - 0.080]	0.117 [0.067 - 0.198]	0.077 [0.063 - 0.095]	0.084 [0.068 - 0.104]
1	0.102 [0.073 - 0.141]	0.068 [0.058 - 0.079]	0.062 [0.051 - 0.075]	0.112 [0.067 - 0.180]	0.074 [0.061 - 0.089]	0.080 [0.066 - 0.098]
1.5	0.093 [0.069 - 0.126]	0.063 [0.055 - 0.073]	0.059 [0.050 - 0.071]	0.106 [0.067 - 0.165]	0.070 [0.059 - 0.083]	0.076 [0.063 - 0.092]
2	0.086 [0.065 - 0.112]	0.059 [0.052 - 0.068]	0.057 [0.048 - 0.067]	0.101 [0.066 - 0.150]	0.067 [0.057 - 0.078]	0.072 [0.060 - 0.087]
2.5	0.078 [0.061 - 0.100]	0.055 [0.049 - 0.062]	0.055 [0.047 - 0.064]	0.096 [0.066 - 0.137]	0.063 [0.055 - 0.073]	0.069 [0.058 - 0.082]
3	0.072 [0.057 - 0.090]	0.052 [0.046 - 0.058]	0.053 [0.046 - 0.061]	0.091 [0.065 - 0.126]	0.060 [0.053 - 0.069]	0.065 [0.055 - 0.078]
3.5	0.066 [0.053 - 0.081]	0.048 [0.044 - 0.053]	0.051 [0.044 - 0.058]	0.086 [0.063 - 0.116]	0.057 [0.051 - 0.065]	0.062 [0.052 - 0.074]
4	0.060 [0.050 - 0.073]	0.045 [0.041 - 0.049]	0.049 [0.043 - 0.055]	0.082 [0.062 - 0.108]	0.055 [0.049 - 0.061]	0.059 [0.050 - 0.070]
4.5	0.055 [0.046 - 0.066]	0.042 [0.038 - 0.046]	0.047 [0.041 - 0.053]	0.078 [0.059 - 0.102]	0.052 [0.046 - 0.058]	0.057 [0.048 - 0.068]
5	0.050 [0.042 - 0.060]	0.039 [0.036 - 0.043]	0.045 [0.039 - 0.052]	0.074 [0.056 - 0.096]	0.049 [0.044 - 0.055]	0.055 [0.045 - 0.065]

2.5

Stillbirth

Age		<25 yrs			25-29 yrs	
Weight gain	Normal weight (n=1,324)	Underweight (n=698)	Overweight/Obese (n=314)	Normal weight (n=12,406)	Underweight (n=3,943)	Overweight/Obese (n=3,249)
-5	0.004 [0.000 - 0.698]	0.610 [0.027 - 0.989]	0.000 [0.000 - NaN]	0.000 [0.000 - 1.000]	0.001 [0.000 - 0.016]	0.002 [0.000 - 0.038]
-4.5	0.004 [0.000 - 0.639]	0.530 [0.025 - 0.980]	0.000 [0.000 - NaN]	0.000 [0.000 - 1.000]	0.001 [0.000 - 0.014]	0.002 [0.000 - 0.032]
-4	0.004 [0.000 - 0.575]	0.449 [0.023 - 0.965]	0.000 [0.000 - NaN]	0.000 [0.000 - 1.000]	0.001 [0.000 - 0.012]	0.002 [0.000 - 0.028]
-3.5	0.004 [0.000 - 0.509]	0.370 [0.022 - 0.939]	0.000 [0.000 - NaN]	0.000 [0.000 - 1.000]	0.001 [0.000 - 0.011]	0.002 [0.000 - 0.024]
-3	0.004 [0.000 - 0.443]	0.297 [0.020 - 0.896]	0.000 [0.000 - NaN]	0.000 [0.000 - 1.000]	0.001 [0.000 - 0.009]	0.002 [0.000 - 0.021]
-2.5	0.004 [0.000 - 0.379]	0.234 [0.019 - 0.829]	0.000 [0.000 - NaN]	0.000 [0.000 - 0.998]	0.001 [0.000 - 0.008]	0.002 [0.000 - 0.018]
-2	0.004 [0.000 - 0.320]	0.180 [0.017 - 0.733]	0.000 [0.000 - NaN]	0.000 [0.000 - 0.993]	0.001 [0.000 - 0.007]	0.002 [0.000 - 0.016]
-1.5	0.004 [0.000 - 0.266]	0.137 [0.016 - 0.610]	0.000 [0.000 - NaN]	0.000 [0.000 - 0.969]	0.001 [0.000 - 0.006]	0.002 [0.000 - 0.014]
-1	0.004 [0.000 - 0.218]	0.103 [0.014 - 0.475]	0.000 [0.000 - NaN]	0.000 [0.000 - 0.883]	0.001 [0.000 - 0.005]	0.002 [0.000 - 0.012]
-0.5	0.004 [0.000 - 0.177]	0.076 [0.013 - 0.346]	0.000 [0.000 - NaN]	0.000 [0.000 - 0.669]	0.001 [0.000 - 0.005]	0.002 [0.000 - 0.011]
Mean	0.003 [0.000 - 0.143]	0.056 [0.011 - 0.241]	0.000 [0.000 - NaN]	0.000 [0.000 - 0.383]	0.001 [0.000 - 0.004]	0.002 [0.000 - 0.010]
0.5	0.003 [0.000 - 0.114]	0.041 [0.010 - 0.163]	0.000 [0.000 - NaN]	0.001 [0.000 - 0.180]	0.001 [0.000 - 0.004]	0.002 [0.000 - 0.009]
1	0.003 [0.000 - 0.091]	0.030 [0.008 - 0.109]	0.000 [0.000 - NaN]	0.001 [0.000 - 0.081]	0.001 [0.000 - 0.003]	0.002 [0.001 - 0.008]
1.5	0.003 [0.000 - 0.073]	0.023 [0.007 - 0.074]	0.000 [0.000 - NaN]	0.001 [0.000 - 0.039]	0.001 [0.000 - 0.003]	0.002 [0.001 - 0.007]
2	0.003 [0.000 - 0.058]	0.017 [0.005 - 0.051]	0.000 [0.000 - NaN]	0.001 [0.000 - 0.021]	0.001 [0.000 - 0.003]	0.002 [0.001 - 0.007]
2.5	0.003 [0.000 - 0.046]	0.012 [0.004 - 0.036]	0.000 [0.000 - NaN]	0.001 [0.000 - 0.013]	0.001 [0.000 - 0.002]	0.002 [0.001 - 0.006]
3	0.003 [0.000 - 0.037]	0.009 [0.003 - 0.026]	0.000 [0.000 - NaN]	0.001 [0.000 - 0.009]	0.001 [0.000 - 0.002]	0.002 [0.001 - 0.006]
3.5	0.003 [0.000 - 0.030]	0.007 [0.002 - 0.020]	0.000 [0.000 - NaN]	0.002 [0.000 - 0.007]	0.001 [0.000 - 0.002]	0.002 [0.001 - 0.006]
4	0.003 [0.000 - 0.024]	0.005 [0.002 - 0.015]	0.000 [0.000 - NaN]	0.002 [0.000 - 0.007]	0.001 [0.000 - 0.002]	0.002 [0.001 - 0.005]
4.5	0.003 [0.001 - 0.020]	0.004 [0.001 - 0.012]	0.000 [0.000 - NaN]	0.002 [0.000 - 0.006]	0.001 [0.000 - 0.002]	0.003 [0.001 - 0.005]
5	0.003 [0.001 - 0.017]	0.003 [0.001 - 0.010]	0.000 [0.000 - NaN]	0.002 [0.001 - 0.006]	0.001 [0.000 - 0.001]	0.003 [0.001 - 0.005]
Age		30-34 yrs			>34 yrs	
Weight gain	Normal weight (n=12,302)	Underweight (n=2,460)	Overweight/Obese (n=4,749)	Normal weight (n=5,750)	Underweight (n=704)	Overweight/Obese (n=3,221)
-5	0.000 [0.000 - 1.000]	0.006 [0.001 - 0.050]	0.002 [0.000 - 0.021]	0.038 [0.000 - 0.806]	0.016 [0.002 - 0.119]	0.000 [0.000 - NaN]
-4.5	0.000 [0.000 - 1.000]	0.005 [0.001 - 0.041]	0.002 [0.000 - 0.019]	0.034 [0.000 - 0.745]	0.015 [0.002 - 0.097]	0.000 [0.000 - NaN]
-4	0.000 [0.000 - 1.000]	0.005 [0.001 - 0.034]	0.002 [0.000 - 0.017]	0.030 [0.000 - 0.672]	0.013 [0.002 - 0.079]	0.000 [0.000 - NaN]
-3.5	0.000 [0.000 - 1.000]	0.004 [0.001 - 0.028]	0.002 [0.000 - 0.015]	0.027 [0.001 - 0.591]	0.011 [0.002 - 0.063]	0.000 [0.000 - NaN]
-3	0.000 [0.000 - 1.000]	0.004 [0.001 - 0.023]	0.002 [0.000 - 0.014]	0.024 [0.001 - 0.505]	0.010 [0.002 - 0.051]	0.000 [0.000 - NaN]
-2.5	0.000 [0.000 - 1.000]	0.004 [0.001 - 0.019]	0.002 [0.000 - 0.012]	0.022 [0.001 - 0.419]	0.009 [0.002 - 0.041]	0.000 [0.000 - NaN]
-2	0.000 [0.000 - 1.000]	0.003 [0.001 - 0.016]	0.002 [0.000 - 0.011]	0.020 [0.001 - 0.338]	0.008 [0.002 - 0.033]	0.000 [0.000 - NaN]
-1.5	0.000 [0.000 - 1.000]	0.003 [0.001 - 0.013]	0.002 [0.000 - 0.010]	0.018 [0.001 - 0.266]	0.007 [0.002 - 0.027]	0.000 [0.000 - NaN]
-1	0.000 [0.000 - 1.000]	0.003 [0.001 - 0.011]	0.002 [0.000 - 0.009]	0.016 [0.001 - 0.205]	0.006 [0.002 - 0.022]	0.000 [0.000 - NaN]
-0.5	0.000 [0.000 - 1.000]	0.003 [0.001 - 0.009]	0.002 [0.000 - 0.008]	0.014 [0.001 - 0.155]	0.006 [0.002 - 0.017]	0.000 [0.000 - NaN]
Mean	0.000 [0.000 - 1.000]	0.002 [0.001 - 0.008]	0.002 [0.001 - 0.007]	0.013 [0.001 - 0.117]	0.005 [0.002 - 0.014]	0.000 [0.000 - NaN]
0.5	0.000 [0.000 - 1.000]	0.002 [0.001 - 0.006]	0.002 [0.001 - 0.007]	0.011 [0.001 - 0.087]	0.004 [0.002 - 0.011]	0.000 [0.000 - NaN]
1	0.000 [0.000 - 1.000]	0.002 [0.001 - 0.005]	0.002 [0.001 - 0.006]	0.010 [0.002 - 0.065]	0.004 [0.002 - 0.009]	0.000 [0.000 - NaN]
1.5	0.000 [0.000 - 0.994]	0.002 [0.001 - 0.004]	0.002 [0.001 - 0.005]	0.009 [0.002 - 0.049]	0.004 [0.002 - 0.008]	0.000 [0.000 - NaN]
2	0.000 [0.000 - 0.916]	0.002 [0.001 - 0.004]	0.002 [0.001 - 0.005]	0.008 [0.002 - 0.037]	0.003 [0.002 - 0.006]	0.000 [0.000 - NaN]
2.5	0.000 [0.000 - 0.512]	0.002 [0.001 - 0.003]	0.002 [0.001 - 0.005]	0.007 [0.002 - 0.029]	0.003 [0.001 - 0.005]	0.000 [0.000 - NaN]
3	0.000 [0.000 - 0.136]	0.001 [0.001 - 0.003]	0.002 [0.001 - 0.004]	0.007 [0.002 - 0.023]	0.002 [0.001 - 0.005]	0.000 [0.000 - NaN]
3.5	0.000 [0.000 - 0.038]	0.001 [0.001 - 0.002]	0.002 [0.001 - 0.004]	0.006 [0.002 - 0.019]	0.002 [0.001 - 0.004]	0.000 [0.000 - NaN]
4	0.000 [0.000 - 0.017]	0.001 [0.001 - 0.002]	0.002 [0.001 - 0.004]	0.005 [0.002 - 0.016]	0.002 [0.001 - 0.004]	0.000 [0.000 - NaN]
4.5	0.001 [0.000 - 0.011]	0.001 [0.001 - 0.002]	0.002 [0.001 - 0.004]	0.005 [0.001 - 0.015]	0.002 [0.001 - 0.003]	0.000 [0.000 - NaN]
5	0.002 [0.000 - 0.011]	0.001 [0.001 - 0.002]	0.002 [0.001 - 0.004]	0.004 [0.001 - 0.014]	0.002 [0.001 - 0.003]	0.000 [0.000 - NaN]

2.6

Macrosomia

Age		<25 yrs			25-29 yrs	
Weight gain	Normal weight (n=1,317)	Underweight (n=697)	Overweight/Obese (n=313)	Normal weight (n=12,390)	Underweight (n=3,940)	Overweight/Obese (n=3,236)
-5	0.003 [0.000 - 0.076]	0.004 [0.001 - 0.021]	0.075 [0.013 - 0.335]	0.001 [0.000 - 0.004]	0.007 [0.004 - 0.011]	0.026 [0.015 - 0.045]
-4.5	0.003 [0.000 - 0.072]	0.005 [0.001 - 0.021]	0.075 [0.014 - 0.315]	0.001 [0.000 - 0.004]	0.007 [0.005 - 0.011]	0.028 [0.016 - 0.046]
-4	0.003 [0.000 - 0.067]	0.005 [0.001 - 0.022]	0.075 [0.015 - 0.296]	0.001 [0.001 - 0.004]	0.008 [0.005 - 0.012]	0.029 [0.018 - 0.047]
-3.5	0.004 [0.000 - 0.063]	0.005 [0.001 - 0.022]	0.075 [0.017 - 0.277]	0.002 [0.001 - 0.005]	0.008 [0.006 - 0.012]	0.030 [0.019 - 0.049]
-3	0.004 [0.000 - 0.060]	0.006 [0.002 - 0.022]	0.075 [0.019 - 0.260]	0.002 [0.001 - 0.005]	0.009 [0.006 - 0.013]	0.032 [0.020 - 0.050]
-2.5	0.004 [0.000 - 0.056]	0.007 [0.002 - 0.023]	0.076 [0.020 - 0.244]	0.002 [0.001 - 0.005]	0.010 [0.007 - 0.014]	0.034 [0.022 - 0.051]
-2	0.004 [0.000 - 0.053]	0.007 [0.002 - 0.024]	0.076 [0.022 - 0.228]	0.002 [0.001 - 0.006]	0.010 [0.007 - 0.015]	0.035 [0.024 - 0.053]
-1.5	0.004 [0.000 - 0.050]	0.008 [0.002 - 0.024]	0.076 [0.024 - 0.215]	0.003 [0.001 - 0.006]	0.011 [0.008 - 0.016]	0.037 [0.025 - 0.054]
-1	0.005 [0.000 - 0.047]	0.009 [0.003 - 0.025]	0.076 [0.026 - 0.202]	0.003 [0.001 - 0.006]	0.012 [0.009 - 0.017]	0.039 [0.027 - 0.055]
-0.5	0.005 [0.001 - 0.044]	0.009 [0.003 - 0.025]	0.076 [0.028 - 0.190]	0.003 [0.001 - 0.007]	0.013 [0.010 - 0.018]	0.041 [0.029 - 0.057]
Mean	0.005 [0.001 - 0.041]	0.010 [0.004 - 0.026]	0.077 [0.030 - 0.179]	0.004 [0.002 - 0.007]	0.014 [0.011 - 0.019]	0.043 [0.031 - 0.058]
0.5	0.006 [0.001 - 0.039]	0.011 [0.005 - 0.027]	0.077 [0.033 - 0.169]	0.004 [0.002 - 0.008]	0.015 [0.012 - 0.020]	0.045 [0.034 - 0.060]
1	0.006 [0.001 - 0.037]	0.012 [0.005 - 0.028]	0.077 [0.035 - 0.161]	0.004 [0.002 - 0.008]	0.016 [0.013 - 0.021]	0.047 [0.036 - 0.062]
1.5	0.006 [0.001 - 0.035]	0.013 [0.006 - 0.029]	0.077 [0.037 - 0.153]	0.005 [0.003 - 0.009]	0.018 [0.014 - 0.022]	0.050 [0.039 - 0.063]
2	0.007 [0.001 - 0.033]	0.015 [0.007 - 0.030]	0.077 [0.040 - 0.146]	0.005 [0.003 - 0.010]	0.019 [0.016 - 0.023]	0.052 [0.041 - 0.065]
2.5	0.007 [0.002 - 0.031]	0.016 [0.008 - 0.031]	0.078 [0.042 - 0.139]	0.006 [0.004 - 0.010]	0.021 [0.017 - 0.025]	0.054 [0.044 - 0.067]
3	0.007 [0.002 - 0.030]	0.018 [0.010 - 0.032]	0.078 [0.044 - 0.134]	0.007 [0.004 - 0.011]	0.022 [0.019 - 0.026]	0.057 [0.047 - 0.069]
3.5	0.008 [0.002 - 0.028]	0.019 [0.011 - 0.033]	0.078 [0.046 - 0.130]	0.008 [0.005 - 0.012]	0.024 [0.020 - 0.028]	0.060 [0.050 - 0.071]
4	0.008 [0.003 - 0.027]	0.021 [0.013 - 0.035]	0.079 [0.048 - 0.126]	0.008 [0.006 - 0.013]	0.026 [0.022 - 0.030]	0.063 [0.054 - 0.073]
4.5	0.009 [0.003 - 0.026]	0.023 [0.014 - 0.036]	0.079 [0.050 - 0.123]	0.009 [0.006 - 0.014]	0.028 [0.024 - 0.031]	0.066 [0.057 - 0.076]
5	0.009 [0.004 - 0.025]	0.025 [0.016 - 0.038]	0.080 [0.052 - 0.122]	0.011 [0.007 - 0.015]	0.030 [0.026 - 0.033]	0.069 [0.060 - 0.079]
Age		30-34 yrs			>34 yrs	
Weight gain	Normal weight (n=12,281)	Underweight (n=2,458)	Overweight/Obese (n=4,738)	Normal weight (n=5,735)	Underweight (n=703)	Overweight/Obese (n=3,211)
-5	0.000 [0.000 - 0.141]	0.007 [0.005 - 0.011]	0.016 [0.006 - 0.044]	0.001 [0.000 - 0.029]	0.012 [0.006 - 0.022]	0.013 [0.004 - 0.043]
-4.5	0.000 [0.000 - 0.111]	0.008 [0.005 - 0.012]	0.017 [0.007 - 0.043]	0.001 [0.000 - 0.028]	0.012 [0.007 - 0.022]	0.014 [0.005 - 0.042]
-4	0.000 [0.000 - 0.087]	0.008 [0.006 - 0.013]	0.019 [0.008 - 0.043]	0.001 [0.000 - 0.027]	0.013 [0.007 - 0.023]	0.016 [0.006 - 0.042]
-3.5	0.000 [0.000 - 0.069]	0.009 [0.006 - 0.013]	0.020 [0.009 - 0.043]	0.001 [0.000 - 0.026]	0.014 [0.008 - 0.023]	0.017 [0.007 - 0.042]
-3	0.000 [0.000 - 0.055]	0.010 [0.007 - 0.014]	0.022 [0.011 - 0.043]	0.001 [0.000 - 0.025]	0.015 [0.009 - 0.024]	0.019 [0.009 - 0.043]
-2.5	0.000 [0.000 - 0.044]	0.011 [0.008 - 0.015]	0.024 [0.013 - 0.044]	0.001 [0.000 - 0.025]	0.015 [0.009 - 0.025]	0.021 [0.010 - 0.043]
-2	0.001 [0.000 - 0.036]	0.012 [0.008 - 0.016]	0.026 [0.015 - 0.044]	0.001 [0.000 - 0.024]	0.016 [0.010 - 0.025]	0.024 [0.013 - 0.044]
-1.5	0.001 [0.000 - 0.030]	0.013 [0.009 - 0.017]	0.028 [0.017 - 0.045]	0.001 [0.000 - 0.023]	0.017 [0.011 - 0.026]	0.026 [0.015 - 0.045]
-1	0.001 [0.000 - 0.025]	0.014 [0.010 - 0.018]	0.030 [0.019 - 0.046]	0.002 [0.000 - 0.022]	0.018 [0.012 - 0.027]	0.029 [0.018 - 0.047]
-0.5	0.001 [0.000 - 0.022]	0.015 [0.011 - 0.019]	0.032 [0.022 - 0.048]	0.002 [0.000 - 0.022]	0.019 [0.013 - 0.028]	0.032 [0.021 - 0.049]
Mean	0.002 [0.000 - 0.019]	0.016 [0.012 - 0.020]	0.035 [0.025 - 0.049]	0.002 [0.000 - 0.021]	0.020 [0.014 - 0.028]	0.035 [0.024 - 0.051]
0.5	0.002 [0.000 - 0.018]	0.017 [0.014 - 0.022]	0.038 [0.028 - 0.051]	0.002 [0.000 - 0.020]	0.021 [0.015 - 0.029]	0.039 [0.028 - 0.053]
1	0.003 [0.000 - 0.016]	0.018 [0.015 - 0.023]	0.041 [0.032 - 0.053]	0.003 [0.000 - 0.020]	0.022 [0.017 - 0.030]	0.043 [0.032 - 0.056]
1.5	0.004 [0.001 - 0.016]	0.020 [0.016 - 0.024]	0.044 [0.035 - 0.055]	0.003 [0.000 - 0.019]	0.024 [0.018 - 0.031]	0.046 [0.036 - 0.059]
2	0.005 [0.001 - 0.016]	0.022 [0.018 - 0.026]	0.048 [0.039 - 0.058]	0.003 [0.001 - 0.019]	0.025 [0.019 - 0.032]	0.050 [0.040 - 0.063]
2.5	0.006 [0.002 - 0.016]	0.023 [0.020 - 0.028]	0.052 [0.043 - 0.062]	0.004 [0.001 - 0.019]	0.026 [0.021 - 0.033]	0.054 [0.044 - 0.066]
3	0.008 [0.003 - 0.017]	0.025 [0.022 - 0.029]	0.056 [0.047 - 0.065]	0.004 [0.001 - 0.018]	0.028 [0.023 - 0.034]	0.058 [0.048 - 0.070]
3.5	0.010 [0.005 - 0.019]	0.027 [0.024 - 0.031]	0.060 [0.052 - 0.069]	0.005 [0.001 - 0.018]	0.029 [0.024 - 0.035]	0.062 [0.052 - 0.074]
4	0.012 [0.007 - 0.021]	0.029 [0.026 - 0.033]	0.064 [0.056 - 0.073]	0.006 [0.002 - 0.018]	0.031 [0.026 - 0.036]	0.065 [0.055 - 0.077]
4.5	0.014 [0.009 - 0.023]	0.032 [0.028 - 0.035]	0.068 [0.060 - 0.078]	0.006 [0.002 - 0.018]	0.032 [0.028 - 0.038]	0.069 [0.058 - 0.081]
5	0.017 [0.011 - 0.026]	0.034 [0.031 - 0.038]	0.073 [0.064 - 0.083]	0.007 [0.003 - 0.019]	0.034 [0.030 - 0.039]	0.072 [0.061 - 0.085]

2.7

Small for gestational age

Age		<25 yrs			25-29 yrs	
Weight gain	Normal weight (n=1,316)	Underweight (n=697)	Overweight/Obese (n=313)	Normal weight (n=12,386)	Underweight (n=3,938)	Overweight/Obese (n=3,236)
-5	0.089 [0.023 - 0.290]	0.205 [0.043 - 0.597]	0.093 [0.011 - 0.491]	0.257 [0.070 - 0.614]	0.080 [0.053 - 0.119]	0.026 [0.011 - 0.065]
-4.5	0.088 [0.024 - 0.275]	0.190 [0.043 - 0.551]	0.089 [0.011 - 0.453]	0.240 [0.071 - 0.566]	0.077 [0.052 - 0.113]	0.026 [0.011 - 0.061]
-4	0.087 [0.025 - 0.261]	0.177 [0.043 - 0.505]	0.084 [0.012 - 0.415]	0.224 [0.072 - 0.517]	0.074 [0.051 - 0.107]	0.026 [0.011 - 0.059]
-3.5	0.086 [0.026 - 0.248]	0.164 [0.044 - 0.458]	0.080 [0.012 - 0.379]	0.208 [0.073 - 0.467]	0.072 [0.050 - 0.101]	0.026 [0.012 - 0.056]
-3	0.085 [0.027 - 0.235]	0.152 [0.044 - 0.412]	0.076 [0.013 - 0.344]	0.193 [0.074 - 0.419]	0.069 [0.049 - 0.096]	0.025 [0.012 - 0.053]
-2.5	0.084 [0.028 - 0.222]	0.141 [0.044 - 0.368]	0.073 [0.013 - 0.311]	0.179 [0.075 - 0.373]	0.066 [0.048 - 0.091]	0.025 [0.012 - 0.051]
-2	0.083 [0.030 - 0.210]	0.130 [0.044 - 0.327]	0.069 [0.014 - 0.280]	0.166 [0.075 - 0.329]	0.064 [0.047 - 0.086]	0.025 [0.013 - 0.048]
-1.5	0.082 [0.031 - 0.199]	0.120 [0.044 - 0.288]	0.066 [0.015 - 0.251]	0.154 [0.076 - 0.289]	0.061 [0.046 - 0.081]	0.025 [0.013 - 0.046]
-1	0.081 [0.032 - 0.188]	0.111 [0.044 - 0.253]	0.063 [0.015 - 0.224]	0.142 [0.076 - 0.252]	0.059 [0.045 - 0.077]	0.024 [0.014 - 0.044]
-0.5	0.080 [0.034 - 0.178]	0.103 [0.044 - 0.221]	0.060 [0.016 - 0.200]	0.132 [0.075 - 0.220]	0.057 [0.044 - 0.073]	0.024 [0.014 - 0.042]
Mean	0.079 [0.035 - 0.168]	0.095 [0.044 - 0.193]	0.057 [0.016 - 0.177]	0.121 [0.074 - 0.192]	0.055 [0.043 - 0.069]	0.024 [0.014 - 0.040]
0.5	0.078 [0.036 - 0.159]	0.087 [0.043 - 0.169]	0.054 [0.017 - 0.158]	0.112 [0.073 - 0.168]	0.052 [0.042 - 0.065]	0.024 [0.015 - 0.038]
1	0.077 [0.038 - 0.150]	0.081 [0.042 - 0.147]	0.051 [0.018 - 0.140]	0.103 [0.071 - 0.147]	0.050 [0.041 - 0.062]	0.024 [0.015 - 0.036]
1.5	0.076 [0.039 - 0.141]	0.074 [0.042 - 0.129]	0.049 [0.018 - 0.125]	0.095 [0.069 - 0.130]	0.049 [0.040 - 0.058]	0.023 [0.016 - 0.035]
2	0.075 [0.041 - 0.134]	0.069 [0.041 - 0.113]	0.046 [0.019 - 0.111]	0.088 [0.066 - 0.115]	0.047 [0.039 - 0.055]	0.023 [0.016 - 0.033]
2.5	0.074 [0.042 - 0.126]	0.063 [0.039 - 0.100]	0.044 [0.019 - 0.099]	0.081 [0.063 - 0.102]	0.045 [0.038 - 0.052]	0.023 [0.016 - 0.032]
3	0.073 [0.044 - 0.119]	0.059 [0.038 - 0.089]	0.042 [0.019 - 0.089]	0.075 [0.060 - 0.092]	0.043 [0.037 - 0.049]	0.023 [0.017 - 0.031]
3.5	0.072 [0.045 - 0.113]	0.054 [0.037 - 0.080]	0.040 [0.019 - 0.081]	0.069 [0.057 - 0.083]	0.041 [0.037 - 0.047]	0.022 [0.017 - 0.030]
4	0.071 [0.047 - 0.107]	0.050 [0.035 - 0.072]	0.038 [0.019 - 0.074]	0.064 [0.054 - 0.076]	0.040 [0.035 - 0.045]	0.022 [0.017 - 0.029]
4.5	0.070 [0.048 - 0.102]	0.047 [0.034 - 0.065]	0.036 [0.019 - 0.069]	0.060 [0.051 - 0.071]	0.038 [0.034 - 0.042]	0.022 [0.017 - 0.028]
5	0.069 [0.049 - 0.098]	0.044 [0.032 - 0.060]	0.034 [0.018 - 0.064]	0.056 [0.048 - 0.066]	0.037 [0.033 - 0.041]	0.022 [0.017 - 0.028]
Age		30-34 yrs			>34 yrs	
Weight gain	Normal weight (n=12,275)	Underweight (n=2,458)	Overweight/Obese (n=4,735)	Normal weight (n=5,730)	Underweight (n=703)	Overweight/Obese (n=3,208)
-5	0.369 [0.085 - 0.787]	0.084 [0.056 - 0.123]	0.025 [0.012 - 0.050]	0.112 [0.028 - 0.353]	0.037 [0.019 - 0.071]	0.007 [0.001 - 0.051]
-4.5	0.339 [0.087 - 0.734]	0.080 [0.055 - 0.115]	0.025 [0.012 - 0.048]	0.108 [0.029 - 0.329]	0.037 [0.020 - 0.068]	0.007 [0.001 - 0.046]
-4	0.310 [0.089 - 0.675]	0.076 [0.053 - 0.108]	0.024 [0.013 - 0.046]	0.104 [0.030 - 0.305]	0.036 [0.020 - 0.064]	0.008 [0.001 - 0.041]
-3.5	0.282 [0.090 - 0.610]	0.073 [0.052 - 0.102]	0.024 [0.013 - 0.044]	0.101 [0.031 - 0.283]	0.035 [0.020 - 0.061]	0.008 [0.002 - 0.037]
-3	0.256 [0.091 - 0.543]	0.070 [0.050 - 0.096]	0.024 [0.014 - 0.043]	0.098 [0.032 - 0.262]	0.035 [0.020 - 0.058]	0.009 [0.002 - 0.034]
-2.5	0.232 [0.091 - 0.475]	0.067 [0.049 - 0.090]	0.024 [0.014 - 0.041]	0.094 [0.033 - 0.242]	0.034 [0.021 - 0.056]	0.010 [0.003 - 0.032]
-2	0.209 [0.091 - 0.411]	0.064 [0.048 - 0.084]	0.024 [0.014 - 0.039]	0.091 [0.034 - 0.223]	0.033 [0.021 - 0.053]	0.011 [0.004 - 0.030]
-1.5	0.188 [0.090 - 0.352]	0.061 [0.046 - 0.079]	0.024 [0.015 - 0.038]	0.088 [0.035 - 0.205]	0.033 [0.021 - 0.051]	0.011 [0.005 - 0.028]
-1	0.168 [0.088 - 0.299]	0.058 [0.045 - 0.074]	0.023 [0.015 - 0.037]	0.085 [0.036 - 0.188]	0.032 [0.021 - 0.048]	0.012 [0.005 - 0.027]
-0.5	0.151 [0.085 - 0.253]	0.055 [0.044 - 0.070]	0.023 [0.015 - 0.035]	0.082 [0.037 - 0.173]	0.032 [0.022 - 0.046]	0.013 [0.006 - 0.026]
Mean	0.135 [0.081 - 0.214]	0.053 [0.042 - 0.066]	0.023 [0.016 - 0.034]	0.079 [0.038 - 0.159]	0.031 [0.022 - 0.044]	0.014 [0.008 - 0.026]
0.5	0.120 [0.078 - 0.182]	0.050 [0.041 - 0.062]	0.023 [0.016 - 0.033]	0.077 [0.039 - 0.145]	0.030 [0.022 - 0.042]	0.015 [0.009 - 0.025]
1	0.108 [0.073 - 0.155]	0.048 [0.040 - 0.058]	0.023 [0.017 - 0.032]	0.074 [0.040 - 0.133]	0.030 [0.022 - 0.040]	0.016 [0.010 - 0.025]
1.5	0.097 [0.069 - 0.134]	0.046 [0.039 - 0.054]	0.023 [0.017 - 0.031]	0.072 [0.041 - 0.122]	0.029 [0.022 - 0.038]	0.017 [0.011 - 0.026]
2	0.087 [0.065 - 0.116]	0.044 [0.038 - 0.051]	0.023 [0.017 - 0.030]	0.069 [0.042 - 0.112]	0.029 [0.022 - 0.037]	0.018 [0.012 - 0.026]
2.5	0.079 [0.061 - 0.103]	0.042 [0.036 - 0.048]	0.022 [0.018 - 0.029]	0.067 [0.042 - 0.104]	0.028 [0.023 - 0.035]	0.018 [0.013 - 0.026]
3	0.073 [0.057 - 0.092]	0.040 [0.035 - 0.045]	0.022 [0.018 - 0.028]	0.064 [0.043 - 0.096]	0.028 [0.023 - 0.034]	0.019 [0.013 - 0.027]
3.5	0.067 [0.053 - 0.084]	0.038 [0.034 - 0.043]	0.022 [0.018 - 0.027]	0.062 [0.043 - 0.089]	0.027 [0.022 - 0.033]	0.019 [0.014 - 0.027]
4	0.062 [0.050 - 0.077]	0.036 [0.033 - 0.040]	0.022 [0.018 - 0.027]	0.060 [0.043 - 0.084]	0.027 [0.022 - 0.032]	0.019 [0.014 - 0.027]
4.5	0.058 [0.047 - 0.071]	0.035 [0.031 - 0.038]	0.022 [0.018 - 0.027]	0.058 [0.042 - 0.079]	0.026 [0.022 - 0.031]	0.019 [0.014 - 0.027]
5	0.054 [0.044 - 0.067]	0.033 [0.030 - 0.036]	0.022 [0.018 - 0.027]	0.056 [0.041 - 0.076]	0.026 [0.022 - 0.030]	0.019 [0.014 - 0.027]

2.8

Large for gestational age

Age		<25 yrs			25-29 yrs	
Weight gain	Normal weight (n=1,316)	Underweight (n=697)	Overweight/Obese (n=313)	Normal weight (n=12,386)	Underweight (n=3,938)	Overweight/Obese (n=3,236)
-5	0.067 [0.005 - 0.490]	0.245 [0.012 - 0.899]	0.268 [0.052 - 0.711]	0.021 [0.012 - 0.037]	0.080 [0.036 - 0.168]	0.123 [0.089 - 0.166]
-4.5	0.066 [0.006 - 0.453]	0.239 [0.015 - 0.870]	0.262 [0.057 - 0.677]	0.022 [0.013 - 0.038]	0.081 [0.039 - 0.160]	0.126 [0.093 - 0.168]
-4	0.066 [0.007 - 0.417]	0.234 [0.018 - 0.835]	0.256 [0.063 - 0.640]	0.024 [0.014 - 0.039]	0.081 [0.042 - 0.153]	0.129 [0.096 - 0.169]
-3.5	0.066 [0.008 - 0.382]	0.228 [0.022 - 0.792]	0.250 [0.069 - 0.603]	0.025 [0.015 - 0.041]	0.082 [0.045 - 0.146]	0.132 [0.100 - 0.171]
-3	0.065 [0.009 - 0.348]	0.223 [0.028 - 0.742]	0.245 [0.075 - 0.565]	0.026 [0.016 - 0.042]	0.083 [0.048 - 0.139]	0.135 [0.104 - 0.173]
-2.5	0.065 [0.010 - 0.316]	0.217 [0.034 - 0.686]	0.239 [0.081 - 0.527]	0.028 [0.018 - 0.043]	0.084 [0.052 - 0.134]	0.138 [0.108 - 0.174]
-2	0.064 [0.012 - 0.286]	0.212 [0.042 - 0.624]	0.233 [0.088 - 0.491]	0.029 [0.019 - 0.045]	0.085 [0.055 - 0.128]	0.141 [0.112 - 0.176]
-1.5	0.064 [0.013 - 0.258]	0.207 [0.051 - 0.560]	0.228 [0.094 - 0.456]	0.031 [0.021 - 0.046]	0.086 [0.059 - 0.124]	0.144 [0.117 - 0.178]
-1	0.064 [0.015 - 0.232]	0.202 [0.061 - 0.495]	0.222 [0.099 - 0.424]	0.033 [0.022 - 0.048]	0.087 [0.062 - 0.120]	0.148 [0.121 - 0.179]
-0.5	0.063 [0.017 - 0.209]	0.196 [0.072 - 0.435]	0.216 [0.105 - 0.394]	0.035 [0.024 - 0.049]	0.088 [0.066 - 0.116]	0.151 [0.126 - 0.181]
Mean	0.063 [0.019 - 0.188]	0.191 [0.083 - 0.381]	0.211 [0.110 - 0.366]	0.037 [0.026 - 0.051]	0.088 [0.069 - 0.113]	0.155 [0.130 - 0.183]
0.5	0.063 [0.022 - 0.169]	0.185 [0.092 - 0.335]	0.205 [0.114 - 0.341]	0.039 [0.028 - 0.053]	0.089 [0.072 - 0.110]	0.158 [0.135 - 0.185]
1	0.062 [0.024 - 0.153]	0.178 [0.100 - 0.297]	0.199 [0.117 - 0.319]	0.041 [0.031 - 0.055]	0.090 [0.075 - 0.108]	0.162 [0.140 - 0.187]
1.5	0.062 [0.026 - 0.139]	0.170 [0.104 - 0.264]	0.194 [0.119 - 0.299]	0.043 [0.033 - 0.057]	0.091 [0.078 - 0.106]	0.166 [0.145 - 0.189]
2	0.062 [0.029 - 0.127]	0.161 [0.106 - 0.235]	0.189 [0.121 - 0.282]	0.046 [0.036 - 0.058]	0.092 [0.081 - 0.105]	0.169 [0.150 - 0.191]
2.5	0.061 [0.031 - 0.117]	0.150 [0.105 - 0.210]	0.184 [0.122 - 0.266]	0.048 [0.038 - 0.061]	0.093 [0.083 - 0.105]	0.173 [0.155 - 0.193]
3	0.061 [0.034 - 0.108]	0.139 [0.101 - 0.189]	0.179 [0.123 - 0.254]	0.051 [0.041 - 0.063]	0.095 [0.085 - 0.104]	0.177 [0.160 - 0.196]
3.5	0.060 [0.036 - 0.101]	0.128 [0.095 - 0.170]	0.175 [0.123 - 0.243]	0.054 [0.044 - 0.065]	0.096 [0.088 - 0.105]	0.181 [0.165 - 0.198]
4	0.060 [0.037 - 0.095]	0.117 [0.089 - 0.153]	0.172 [0.123 - 0.235]	0.057 [0.048 - 0.067]	0.098 [0.090 - 0.106]	0.185 [0.170 - 0.201]
4.5	0.060 [0.039 - 0.090]	0.108 [0.083 - 0.140]	0.169 [0.122 - 0.228]	0.060 [0.051 - 0.070]	0.100 [0.093 - 0.108]	0.189 [0.175 - 0.205]
5	0.059 [0.040 - 0.087]	0.101 [0.078 - 0.129]	0.166 [0.122 - 0.223]	0.063 [0.055 - 0.073]	0.103 [0.096 - 0.111]	0.194 [0.180 - 0.208]

Age		30-34 yrs			>34 yrs	
Weight gain	Normal weight (n=12,275)	Underweight (n=2,458)	Overweight/Obese (n=4,735)	Normal weight (n=5,730)	Underweight (n=703)	Overweight/Obese (n=3,208)
-5	0.021 [0.010 - 0.041]	0.052 [0.041 - 0.065]	0.103 [0.080 - 0.132]	0.024 [0.007 - 0.081]	0.096 [0.072 - 0.127]	0.133 [0.102 - 0.171]
-4.5	0.022 [0.012 - 0.042]	0.054 [0.043 - 0.067]	0.107 [0.084 - 0.135]	0.026 [0.008 - 0.082]	0.098 [0.074 - 0.129]	0.137 [0.107 - 0.174]
-4	0.024 [0.013 - 0.044]	0.056 [0.045 - 0.070]	0.111 [0.088 - 0.138]	0.028 [0.009 - 0.083]	0.100 [0.077 - 0.130]	0.142 [0.112 - 0.178]
-3.5	0.025 [0.014 - 0.045]	0.059 [0.048 - 0.072]	0.115 [0.093 - 0.142]	0.030 [0.010 - 0.083]	0.103 [0.080 - 0.131]	0.146 [0.117 - 0.181]
-3	0.027 [0.015 - 0.047]	0.061 [0.051 - 0.074]	0.119 [0.098 - 0.145]	0.032 [0.011 - 0.084]	0.105 [0.083 - 0.132]	0.151 [0.122 - 0.184]
-2.5	0.029 [0.017 - 0.049]	0.064 [0.053 - 0.077]	0.124 [0.102 - 0.149]	0.034 [0.013 - 0.085]	0.107 [0.086 - 0.133]	0.155 [0.128 - 0.187]
-2	0.031 [0.018 - 0.051]	0.067 [0.056 - 0.079]	0.129 [0.108 - 0.153]	0.036 [0.015 - 0.086]	0.110 [0.089 - 0.134]	0.160 [0.133 - 0.191]
-1.5	0.033 [0.020 - 0.052]	0.070 [0.060 - 0.082]	0.133 [0.113 - 0.157]	0.038 [0.016 - 0.087]	0.112 [0.092 - 0.135]	0.165 [0.139 - 0.194]
-1	0.035 [0.022 - 0.054]	0.073 [0.063 - 0.085]	0.138 [0.118 - 0.161]	0.041 [0.018 - 0.088]	0.115 [0.096 - 0.137]	0.170 [0.145 - 0.198]
-0.5	0.037 [0.024 - 0.056]	0.076 [0.066 - 0.088]	0.143 [0.124 - 0.165]	0.044 [0.021 - 0.089]	0.117 [0.099 - 0.138]	0.175 [0.152 - 0.201]
Mean	0.040 [0.027 - 0.059]	0.080 [0.070 - 0.091]	0.149 [0.130 - 0.169]	0.046 [0.023 - 0.090]	0.120 [0.102 - 0.139]	0.181 [0.158 - 0.205]
0.5	0.042 [0.029 - 0.061]	0.083 [0.074 - 0.094]	0.154 [0.136 - 0.173]	0.049 [0.026 - 0.091]	0.122 [0.106 - 0.140]	0.186 [0.165 - 0.209]
1	0.045 [0.032 - 0.063]	0.087 [0.078 - 0.097]	0.159 [0.143 - 0.178]	0.053 [0.029 - 0.092]	0.125 [0.110 - 0.142]	0.191 [0.172 - 0.213]
1.5	0.048 [0.035 - 0.065]	0.090 [0.082 - 0.100]	0.165 [0.149 - 0.182]	0.056 [0.033 - 0.094]	0.128 [0.113 - 0.143]	0.197 [0.178 - 0.217]
2	0.051 [0.039 - 0.068]	0.094 [0.086 - 0.103]	0.171 [0.156 - 0.187]	0.060 [0.037 - 0.095]	0.130 [0.117 - 0.145]	0.203 [0.185 - 0.222]
2.5	0.055 [0.042 - 0.071]	0.098 [0.090 - 0.107]	0.177 [0.163 - 0.192]	0.063 [0.041 - 0.097]	0.133 [0.121 - 0.146]	0.209 [0.192 - 0.227]
3	0.058 [0.046 - 0.073]	0.103 [0.095 - 0.110]	0.183 [0.170 - 0.197]	0.067 [0.046 - 0.099]	0.136 [0.125 - 0.148]	0.215 [0.199 - 0.232]
3.5	0.062 [0.050 - 0.076]	0.107 [0.100 - 0.114]	0.190 [0.177 - 0.203]	0.072 [0.050 - 0.101]	0.139 [0.128 - 0.150]	0.221 [0.206 - 0.237]
4	0.066 [0.054 - 0.080]	0.111 [0.105 - 0.118]	0.196 [0.184 - 0.209]	0.076 [0.056 - 0.104]	0.142 [0.132 - 0.152]	0.227 [0.213 - 0.243]
4.5	0.070 [0.059 - 0.083]	0.116 [0.110 - 0.123]	0.203 [0.191 - 0.215]	0.081 [0.061 - 0.107]	0.145 [0.135 - 0.155]	0.234 [0.219 - 0.250]
5	0.075 [0.064 - 0.087]	0.121 [0.115 - 0.127]	0.210 [0.198 - 0.222]	0.086 [0.066 - 0.111]	0.148 [0.139 - 0.158]	0.240 [0.225 - 0.257]

2.9

All disease

Age		<25 yrs			25-29 yrs	
Weight gain	Normal weight (n=1,324)	Underweight (n=698)	Overweight/Obese (n=314)	Normal weight (n=12,406)	Underweight (n=3,943)	Overweight/Obese (n=3,249)
-5	0.726 [0.095 - 0.985]	0.842 [0.291 - 0.986]	0.967 [0.555 - 0.999]	0.870 [0.547 - 0.974]	0.870 [0.613 - 0.966]	0.975 [0.881 - 0.995]
-4.5	0.705 [0.105 - 0.980]	0.821 [0.301 - 0.980]	0.960 [0.571 - 0.998]	0.849 [0.537 - 0.965]	0.856 [0.626 - 0.955]	0.969 [0.875 - 0.993]
-4	0.683 [0.117 - 0.972]	0.799 [0.311 - 0.972]	0.952 [0.586 - 0.996]	0.825 [0.528 - 0.952]	0.842 [0.638 - 0.941]	0.960 [0.870 - 0.989]
-3.5	0.660 [0.129 - 0.962]	0.774 [0.321 - 0.961]	0.941 [0.600 - 0.994]	0.798 [0.517 - 0.936]	0.826 [0.649 - 0.924]	0.950 [0.862 - 0.983]
-3	0.636 [0.142 - 0.949]	0.747 [0.331 - 0.946]	0.929 [0.612 - 0.991]	0.768 [0.507 - 0.914]	0.808 [0.656 - 0.903]	0.937 [0.853 - 0.974]
-2.5	0.612 [0.156 - 0.931]	0.718 [0.340 - 0.927]	0.914 [0.622 - 0.986]	0.734 [0.496 - 0.886]	0.789 [0.660 - 0.878]	0.921 [0.841 - 0.962]
-2	0.587 [0.171 - 0.907]	0.688 [0.349 - 0.900]	0.897 [0.628 - 0.978]	0.698 [0.484 - 0.851]	0.768 [0.660 - 0.850]	0.900 [0.825 - 0.946]
-1.5	0.561 [0.187 - 0.877]	0.655 [0.357 - 0.867]	0.877 [0.630 - 0.967]	0.660 [0.472 - 0.808]	0.746 [0.654 - 0.820]	0.875 [0.804 - 0.923]
-1	0.535 [0.203 - 0.839]	0.621 [0.364 - 0.825]	0.853 [0.626 - 0.952]	0.619 [0.457 - 0.758]	0.720 [0.643 - 0.786]	0.845 [0.778 - 0.895]
-0.5	0.509 [0.219 - 0.793]	0.586 [0.368 - 0.776]	0.825 [0.618 - 0.932]	0.577 [0.441 - 0.702]	0.691 [0.627 - 0.749]	0.810 [0.747 - 0.861]
Mean	0.483 [0.235 - 0.740]	0.551 [0.368 - 0.721]	0.794 [0.606 - 0.906]	0.534 [0.422 - 0.643]	0.658 [0.605 - 0.708]	0.770 [0.711 - 0.820]
0.5	0.457 [0.248 - 0.683]	0.515 [0.364 - 0.663]	0.759 [0.588 - 0.874]	0.492 [0.400 - 0.584]	0.621 [0.576 - 0.664]	0.726 [0.671 - 0.775]
1	0.431 [0.257 - 0.623]	0.479 [0.355 - 0.606]	0.721 [0.567 - 0.836]	0.451 [0.377 - 0.527]	0.578 [0.540 - 0.616]	0.680 [0.630 - 0.727]
1.5	0.404 [0.261 - 0.566]	0.445 [0.343 - 0.551]	0.681 [0.541 - 0.794]	0.412 [0.353 - 0.473]	0.531 [0.498 - 0.564]	0.634 [0.588 - 0.678]
2	0.377 [0.260 - 0.511]	0.412 [0.329 - 0.500]	0.639 [0.513 - 0.748]	0.375 [0.328 - 0.424]	0.482 [0.453 - 0.510]	0.590 [0.549 - 0.630]
2.5	0.350 [0.254 - 0.460]	0.381 [0.313 - 0.455]	0.596 [0.482 - 0.701]	0.342 [0.304 - 0.381]	0.432 [0.407 - 0.456]	0.548 [0.511 - 0.585]
3	0.323 [0.243 - 0.413]	0.353 [0.295 - 0.416]	0.553 [0.449 - 0.653]	0.311 [0.280 - 0.345]	0.385 [0.364 - 0.406]	0.511 [0.477 - 0.545]
3.5	0.296 [0.230 - 0.372]	0.328 [0.277 - 0.382]	0.511 [0.414 - 0.606]	0.285 [0.259 - 0.313]	0.343 [0.325 - 0.362]	0.479 [0.448 - 0.510]
4	0.271 [0.215 - 0.336]	0.304 [0.260 - 0.353]	0.470 [0.380 - 0.561]	0.263 [0.240 - 0.288]	0.310 [0.293 - 0.326]	0.451 [0.422 - 0.481]
4.5	0.247 [0.198 - 0.304]	0.283 [0.243 - 0.327]	0.432 [0.349 - 0.519]	0.246 [0.225 - 0.268]	0.284 [0.270 - 0.299]	0.430 [0.402 - 0.458]
5	0.226 [0.181 - 0.277]	0.264 [0.227 - 0.305]	0.399 [0.321 - 0.482]	0.233 [0.213 - 0.254]	0.266 [0.252 - 0.280]	0.414 [0.386 - 0.442]
Age		30-34 yrs			>34 yrs	
Weight gain	Normal weight (n=12,302)	Underweight (n=2,460)	Overweight/Obese (n=4,749)	Normal weight (n=5,750)	Underweight (n=704)	Overweight/Obese (n=3,221)
-5	0.978 [0.772 - 0.998]	0.956 [0.829 - 0.990]	0.974 [0.897 - 0.994]	0.975 [0.613 - 0.999]	0.946 [0.800 - 0.987]	0.930 [0.816 - 0.975]
-4.5	0.971 [0.765 - 0.997]	0.949 [0.835 - 0.986]	0.968 [0.895 - 0.991]	0.968 [0.620 - 0.998]	0.940 [0.812 - 0.983]	0.924 [0.825 - 0.969]
-4	0.962 [0.758 - 0.995]	0.941 [0.839 - 0.980]	0.960 [0.892 - 0.986]	0.959 [0.627 - 0.997]	0.933 [0.819 - 0.977]	0.918 [0.832 - 0.962]
-3.5	0.950 [0.750 - 0.992]	0.932 [0.841 - 0.972]	0.951 [0.887 - 0.980]	0.948 [0.632 - 0.995]	0.924 [0.824 - 0.969]	0.912 [0.836 - 0.954]
-3	0.934 [0.741 - 0.986]	0.920 [0.840 - 0.962]	0.941 [0.881 - 0.971]	0.934 [0.637 - 0.991]	0.914 [0.825 - 0.960]	0.904 [0.838 - 0.945]
-2.5	0.915 [0.729 - 0.977]	0.907 [0.836 - 0.949]	0.928 [0.872 - 0.960]	0.916 [0.640 - 0.985]	0.902 [0.822 - 0.948]	0.895 [0.838 - 0.934]
-2	0.890 [0.714 - 0.963]	0.891 [0.828 - 0.932]	0.912 [0.860 - 0.946]	0.894 [0.640 - 0.976]	0.887 [0.816 - 0.932]	0.885 [0.834 - 0.921]
-1.5	0.859 [0.695 - 0.942]	0.871 [0.816 - 0.912]	0.893 [0.845 - 0.927]	0.867 [0.637 - 0.961]	0.868 [0.806 - 0.913]	0.872 [0.827 - 0.907]
-1	0.821 [0.671 - 0.912]	0.846 [0.797 - 0.885]	0.870 [0.826 - 0.904]	0.835 [0.627 - 0.938]	0.846 [0.791 - 0.889]	0.857 [0.816 - 0.891]
-0.5	0.776 [0.642 - 0.870]	0.816 [0.773 - 0.853]	0.842 [0.801 - 0.876]	0.797 [0.612 - 0.907]	0.820 [0.771 - 0.861]	0.839 [0.801 - 0.871]
Mean	0.725 [0.609 - 0.817]	0.779 [0.742 - 0.813]	0.807 [0.769 - 0.841]	0.754 [0.592 - 0.866]	0.789 [0.745 - 0.827]	0.816 [0.781 - 0.847]
0.5	0.669 [0.571 - 0.754]	0.735 [0.702 - 0.767]	0.767 [0.730 - 0.799]	0.705 [0.566 - 0.815]	0.753 [0.714 - 0.789]	0.790 [0.756 - 0.820]
1	0.611 [0.531 - 0.685]	0.684 [0.653 - 0.714]	0.720 [0.686 - 0.752]	0.655 [0.536 - 0.757]	0.712 [0.676 - 0.745]	0.759 [0.726 - 0.789]
1.5	0.554 [0.488 - 0.618]	0.628 [0.600 - 0.655]	0.670 [0.639 - 0.701]	0.604 [0.504 - 0.696]	0.665 [0.633 - 0.696]	0.724 [0.692 - 0.754]
2	0.501 [0.446 - 0.556]	0.568 [0.543 - 0.593]	0.621 [0.591 - 0.650]	0.555 [0.469 - 0.637]	0.615 [0.586 - 0.644]	0.687 [0.656 - 0.716]
2.5	0.454 [0.407 - 0.501]	0.509 [0.487 - 0.531]	0.575 [0.547 - 0.603]	0.508 [0.434 - 0.581]	0.563 [0.536 - 0.590]	0.650 [0.619 - 0.679]
3	0.413 [0.372 - 0.454]	0.454 [0.435 - 0.473]	0.535 [0.508 - 0.561]	0.463 [0.398 - 0.529]	0.512 [0.486 - 0.537]	0.614 [0.584 - 0.643]
3.5	0.377 [0.341 - 0.414]	0.405 [0.388 - 0.423]	0.501 [0.475 - 0.526]	0.422 [0.364 - 0.482]	0.465 [0.441 - 0.489]	0.581 [0.552 - 0.609]
4	0.346 [0.314 - 0.380]	0.365 [0.350 - 0.381]	0.472 [0.447 - 0.496]	0.386 [0.332 - 0.442]	0.425 [0.403 - 0.448]	0.552 [0.523 - 0.580]
4.5	0.319 [0.290 - 0.349]	0.334 [0.320 - 0.349]	0.447 [0.423 - 0.472]	0.356 [0.306 - 0.409]	0.394 [0.373 - 0.416]	0.527 [0.498 - 0.555]
5	0.295 [0.268 - 0.324]	0.312 [0.298 - 0.326]	0.428 [0.404 - 0.452]	0.334 [0.286 - 0.385]	0.370 [0.350 - 0.391]	0.506 [0.477 - 0.535]

3. Evaluation of weight gain between pre and 34 weeks after pregnancy

- Generalized additive model (GAM) was used for predictive model of each disease.
- The above statistical model was applied to subsets of each age and BMI (ASIA).
- Reference weight gain = 5kg (Mean weight gain of mothers without any disease).

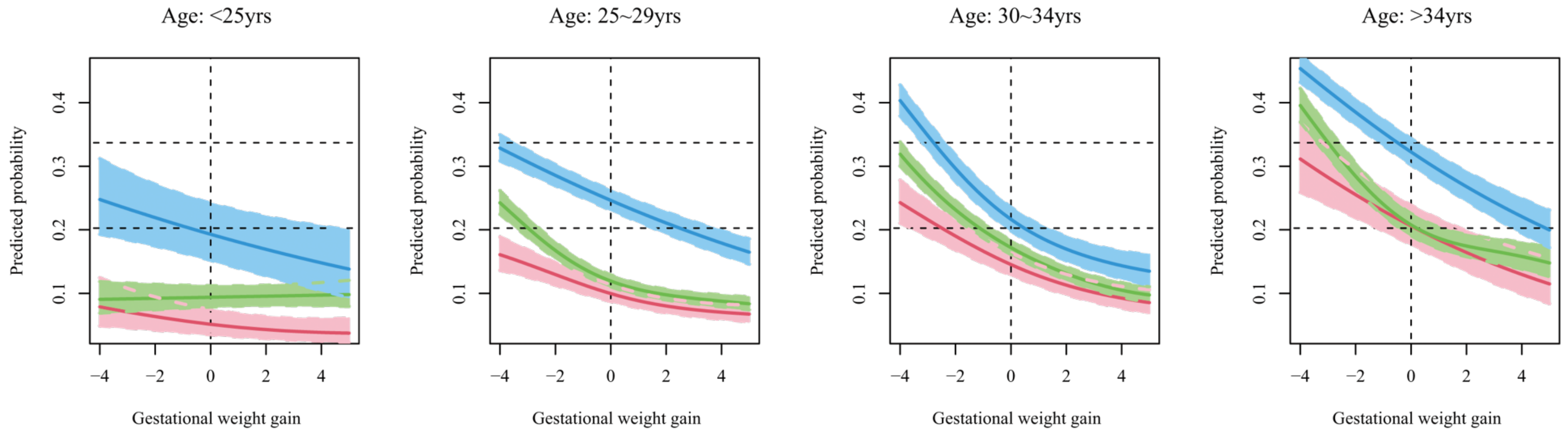

(a) **Gestational diabetes mellitus** (Red: Normal weight, Green: Underweight, Blue: Overweight/obese)

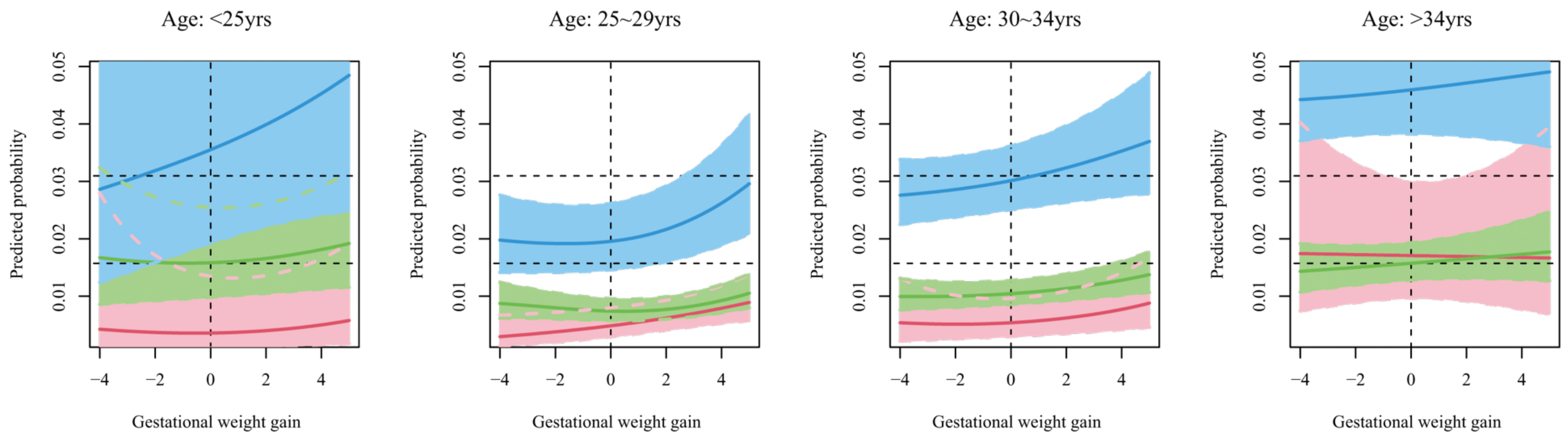

(b) **Preeclampsia** (Red: Normal weight, Green: Underweight, Blue: Overweight/obese)

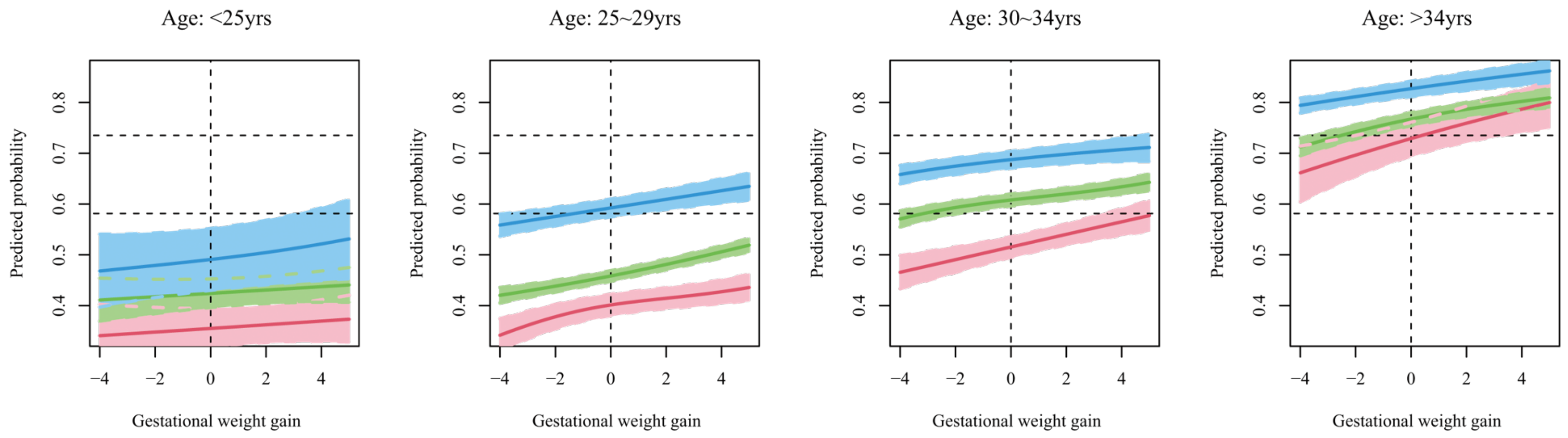

(c) **Cesarean delivery** (Red: Normal weight, Green: Underweight, Blue: Overweight/obese)

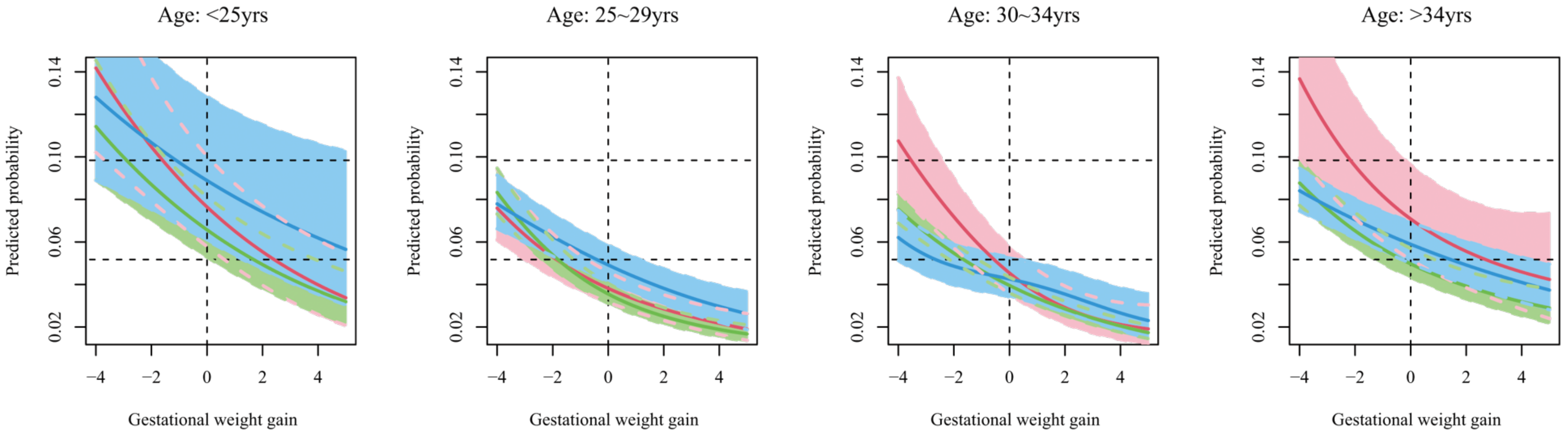

(d) **Preterm delivery** (Red: Normal weight, Green: Underweight, Blue: Overweight/obese)

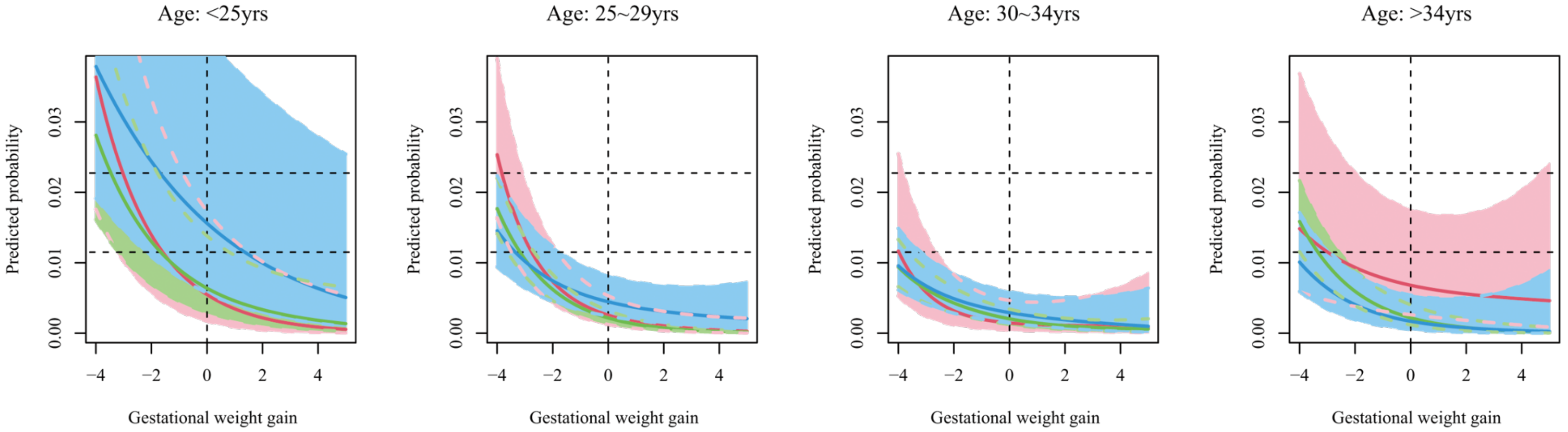

(e) **Stillbirth** (Red: Normal weight, Green: Underweight, Blue: Overweight/obese)

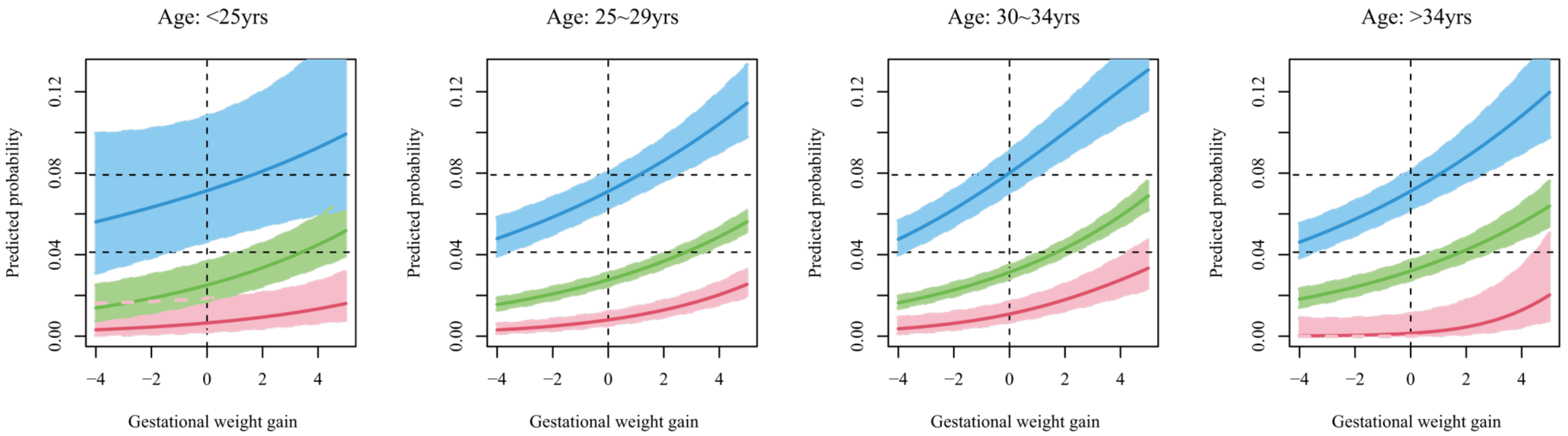

(f) **Macrosomia** (Red: Normal weight, Green: Underweight, Blue: Overweight/obese)

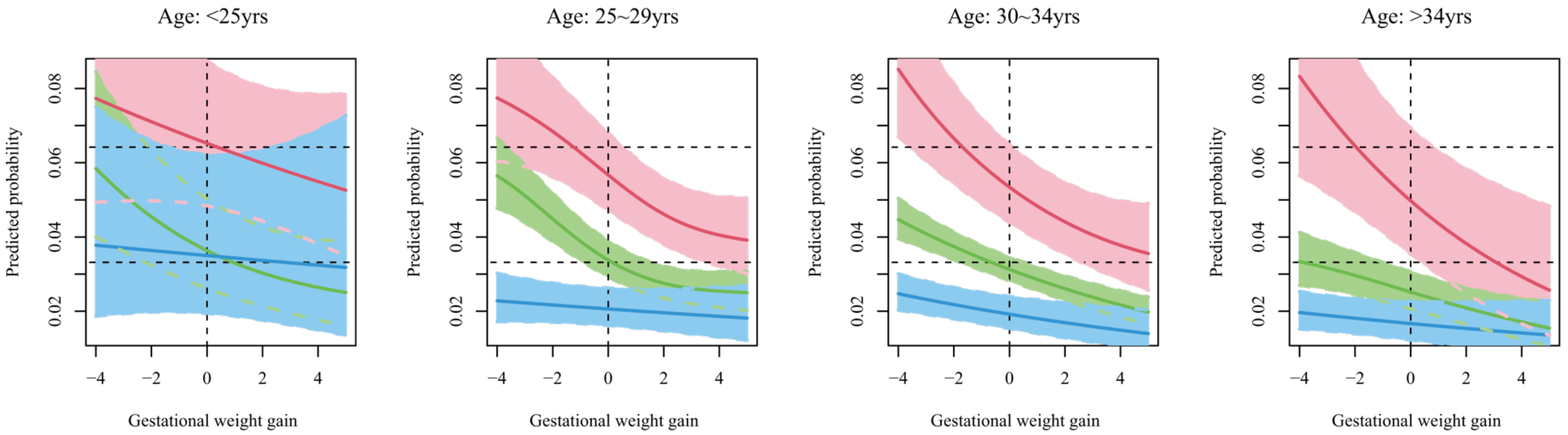

(g) **Small for gestational age** (Red: Normal weight, Green: Underweight, Blue: Overweight/obese)

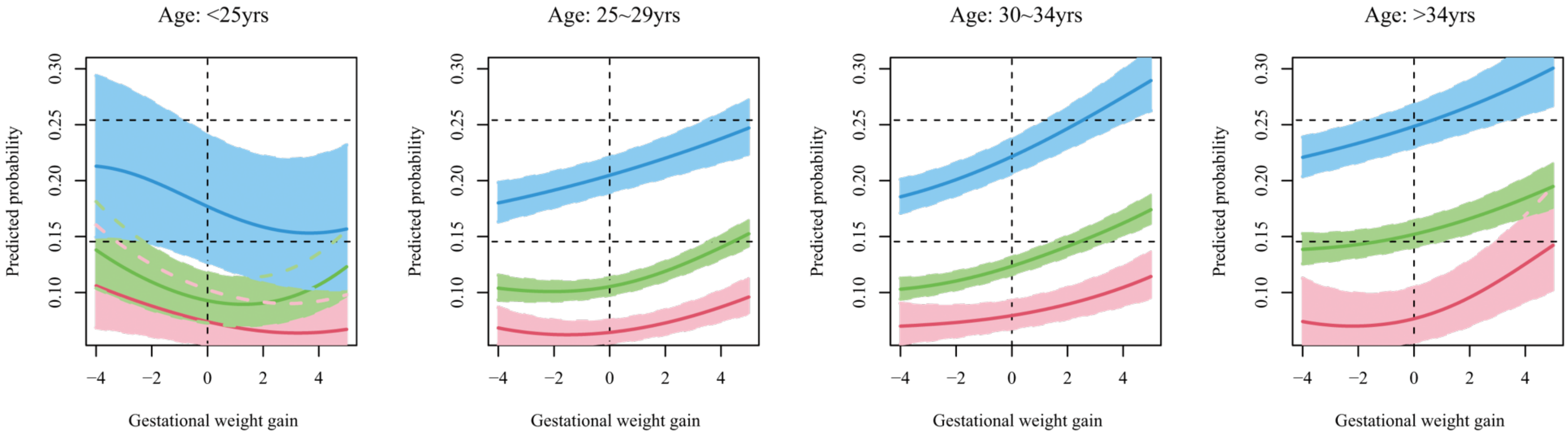

(h) **Large for gestational age** (Red: Normal weight, Green: Underweight, Blue: Overweight/obese)

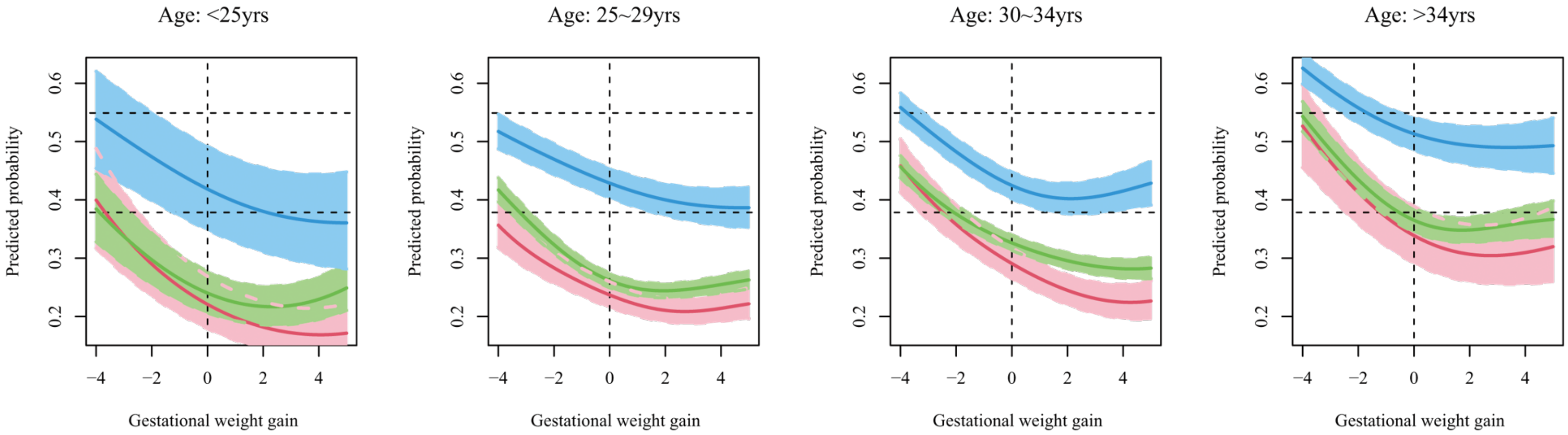

(i) **All disease** (Red: Normal weight, Green: Underweight, Blue: Overweight/obese)

3.1

Gestational diabetes mellitus

Age		<25 yrs			25-29 yrs	
Weight gain	Normal weight (n=1,324)	Underweight (n=698)	Overweight/Obese (n=314)	Normal weight (n=12,406)	Underweight (n=3,943)	Overweight/Obese (n=3,249)
-5	0.086 [0.051 - 0.141]	0.090 [0.066 - 0.121]	0.263 [0.201 - 0.336]	0.174 [0.144 - 0.209]	0.279 [0.256 - 0.304]	0.351 [0.328 - 0.375]
-4.5	0.082 [0.050 - 0.131]	0.090 [0.067 - 0.120]	0.255 [0.197 - 0.324]	0.167 [0.141 - 0.198]	0.262 [0.241 - 0.283]	0.340 [0.318 - 0.362]
-4	0.078 [0.049 - 0.122]	0.091 [0.069 - 0.118]	0.248 [0.193 - 0.312]	0.160 [0.136 - 0.188]	0.242 [0.224 - 0.262]	0.328 [0.308 - 0.349]
-3.5	0.074 [0.047 - 0.114]	0.091 [0.070 - 0.117]	0.240 [0.189 - 0.301]	0.153 [0.131 - 0.178]	0.223 [0.206 - 0.240]	0.317 [0.299 - 0.337]
-3	0.070 [0.046 - 0.106]	0.091 [0.072 - 0.116]	0.233 [0.184 - 0.290]	0.145 [0.125 - 0.168]	0.203 [0.188 - 0.219]	0.307 [0.289 - 0.325]
-2.5	0.067 [0.044 - 0.099]	0.092 [0.073 - 0.115]	0.226 [0.180 - 0.280]	0.137 [0.119 - 0.158]	0.185 [0.171 - 0.199]	0.296 [0.279 - 0.313]
-2	0.064 [0.043 - 0.093]	0.092 [0.074 - 0.114]	0.219 [0.174 - 0.271]	0.129 [0.113 - 0.148]	0.168 [0.155 - 0.181]	0.286 [0.270 - 0.302]
-1.5	0.060 [0.041 - 0.088]	0.093 [0.075 - 0.113]	0.212 [0.169 - 0.263]	0.121 [0.106 - 0.138]	0.152 [0.141 - 0.164]	0.275 [0.260 - 0.292]
-1	0.057 [0.039 - 0.083]	0.093 [0.076 - 0.113]	0.206 [0.164 - 0.255]	0.114 [0.100 - 0.129]	0.139 [0.129 - 0.150]	0.265 [0.250 - 0.281]
-0.5	0.055 [0.038 - 0.079]	0.093 [0.078 - 0.112]	0.199 [0.158 - 0.248]	0.106 [0.093 - 0.121]	0.128 [0.119 - 0.138]	0.256 [0.241 - 0.272]
Mean	0.052 [0.036 - 0.075]	0.094 [0.078 - 0.112]	0.193 [0.152 - 0.242]	0.100 [0.087 - 0.114]	0.119 [0.110 - 0.129]	0.246 [0.231 - 0.262]
0.5	0.050 [0.035 - 0.072]	0.094 [0.079 - 0.112]	0.187 [0.146 - 0.236]	0.094 [0.082 - 0.107]	0.112 [0.103 - 0.122]	0.237 [0.222 - 0.253]
1	0.048 [0.033 - 0.069]	0.095 [0.080 - 0.112]	0.181 [0.140 - 0.231]	0.089 [0.078 - 0.101]	0.106 [0.098 - 0.115]	0.228 [0.212 - 0.245]
1.5	0.046 [0.032 - 0.066]	0.095 [0.080 - 0.112]	0.175 [0.134 - 0.226]	0.084 [0.073 - 0.096]	0.101 [0.093 - 0.110]	0.219 [0.203 - 0.236]
2	0.044 [0.030 - 0.064]	0.096 [0.081 - 0.113]	0.169 [0.128 - 0.221]	0.080 [0.070 - 0.092]	0.098 [0.090 - 0.107]	0.211 [0.194 - 0.228]
2.5	0.043 [0.029 - 0.063]	0.096 [0.081 - 0.113]	0.164 [0.122 - 0.217]	0.077 [0.067 - 0.089]	0.095 [0.086 - 0.104]	0.203 [0.186 - 0.221]
3	0.041 [0.028 - 0.062]	0.096 [0.081 - 0.114]	0.158 [0.116 - 0.213]	0.075 [0.064 - 0.087]	0.092 [0.084 - 0.101]	0.195 [0.177 - 0.213]
3.5	0.040 [0.026 - 0.061]	0.097 [0.081 - 0.116]	0.153 [0.110 - 0.209]	0.072 [0.062 - 0.085]	0.090 [0.081 - 0.099]	0.187 [0.169 - 0.206]
4	0.039 [0.025 - 0.060]	0.097 [0.080 - 0.117]	0.148 [0.104 - 0.206]	0.070 [0.059 - 0.083]	0.088 [0.079 - 0.098]	0.179 [0.161 - 0.199]
4.5	0.038 [0.024 - 0.061]	0.098 [0.080 - 0.119]	0.143 [0.099 - 0.203]	0.069 [0.057 - 0.082]	0.086 [0.076 - 0.096]	0.172 [0.154 - 0.192]
5	0.038 [0.023 - 0.061]	0.098 [0.079 - 0.121]	0.138 [0.093 - 0.200]	0.067 [0.056 - 0.082]	0.084 [0.074 - 0.094]	0.165 [0.146 - 0.186]
Age		30-34 yrs			>34 yrs	
Weight gain	Normal weight (n=12,302)	Underweight (n=2,460)	Overweight/Obese (n=4,749)	Normal weight (n=5,750)	Underweight (n=704)	Overweight/Obese (n=3,221)
-5	0.269 [0.230 - 0.313]	0.371 [0.348 - 0.395]	0.456 [0.429 - 0.483]	0.342 [0.279 - 0.411]	0.453 [0.422 - 0.484]	0.491 [0.467 - 0.515]
-4.5	0.256 [0.220 - 0.295]	0.345 [0.324 - 0.366]	0.430 [0.405 - 0.456]	0.327 [0.269 - 0.390]	0.425 [0.396 - 0.454]	0.473 [0.449 - 0.496]
-4	0.242 [0.210 - 0.277]	0.319 [0.300 - 0.338]	0.403 [0.379 - 0.428]	0.311 [0.259 - 0.369]	0.396 [0.369 - 0.422]	0.454 [0.432 - 0.477]
-3.5	0.229 [0.200 - 0.260]	0.294 [0.277 - 0.312]	0.376 [0.353 - 0.399]	0.297 [0.249 - 0.349]	0.366 [0.342 - 0.391]	0.436 [0.414 - 0.458]
-3	0.215 [0.189 - 0.244]	0.271 [0.256 - 0.287]	0.348 [0.326 - 0.371]	0.282 [0.239 - 0.330]	0.337 [0.315 - 0.361]	0.418 [0.396 - 0.440]
-2.5	0.202 [0.179 - 0.228]	0.250 [0.236 - 0.266]	0.322 [0.301 - 0.343]	0.269 [0.229 - 0.312]	0.310 [0.289 - 0.332]	0.400 [0.379 - 0.422]
-2	0.190 [0.168 - 0.214]	0.231 [0.218 - 0.246]	0.296 [0.276 - 0.318]	0.255 [0.219 - 0.295]	0.284 [0.264 - 0.305]	0.383 [0.362 - 0.405]
-1.5	0.178 [0.158 - 0.200]	0.214 [0.202 - 0.228]	0.273 [0.253 - 0.294]	0.242 [0.209 - 0.279]	0.261 [0.242 - 0.281]	0.367 [0.346 - 0.388]
-1	0.166 [0.148 - 0.187]	0.199 [0.187 - 0.211]	0.252 [0.233 - 0.272]	0.230 [0.198 - 0.264]	0.240 [0.222 - 0.259]	0.351 [0.330 - 0.372]
-0.5	0.156 [0.138 - 0.175]	0.185 [0.174 - 0.197]	0.233 [0.215 - 0.253]	0.218 [0.188 - 0.251]	0.222 [0.205 - 0.241]	0.335 [0.314 - 0.357]
Mean	0.146 [0.129 - 0.164]	0.172 [0.161 - 0.184]	0.217 [0.199 - 0.236]	0.206 [0.177 - 0.239]	0.208 [0.191 - 0.226]	0.320 [0.299 - 0.342]
0.5	0.136 [0.120 - 0.154]	0.160 [0.150 - 0.172]	0.202 [0.184 - 0.221]	0.195 [0.166 - 0.227]	0.196 [0.179 - 0.214]	0.306 [0.285 - 0.329]
1	0.128 [0.112 - 0.145]	0.150 [0.139 - 0.161]	0.190 [0.172 - 0.209]	0.184 [0.156 - 0.217]	0.187 [0.170 - 0.205]	0.293 [0.271 - 0.316]
1.5	0.120 [0.105 - 0.137]	0.140 [0.130 - 0.151]	0.179 [0.161 - 0.199]	0.174 [0.145 - 0.207]	0.180 [0.163 - 0.198]	0.280 [0.257 - 0.303]
2	0.113 [0.098 - 0.130]	0.131 [0.121 - 0.142]	0.170 [0.151 - 0.190]	0.164 [0.135 - 0.199]	0.174 [0.158 - 0.193]	0.267 [0.244 - 0.292]
2.5	0.107 [0.092 - 0.124]	0.123 [0.113 - 0.134]	0.162 [0.143 - 0.182]	0.155 [0.125 - 0.190]	0.170 [0.153 - 0.189]	0.255 [0.231 - 0.281]
3	0.102 [0.087 - 0.119]	0.116 [0.106 - 0.127]	0.155 [0.136 - 0.176]	0.146 [0.116 - 0.182]	0.166 [0.148 - 0.186]	0.244 [0.219 - 0.271]
3.5	0.097 [0.082 - 0.115]	0.110 [0.100 - 0.122]	0.149 [0.129 - 0.171]	0.138 [0.107 - 0.175]	0.162 [0.143 - 0.184]	0.233 [0.207 - 0.261]
4	0.093 [0.077 - 0.111]	0.105 [0.094 - 0.117]	0.144 [0.123 - 0.166]	0.130 [0.099 - 0.168]	0.158 [0.138 - 0.180]	0.222 [0.195 - 0.252]
4.5	0.089 [0.073 - 0.108]	0.101 [0.090 - 0.113]	0.139 [0.118 - 0.163]	0.122 [0.091 - 0.161]	0.153 [0.132 - 0.176]	0.212 [0.183 - 0.243]
5	0.085 [0.069 - 0.106]	0.097 [0.086 - 0.110]	0.135 [0.113 - 0.161]	0.115 [0.084 - 0.155]	0.148 [0.126 - 0.173]	0.202 [0.172 - 0.235]

3.2

Preeclampsia

Age		<25 yrs			25-29 yrs	
Weight gain	Normal weight (n=1,324)	Underweight (n=698)	Overweight/Obese (n=314)	Normal weight (n=12,406)	Underweight (n=3,943)	Overweight/Obese (n=3,249)
-5	0.003 [0.000 - 0.032]	0.017 [0.008 - 0.035]	0.026 [0.010 - 0.065]	0.002 [0.001 - 0.007]	0.009 [0.006 - 0.014]	0.020 [0.014 - 0.029]
-4.5	0.003 [0.000 - 0.028]	0.017 [0.008 - 0.033]	0.027 [0.011 - 0.064]	0.002 [0.001 - 0.007]	0.009 [0.006 - 0.013]	0.020 [0.014 - 0.028]
-4	0.003 [0.000 - 0.025]	0.016 [0.008 - 0.032]	0.028 [0.012 - 0.064]	0.002 [0.001 - 0.007]	0.009 [0.006 - 0.012]	0.020 [0.014 - 0.027]
-3.5	0.003 [0.000 - 0.022]	0.016 [0.009 - 0.031]	0.029 [0.013 - 0.063]	0.003 [0.001 - 0.007]	0.009 [0.006 - 0.012]	0.020 [0.014 - 0.027]
-3	0.003 [0.001 - 0.020]	0.016 [0.009 - 0.029]	0.030 [0.014 - 0.063]	0.003 [0.001 - 0.007]	0.008 [0.006 - 0.011]	0.019 [0.014 - 0.026]
-2.5	0.003 [0.001 - 0.019]	0.016 [0.009 - 0.028]	0.031 [0.015 - 0.063]	0.003 [0.001 - 0.007]	0.008 [0.006 - 0.011]	0.019 [0.014 - 0.026]
-2	0.003 [0.001 - 0.017]	0.016 [0.009 - 0.028]	0.032 [0.016 - 0.063]	0.004 [0.002 - 0.007]	0.008 [0.006 - 0.011]	0.019 [0.014 - 0.026]
-1.5	0.004 [0.001 - 0.016]	0.016 [0.010 - 0.027]	0.033 [0.017 - 0.063]	0.004 [0.002 - 0.007]	0.008 [0.006 - 0.010]	0.019 [0.014 - 0.026]
-1	0.004 [0.001 - 0.015]	0.016 [0.010 - 0.026]	0.034 [0.018 - 0.063]	0.004 [0.002 - 0.008]	0.008 [0.006 - 0.010]	0.019 [0.014 - 0.026]
-0.5	0.004 [0.001 - 0.014]	0.016 [0.010 - 0.026]	0.035 [0.019 - 0.064]	0.005 [0.003 - 0.008]	0.007 [0.006 - 0.010]	0.019 [0.015 - 0.026]
Mean	0.004 [0.001 - 0.014]	0.016 [0.010 - 0.026]	0.036 [0.020 - 0.065]	0.005 [0.003 - 0.008]	0.007 [0.006 - 0.010]	0.020 [0.015 - 0.026]
0.5	0.004 [0.001 - 0.014]	0.017 [0.011 - 0.026]	0.037 [0.021 - 0.066]	0.005 [0.003 - 0.009]	0.007 [0.006 - 0.009]	0.020 [0.015 - 0.027]
1	0.004 [0.001 - 0.014]	0.017 [0.011 - 0.026]	0.039 [0.022 - 0.067]	0.006 [0.004 - 0.009]	0.007 [0.006 - 0.010]	0.020 [0.015 - 0.027]
1.5	0.004 [0.001 - 0.014]	0.017 [0.011 - 0.026]	0.040 [0.023 - 0.069]	0.006 [0.004 - 0.010]	0.008 [0.006 - 0.010]	0.021 [0.016 - 0.028]
2	0.005 [0.001 - 0.014]	0.017 [0.011 - 0.026]	0.041 [0.023 - 0.071]	0.007 [0.004 - 0.010]	0.008 [0.006 - 0.010]	0.022 [0.016 - 0.029]
2.5	0.005 [0.002 - 0.014]	0.017 [0.011 - 0.027]	0.042 [0.024 - 0.074]	0.007 [0.005 - 0.011]	0.008 [0.006 - 0.010]	0.023 [0.017 - 0.031]
3	0.005 [0.002 - 0.015]	0.018 [0.011 - 0.028]	0.044 [0.025 - 0.077]	0.007 [0.005 - 0.011]	0.008 [0.006 - 0.011]	0.024 [0.017 - 0.032]
3.5	0.005 [0.002 - 0.016]	0.018 [0.012 - 0.028]	0.045 [0.025 - 0.080]	0.008 [0.005 - 0.012]	0.009 [0.007 - 0.011]	0.025 [0.018 - 0.034]
4	0.006 [0.002 - 0.017]	0.018 [0.012 - 0.029]	0.047 [0.025 - 0.084]	0.008 [0.005 - 0.013]	0.009 [0.007 - 0.012]	0.026 [0.019 - 0.036]
4.5	0.006 [0.002 - 0.018]	0.019 [0.012 - 0.030]	0.048 [0.025 - 0.089]	0.009 [0.006 - 0.014]	0.010 [0.007 - 0.013]	0.028 [0.020 - 0.039]
5	0.007 [0.002 - 0.020]	0.019 [0.012 - 0.032]	0.050 [0.026 - 0.094]	0.009 [0.006 - 0.015]	0.011 [0.008 - 0.014]	0.030 [0.021 - 0.042]
Age		30-34 yrs			>34 yrs	
Weight gain	Normal weight (n=12,302)	Underweight (n=2,460)	Overweight/Obese (n=4,749)	Normal weight (n=5,750)	Underweight (n=704)	Overweight/Obese (n=3,221)
-5	0.005 [0.002 - 0.014]	0.010 [0.007 - 0.014]	0.027 [0.022 - 0.034]	0.018 [0.007 - 0.045]	0.014 [0.010 - 0.019]	0.044 [0.037 - 0.053]
-4.5	0.005 [0.002 - 0.013]	0.010 [0.007 - 0.013]	0.027 [0.022 - 0.034]	0.017 [0.007 - 0.043]	0.014 [0.010 - 0.019]	0.044 [0.037 - 0.053]
-4	0.005 [0.002 - 0.012]	0.010 [0.008 - 0.013]	0.028 [0.023 - 0.034]	0.017 [0.007 - 0.040]	0.014 [0.011 - 0.019]	0.044 [0.037 - 0.053]
-3.5	0.005 [0.002 - 0.012]	0.010 [0.008 - 0.013]	0.028 [0.023 - 0.034]	0.017 [0.008 - 0.038]	0.015 [0.011 - 0.019]	0.045 [0.038 - 0.053]
-3	0.005 [0.002 - 0.011]	0.010 [0.008 - 0.013]	0.028 [0.023 - 0.034]	0.017 [0.008 - 0.036]	0.015 [0.011 - 0.019]	0.045 [0.038 - 0.053]
-2.5	0.005 [0.002 - 0.010]	0.010 [0.008 - 0.013]	0.028 [0.024 - 0.034]	0.017 [0.009 - 0.035]	0.015 [0.012 - 0.019]	0.045 [0.038 - 0.053]
-2	0.005 [0.003 - 0.010]	0.010 [0.008 - 0.012]	0.029 [0.024 - 0.034]	0.017 [0.009 - 0.033]	0.015 [0.012 - 0.019]	0.045 [0.038 - 0.053]
-1.5	0.005 [0.003 - 0.010]	0.010 [0.008 - 0.012]	0.029 [0.024 - 0.035]	0.017 [0.009 - 0.032]	0.015 [0.012 - 0.019]	0.045 [0.038 - 0.053]
-1	0.005 [0.003 - 0.010]	0.010 [0.008 - 0.013]	0.029 [0.025 - 0.035]	0.017 [0.009 - 0.031]	0.015 [0.012 - 0.019]	0.046 [0.039 - 0.054]
-0.5	0.005 [0.003 - 0.010]	0.010 [0.008 - 0.013]	0.030 [0.025 - 0.036]	0.017 [0.010 - 0.030]	0.016 [0.013 - 0.019]	0.046 [0.039 - 0.054]
Mean	0.006 [0.003 - 0.010]	0.011 [0.009 - 0.013]	0.030 [0.025 - 0.036]	0.017 [0.010 - 0.030]	0.016 [0.013 - 0.019]	0.046 [0.038 - 0.055]
0.5	0.006 [0.003 - 0.010]	0.011 [0.009 - 0.013]	0.031 [0.026 - 0.037]	0.017 [0.010 - 0.030]	0.016 [0.013 - 0.020]	0.046 [0.038 - 0.056]
1	0.006 [0.003 - 0.010]	0.011 [0.009 - 0.013]	0.031 [0.026 - 0.038]	0.017 [0.010 - 0.030]	0.016 [0.013 - 0.020]	0.047 [0.038 - 0.057]
1.5	0.006 [0.004 - 0.011]	0.011 [0.009 - 0.014]	0.032 [0.026 - 0.039]	0.017 [0.009 - 0.030]	0.016 [0.013 - 0.020]	0.047 [0.038 - 0.058]
2	0.007 [0.004 - 0.011]	0.011 [0.009 - 0.014]	0.032 [0.026 - 0.040]	0.017 [0.009 - 0.031]	0.017 [0.013 - 0.021]	0.047 [0.038 - 0.058]
2.5	0.007 [0.004 - 0.012]	0.012 [0.010 - 0.014]	0.033 [0.027 - 0.041]	0.017 [0.009 - 0.032]	0.017 [0.013 - 0.021]	0.047 [0.038 - 0.060]
3	0.007 [0.004 - 0.012]	0.012 [0.010 - 0.015]	0.034 [0.027 - 0.042]	0.017 [0.009 - 0.033]	0.017 [0.013 - 0.022]	0.048 [0.037 - 0.061]
3.5	0.008 [0.004 - 0.013]	0.012 [0.010 - 0.015]	0.034 [0.027 - 0.044]	0.017 [0.008 - 0.034]	0.017 [0.013 - 0.023]	0.048 [0.037 - 0.062]
4	0.008 [0.005 - 0.014]	0.013 [0.010 - 0.016]	0.035 [0.027 - 0.045]	0.017 [0.008 - 0.036]	0.017 [0.013 - 0.023]	0.048 [0.037 - 0.063]
4.5	0.008 [0.005 - 0.015]	0.013 [0.010 - 0.017]	0.036 [0.028 - 0.047]	0.017 [0.007 - 0.038]	0.018 [0.013 - 0.024]	0.048 [0.036 - 0.064]
5	0.009 [0.005 - 0.017]	0.014 [0.011 - 0.018]	0.037 [0.028 - 0.048]	0.017 [0.007 - 0.040]	0.018 [0.013 - 0.025]	0.049 [0.036 - 0.066]

3.3

Cesarean delivery

Age		<25 yrs			25-29 yrs	
Weight gain	Normal weight (n=1,324)	Underweight (n=698)	Overweight/Obese (n=314)	Normal weight (n=12,406)	Underweight (n=3,943)	Overweight/Obese (n=3,249)
-5	0.337 [0.275 - 0.406]	0.408 [0.362 - 0.456]	0.462 [0.385 - 0.540]	0.320 [0.283 - 0.359]	0.412 [0.394 - 0.430]	0.550 [0.526 - 0.574]
-4.5	0.339 [0.279 - 0.404]	0.409 [0.365 - 0.455]	0.465 [0.391 - 0.540]	0.331 [0.297 - 0.367]	0.416 [0.400 - 0.433]	0.554 [0.531 - 0.577]
-4	0.341 [0.284 - 0.402]	0.411 [0.369 - 0.454]	0.468 [0.397 - 0.539]	0.342 [0.310 - 0.374]	0.420 [0.405 - 0.436]	0.559 [0.537 - 0.580]
-3.5	0.342 [0.289 - 0.401]	0.413 [0.373 - 0.453]	0.471 [0.403 - 0.540]	0.352 [0.323 - 0.382]	0.425 [0.410 - 0.439]	0.563 [0.542 - 0.583]
-3	0.344 [0.293 - 0.399]	0.414 [0.377 - 0.453]	0.473 [0.408 - 0.540]	0.361 [0.334 - 0.389]	0.429 [0.416 - 0.442]	0.567 [0.548 - 0.587]
-2.5	0.346 [0.298 - 0.398]	0.416 [0.380 - 0.452]	0.476 [0.413 - 0.541]	0.370 [0.345 - 0.396]	0.434 [0.421 - 0.446]	0.571 [0.553 - 0.590]
-2	0.348 [0.302 - 0.396]	0.417 [0.384 - 0.452]	0.479 [0.417 - 0.542]	0.378 [0.354 - 0.403]	0.438 [0.426 - 0.450]	0.576 [0.558 - 0.594]
-1.5	0.350 [0.306 - 0.395]	0.419 [0.387 - 0.452]	0.482 [0.421 - 0.544]	0.385 [0.363 - 0.409]	0.443 [0.432 - 0.454]	0.580 [0.562 - 0.597]
-1	0.351 [0.310 - 0.395]	0.421 [0.390 - 0.452]	0.486 [0.425 - 0.546]	0.392 [0.370 - 0.414]	0.448 [0.437 - 0.459]	0.584 [0.567 - 0.601]
-0.5	0.353 [0.314 - 0.394]	0.422 [0.393 - 0.452]	0.489 [0.429 - 0.549]	0.397 [0.376 - 0.419]	0.453 [0.442 - 0.464]	0.589 [0.571 - 0.606]
Mean	0.355 [0.318 - 0.394]	0.424 [0.396 - 0.452]	0.492 [0.432 - 0.552]	0.402 [0.381 - 0.423]	0.458 [0.448 - 0.469]	0.593 [0.575 - 0.610]
0.5	0.357 [0.321 - 0.395]	0.426 [0.399 - 0.453]	0.495 [0.435 - 0.555]	0.405 [0.385 - 0.426]	0.464 [0.453 - 0.474]	0.597 [0.579 - 0.614]
1	0.359 [0.323 - 0.395]	0.427 [0.401 - 0.454]	0.499 [0.438 - 0.559]	0.409 [0.388 - 0.429]	0.469 [0.459 - 0.480]	0.601 [0.583 - 0.619]
1.5	0.360 [0.326 - 0.397]	0.429 [0.403 - 0.456]	0.502 [0.441 - 0.564]	0.412 [0.391 - 0.432]	0.475 [0.464 - 0.486]	0.605 [0.587 - 0.624]
2	0.362 [0.327 - 0.399]	0.431 [0.404 - 0.458]	0.506 [0.443 - 0.568]	0.414 [0.394 - 0.435]	0.481 [0.470 - 0.492]	0.610 [0.590 - 0.629]
2.5	0.364 [0.328 - 0.401]	0.432 [0.405 - 0.460]	0.510 [0.445 - 0.574]	0.417 [0.396 - 0.438]	0.487 [0.476 - 0.498]	0.614 [0.593 - 0.634]
3	0.366 [0.329 - 0.404]	0.434 [0.406 - 0.462]	0.514 [0.448 - 0.580]	0.420 [0.398 - 0.442]	0.493 [0.482 - 0.504]	0.618 [0.596 - 0.639]
3.5	0.368 [0.329 - 0.408]	0.436 [0.406 - 0.465]	0.518 [0.449 - 0.586]	0.423 [0.401 - 0.446]	0.499 [0.488 - 0.511]	0.622 [0.599 - 0.644]
4	0.369 [0.329 - 0.411]	0.437 [0.407 - 0.468]	0.523 [0.451 - 0.593]	0.427 [0.404 - 0.451]	0.506 [0.494 - 0.518]	0.626 [0.602 - 0.650]
4.5	0.371 [0.329 - 0.416]	0.439 [0.407 - 0.471]	0.527 [0.453 - 0.601]	0.431 [0.406 - 0.456]	0.512 [0.500 - 0.525]	0.630 [0.605 - 0.655]
5	0.373 [0.328 - 0.420]	0.441 [0.407 - 0.475]	0.532 [0.454 - 0.609]	0.435 [0.409 - 0.462]	0.519 [0.505 - 0.533]	0.635 [0.608 - 0.661]
Age		30-34 yrs			>34 yrs	
Weight gain	Normal weight (n=12,302)	Underweight (n=2,460)	Overweight/Obese (n=4,749)	Normal weight (n=5,750)	Underweight (n=704)	Overweight/Obese (n=3,221)
-5	0.453 [0.417 - 0.490]	0.556 [0.537 - 0.574]	0.648 [0.628 - 0.667]	0.643 [0.577 - 0.705]	0.696 [0.676 - 0.716]	0.785 [0.769 - 0.801]
-4.5	0.459 [0.425 - 0.494]	0.564 [0.547 - 0.581]	0.653 [0.634 - 0.672]	0.653 [0.591 - 0.709]	0.705 [0.686 - 0.723]	0.790 [0.774 - 0.805]
-4	0.466 [0.433 - 0.498]	0.571 [0.555 - 0.587]	0.658 [0.640 - 0.676]	0.662 [0.605 - 0.714]	0.713 [0.695 - 0.730]	0.794 [0.779 - 0.809]
-3.5	0.472 [0.441 - 0.502]	0.577 [0.562 - 0.592]	0.663 [0.645 - 0.680]	0.670 [0.618 - 0.719]	0.720 [0.704 - 0.736]	0.799 [0.784 - 0.812]
-3	0.478 [0.450 - 0.507]	0.583 [0.569 - 0.597]	0.667 [0.650 - 0.684]	0.679 [0.631 - 0.724]	0.728 [0.712 - 0.743]	0.803 [0.789 - 0.816]
-2.5	0.484 [0.458 - 0.511]	0.589 [0.575 - 0.602]	0.671 [0.654 - 0.687]	0.688 [0.643 - 0.729]	0.735 [0.720 - 0.750]	0.807 [0.793 - 0.820]
-2	0.490 [0.466 - 0.515]	0.593 [0.580 - 0.606]	0.675 [0.658 - 0.691]	0.696 [0.655 - 0.735]	0.742 [0.728 - 0.756]	0.811 [0.797 - 0.825]
-1.5	0.497 [0.473 - 0.520]	0.598 [0.585 - 0.610]	0.678 [0.661 - 0.694]	0.705 [0.666 - 0.741]	0.749 [0.735 - 0.763]	0.815 [0.801 - 0.829]
-1	0.503 [0.481 - 0.525]	0.601 [0.589 - 0.613]	0.681 [0.665 - 0.698]	0.713 [0.676 - 0.747]	0.756 [0.742 - 0.769]	0.819 [0.805 - 0.833]
-0.5	0.509 [0.488 - 0.530]	0.605 [0.593 - 0.617]	0.684 [0.668 - 0.701]	0.721 [0.686 - 0.754]	0.762 [0.748 - 0.775]	0.823 [0.808 - 0.838]
Mean	0.515 [0.495 - 0.536]	0.608 [0.596 - 0.620]	0.687 [0.671 - 0.704]	0.729 [0.694 - 0.761]	0.767 [0.754 - 0.780]	0.827 [0.811 - 0.842]
0.5	0.522 [0.502 - 0.541]	0.611 [0.599 - 0.623]	0.690 [0.673 - 0.707]	0.737 [0.702 - 0.768]	0.773 [0.759 - 0.786]	0.831 [0.815 - 0.846]
1	0.528 [0.508 - 0.548]	0.614 [0.602 - 0.626]	0.693 [0.675 - 0.710]	0.744 [0.709 - 0.776]	0.778 [0.764 - 0.791]	0.835 [0.818 - 0.850]
1.5	0.534 [0.514 - 0.554]	0.617 [0.605 - 0.629]	0.696 [0.677 - 0.713]	0.752 [0.716 - 0.784]	0.782 [0.768 - 0.796]	0.838 [0.821 - 0.855]
2	0.540 [0.519 - 0.561]	0.620 [0.607 - 0.632]	0.698 [0.679 - 0.717]	0.759 [0.722 - 0.793]	0.787 [0.772 - 0.800]	0.842 [0.824 - 0.859]
2.5	0.546 [0.525 - 0.568]	0.623 [0.610 - 0.636]	0.701 [0.681 - 0.720]	0.766 [0.728 - 0.801]	0.791 [0.776 - 0.805]	0.845 [0.826 - 0.863]
3	0.553 [0.529 - 0.576]	0.626 [0.613 - 0.639]	0.703 [0.682 - 0.723]	0.773 [0.733 - 0.809]	0.795 [0.779 - 0.809]	0.849 [0.829 - 0.867]
3.5	0.559 [0.534 - 0.583]	0.630 [0.616 - 0.644]	0.705 [0.683 - 0.726]	0.780 [0.737 - 0.818]	0.799 [0.782 - 0.814]	0.852 [0.832 - 0.871]
4	0.565 [0.539 - 0.591]	0.634 [0.619 - 0.648]	0.707 [0.684 - 0.730]	0.787 [0.742 - 0.826]	0.802 [0.785 - 0.818]	0.856 [0.834 - 0.875]
4.5	0.571 [0.543 - 0.599]	0.638 [0.622 - 0.654]	0.709 [0.684 - 0.733]	0.794 [0.746 - 0.834]	0.806 [0.787 - 0.823]	0.859 [0.837 - 0.879]
5	0.577 [0.547 - 0.607]	0.643 [0.626 - 0.659]	0.711 [0.684 - 0.737]	0.800 [0.750 - 0.842]	0.809 [0.789 - 0.827]	0.862 [0.839 - 0.882]

3.4

Preterm delivery

Age		<25 yrs			25-29 yrs	
Weight gain	Normal weight (n=1,324)	Underweight (n=698)	Overweight/Obese (n=314)	Normal weight (n=12,406)	Underweight (n=3,943)	Overweight/Obese (n=3,249)
-5	0.164 [0.114 - 0.230]	0.130 [0.099 - 0.170]	0.140 [0.096 - 0.201]	0.091 [0.071 - 0.115]	0.101 [0.088 - 0.116]	0.087 [0.073 - 0.103]
-4.5	0.153 [0.108 - 0.211]	0.122 [0.094 - 0.157]	0.134 [0.093 - 0.191]	0.083 [0.066 - 0.104]	0.092 [0.081 - 0.105]	0.083 [0.070 - 0.098]
-4	0.142 [0.102 - 0.194]	0.114 [0.089 - 0.145]	0.129 [0.090 - 0.181]	0.076 [0.061 - 0.094]	0.083 [0.073 - 0.094]	0.079 [0.066 - 0.093]
-3.5	0.132 [0.096 - 0.177]	0.107 [0.084 - 0.134]	0.123 [0.087 - 0.172]	0.069 [0.056 - 0.085]	0.075 [0.066 - 0.085]	0.074 [0.063 - 0.088]
-3	0.122 [0.090 - 0.163]	0.100 [0.079 - 0.125]	0.118 [0.084 - 0.164]	0.063 [0.052 - 0.077]	0.067 [0.059 - 0.076]	0.071 [0.060 - 0.083]
-2.5	0.113 [0.085 - 0.149]	0.093 [0.075 - 0.115]	0.113 [0.080 - 0.156]	0.058 [0.048 - 0.070]	0.060 [0.053 - 0.068]	0.067 [0.057 - 0.078]
-2	0.105 [0.079 - 0.137]	0.087 [0.070 - 0.107]	0.108 [0.076 - 0.149]	0.053 [0.044 - 0.064]	0.054 [0.048 - 0.061]	0.063 [0.053 - 0.074]
-1.5	0.097 [0.074 - 0.126]	0.081 [0.066 - 0.100]	0.103 [0.073 - 0.143]	0.049 [0.041 - 0.059]	0.048 [0.043 - 0.055]	0.059 [0.050 - 0.070]
-1	0.090 [0.068 - 0.117]	0.076 [0.061 - 0.093]	0.098 [0.069 - 0.138]	0.045 [0.037 - 0.054]	0.044 [0.038 - 0.049]	0.056 [0.047 - 0.066]
-0.5	0.083 [0.063 - 0.108]	0.071 [0.057 - 0.087]	0.094 [0.065 - 0.133]	0.041 [0.035 - 0.050]	0.039 [0.035 - 0.045]	0.052 [0.044 - 0.062]
Mean	0.076 [0.058 - 0.100]	0.066 [0.053 - 0.081]	0.090 [0.062 - 0.129]	0.038 [0.032 - 0.046]	0.036 [0.031 - 0.041]	0.049 [0.041 - 0.059]
0.5	0.071 [0.053 - 0.094]	0.061 [0.049 - 0.076]	0.086 [0.058 - 0.125]	0.036 [0.029 - 0.043]	0.032 [0.028 - 0.037]	0.046 [0.038 - 0.056]
1	0.065 [0.048 - 0.087]	0.057 [0.045 - 0.072]	0.082 [0.054 - 0.121]	0.033 [0.027 - 0.040]	0.030 [0.026 - 0.034]	0.043 [0.035 - 0.052]
1.5	0.060 [0.044 - 0.082]	0.053 [0.042 - 0.068]	0.078 [0.051 - 0.118]	0.031 [0.025 - 0.037]	0.027 [0.023 - 0.031]	0.040 [0.033 - 0.050]
2	0.055 [0.040 - 0.077]	0.049 [0.038 - 0.064]	0.074 [0.047 - 0.115]	0.029 [0.023 - 0.035]	0.025 [0.022 - 0.029]	0.038 [0.030 - 0.047]
2.5	0.051 [0.036 - 0.072]	0.046 [0.035 - 0.060]	0.071 [0.044 - 0.113]	0.027 [0.021 - 0.033]	0.023 [0.020 - 0.027]	0.035 [0.028 - 0.045]
3	0.047 [0.032 - 0.068]	0.043 [0.032 - 0.057]	0.068 [0.041 - 0.110]	0.025 [0.020 - 0.031]	0.022 [0.018 - 0.026]	0.033 [0.026 - 0.043]
3.5	0.043 [0.029 - 0.064]	0.040 [0.029 - 0.054]	0.065 [0.038 - 0.108]	0.023 [0.018 - 0.030]	0.020 [0.017 - 0.024]	0.031 [0.024 - 0.041]
4	0.040 [0.026 - 0.061]	0.037 [0.027 - 0.051]	0.062 [0.035 - 0.106]	0.022 [0.016 - 0.028]	0.019 [0.015 - 0.023]	0.029 [0.022 - 0.039]
4.5	0.037 [0.023 - 0.058]	0.034 [0.024 - 0.049]	0.059 [0.033 - 0.104]	0.020 [0.015 - 0.027]	0.018 [0.014 - 0.022]	0.027 [0.020 - 0.038]
5	0.034 [0.021 - 0.054]	0.032 [0.022 - 0.046]	0.056 [0.030 - 0.102]	0.019 [0.014 - 0.026]	0.017 [0.013 - 0.021]	0.026 [0.018 - 0.036]
Age		30-34 yrs			>34 yrs	
Weight gain	Normal weight (n=12,302)	Underweight (n=2,460)	Overweight/Obese (n=4,749)	Normal weight (n=5,750)	Underweight (n=704)	Overweight/Obese (n=3,221)
-5	0.129 [0.099 - 0.167]	0.088 [0.080 - 0.097]	0.074 [0.060 - 0.090]	0.165 [0.117 - 0.227]	0.102 [0.089 - 0.116]	0.092 [0.081 - 0.103]
-4.5	0.117 [0.091 - 0.150]	0.081 [0.074 - 0.089]	0.067 [0.055 - 0.082]	0.150 [0.109 - 0.204]	0.094 [0.083 - 0.107]	0.088 [0.078 - 0.099]
-4	0.107 [0.084 - 0.135]	0.075 [0.069 - 0.082]	0.061 [0.051 - 0.074]	0.137 [0.100 - 0.185]	0.088 [0.077 - 0.099]	0.084 [0.075 - 0.095]
-3.5	0.097 [0.077 - 0.122]	0.070 [0.064 - 0.076]	0.057 [0.047 - 0.069]	0.125 [0.093 - 0.167]	0.081 [0.072 - 0.092]	0.080 [0.071 - 0.091]
-3	0.088 [0.070 - 0.110]	0.064 [0.059 - 0.070]	0.053 [0.044 - 0.065]	0.115 [0.085 - 0.153]	0.076 [0.067 - 0.085]	0.077 [0.068 - 0.087]
-2.5	0.079 [0.063 - 0.099]	0.059 [0.055 - 0.064]	0.050 [0.041 - 0.061]	0.105 [0.079 - 0.140]	0.070 [0.062 - 0.079]	0.074 [0.065 - 0.084]
-2	0.071 [0.057 - 0.089]	0.055 [0.051 - 0.059]	0.048 [0.039 - 0.059]	0.097 [0.073 - 0.128]	0.065 [0.058 - 0.074]	0.070 [0.062 - 0.080]
-1.5	0.064 [0.051 - 0.079]	0.051 [0.047 - 0.055]	0.047 [0.038 - 0.057]	0.089 [0.067 - 0.119]	0.061 [0.054 - 0.069]	0.067 [0.059 - 0.077]
-1	0.057 [0.046 - 0.071]	0.047 [0.043 - 0.051]	0.045 [0.037 - 0.056]	0.083 [0.062 - 0.110]	0.057 [0.050 - 0.064]	0.064 [0.056 - 0.075]
-0.5	0.051 [0.041 - 0.064]	0.043 [0.039 - 0.047]	0.044 [0.036 - 0.055]	0.077 [0.057 - 0.103]	0.053 [0.047 - 0.061]	0.062 [0.053 - 0.072]
Mean	0.045 [0.036 - 0.057]	0.040 [0.036 - 0.043]	0.043 [0.034 - 0.054]	0.072 [0.053 - 0.097]	0.050 [0.044 - 0.057]	0.059 [0.050 - 0.069]
0.5	0.041 [0.032 - 0.052]	0.036 [0.033 - 0.040]	0.042 [0.033 - 0.052]	0.067 [0.049 - 0.091]	0.047 [0.041 - 0.054]	0.056 [0.047 - 0.067]
1	0.036 [0.028 - 0.047]	0.034 [0.030 - 0.037]	0.040 [0.031 - 0.051]	0.063 [0.045 - 0.087]	0.044 [0.038 - 0.051]	0.054 [0.045 - 0.065]
1.5	0.033 [0.025 - 0.043]	0.031 [0.028 - 0.035]	0.038 [0.029 - 0.049]	0.059 [0.042 - 0.083]	0.042 [0.036 - 0.049]	0.051 [0.042 - 0.062]
2	0.030 [0.022 - 0.040]	0.028 [0.025 - 0.032]	0.036 [0.027 - 0.047]	0.056 [0.039 - 0.080]	0.039 [0.033 - 0.046]	0.049 [0.040 - 0.060]
2.5	0.027 [0.020 - 0.037]	0.026 [0.023 - 0.030]	0.034 [0.025 - 0.045]	0.053 [0.036 - 0.077]	0.037 [0.031 - 0.044]	0.047 [0.038 - 0.058]
3	0.025 [0.018 - 0.035]	0.024 [0.021 - 0.028]	0.031 [0.023 - 0.043]	0.050 [0.033 - 0.075]	0.035 [0.029 - 0.043]	0.045 [0.036 - 0.056]
3.5	0.023 [0.016 - 0.033]	0.022 [0.019 - 0.026]	0.029 [0.020 - 0.040]	0.048 [0.031 - 0.073]	0.033 [0.027 - 0.041]	0.043 [0.034 - 0.055]
4	0.022 [0.015 - 0.032]	0.020 [0.017 - 0.024]	0.026 [0.018 - 0.038]	0.045 [0.028 - 0.072]	0.032 [0.025 - 0.040]	0.041 [0.032 - 0.053]
4.5	0.020 [0.013 - 0.031]	0.019 [0.016 - 0.022]	0.024 [0.016 - 0.037]	0.043 [0.026 - 0.072]	0.030 [0.024 - 0.039]	0.039 [0.030 - 0.051]
5	0.020 [0.012 - 0.031]	0.017 [0.015 - 0.021]	0.022 [0.014 - 0.036]	0.041 [0.023 - 0.072]	0.029 [0.022 - 0.038]	0.037 [0.028 - 0.049]

3.5

Stillbirth

Age		<25 yrs			25-29 yrs	
Weight gain	Normal weight (n=1,324)	Underweight (n=698)	Overweight/Obese (n=314)	Normal weight (n=12,406)	Underweight (n=3,943)	Overweight/Obese (n=3,249)
-5	0.060 [0.030 - 0.115]	0.043 [0.025 - 0.071]	0.047 [0.024 - 0.089]	0.049 [0.034 - 0.071]	0.030 [0.024 - 0.036]	0.021 [0.014 - 0.032]
-4.5	0.047 [0.023 - 0.092]	0.035 [0.021 - 0.059]	0.042 [0.022 - 0.081]	0.036 [0.025 - 0.053]	0.023 [0.019 - 0.028]	0.018 [0.012 - 0.027]
-4	0.037 [0.018 - 0.074]	0.029 [0.017 - 0.049]	0.038 [0.019 - 0.074]	0.027 [0.018 - 0.040]	0.018 [0.014 - 0.022]	0.015 [0.010 - 0.022]
-3.5	0.029 [0.014 - 0.060]	0.024 [0.014 - 0.041]	0.034 [0.017 - 0.068]	0.020 [0.013 - 0.030]	0.014 [0.011 - 0.017]	0.012 [0.008 - 0.019]
-3	0.023 [0.010 - 0.049]	0.019 [0.011 - 0.034]	0.030 [0.015 - 0.062]	0.014 [0.009 - 0.022]	0.011 [0.008 - 0.014]	0.010 [0.007 - 0.016]
-2.5	0.018 [0.008 - 0.040]	0.016 [0.009 - 0.029]	0.027 [0.013 - 0.058]	0.011 [0.007 - 0.017]	0.008 [0.006 - 0.011]	0.009 [0.005 - 0.014]
-2	0.014 [0.006 - 0.033]	0.013 [0.007 - 0.025]	0.024 [0.011 - 0.054]	0.008 [0.005 - 0.013]	0.006 [0.005 - 0.008]	0.008 [0.005 - 0.012]
-1.5	0.011 [0.004 - 0.028]	0.011 [0.006 - 0.021]	0.022 [0.009 - 0.050]	0.006 [0.003 - 0.010]	0.005 [0.003 - 0.007]	0.007 [0.004 - 0.011]
-1	0.009 [0.003 - 0.024]	0.009 [0.005 - 0.018]	0.020 [0.008 - 0.047]	0.004 [0.002 - 0.008]	0.004 [0.003 - 0.005]	0.006 [0.003 - 0.010]
-0.5	0.007 [0.002 - 0.020]	0.008 [0.004 - 0.016]	0.017 [0.007 - 0.045]	0.003 [0.002 - 0.006]	0.003 [0.002 - 0.004]	0.005 [0.003 - 0.009]
Mean	0.005 [0.002 - 0.017]	0.006 [0.003 - 0.014]	0.016 [0.006 - 0.042]	0.003 [0.001 - 0.005]	0.002 [0.001 - 0.003]	0.005 [0.002 - 0.008]
0.5	0.004 [0.001 - 0.015]	0.005 [0.002 - 0.012]	0.014 [0.005 - 0.040]	0.002 [0.001 - 0.004]	0.002 [0.001 - 0.003]	0.004 [0.002 - 0.008]
1	0.003 [0.001 - 0.013]	0.005 [0.002 - 0.011]	0.012 [0.004 - 0.038]	0.002 [0.001 - 0.004]	0.001 [0.001 - 0.002]	0.004 [0.002 - 0.007]
1.5	0.003 [0.001 - 0.011]	0.004 [0.001 - 0.010]	0.011 [0.003 - 0.036]	0.001 [0.000 - 0.003]	0.001 [0.001 - 0.002]	0.003 [0.002 - 0.007]
2	0.002 [0.000 - 0.010]	0.003 [0.001 - 0.009]	0.010 [0.003 - 0.034]	0.001 [0.000 - 0.003]	0.001 [0.000 - 0.001]	0.003 [0.001 - 0.007]
2.5	0.002 [0.000 - 0.009]	0.003 [0.001 - 0.008]	0.009 [0.002 - 0.032]	0.001 [0.000 - 0.002]	0.001 [0.000 - 0.001]	0.003 [0.001 - 0.007]
3	0.001 [0.000 - 0.008]	0.002 [0.001 - 0.008]	0.008 [0.002 - 0.031]	0.001 [0.000 - 0.002]	0.000 [0.000 - 0.001]	0.003 [0.001 - 0.007]
3.5	0.001 [0.000 - 0.007]	0.002 [0.001 - 0.007]	0.007 [0.002 - 0.029]	0.000 [0.000 - 0.002]	0.000 [0.000 - 0.001]	0.002 [0.001 - 0.007]
4	0.001 [0.000 - 0.006]	0.002 [0.000 - 0.007]	0.006 [0.001 - 0.028]	0.000 [0.000 - 0.001]	0.000 [0.000 - 0.000]	0.002 [0.001 - 0.007]
4.5	0.001 [0.000 - 0.005]	0.001 [0.000 - 0.006]	0.006 [0.001 - 0.027]	0.000 [0.000 - 0.001]	0.000 [0.000 - 0.000]	0.002 [0.001 - 0.007]
5	0.001 [0.000 - 0.005]	0.001 [0.000 - 0.006]	0.005 [0.001 - 0.025]	0.000 [0.000 - 0.001]	0.000 [0.000 - 0.000]	0.002 [0.001 - 0.007]
Age		30-34 yrs			>34 yrs	
Weight gain	Normal weight (n=12,302)	Underweight (n=2,460)	Overweight/Obese (n=4,749)	Normal weight (n=5,750)	Underweight (n=703)	Overweight/Obese (n=3,221)
-5	0.024 [0.012 - 0.048]	0.015 [0.011 - 0.021]	0.014 [0.009 - 0.021]	0.021 [0.008 - 0.051]	0.025 [0.017 - 0.035]	0.016 [0.010 - 0.025]
-4.5	0.017 [0.008 - 0.035]	0.012 [0.008 - 0.016]	0.011 [0.007 - 0.018]	0.018 [0.008 - 0.044]	0.020 [0.014 - 0.028]	0.013 [0.008 - 0.021]
-4	0.012 [0.006 - 0.026]	0.009 [0.007 - 0.013]	0.009 [0.006 - 0.015]	0.016 [0.007 - 0.038]	0.016 [0.011 - 0.023]	0.010 [0.006 - 0.017]
-3.5	0.009 [0.004 - 0.019]	0.008 [0.005 - 0.011]	0.008 [0.005 - 0.013]	0.014 [0.006 - 0.033]	0.012 [0.008 - 0.019]	0.008 [0.004 - 0.014]
-3	0.006 [0.003 - 0.014]	0.006 [0.004 - 0.009]	0.007 [0.004 - 0.011]	0.013 [0.005 - 0.029]	0.010 [0.006 - 0.015]	0.006 [0.003 - 0.012]
-2.5	0.004 [0.002 - 0.011]	0.005 [0.003 - 0.008]	0.006 [0.003 - 0.010]	0.011 [0.005 - 0.026]	0.008 [0.005 - 0.012]	0.005 [0.003 - 0.010]
-2	0.003 [0.001 - 0.009]	0.004 [0.003 - 0.006]	0.005 [0.003 - 0.009]	0.010 [0.004 - 0.023]	0.006 [0.004 - 0.010]	0.004 [0.002 - 0.009]
-1.5	0.003 [0.001 - 0.007]	0.004 [0.002 - 0.006]	0.004 [0.002 - 0.008]	0.009 [0.004 - 0.021]	0.004 [0.003 - 0.008]	0.003 [0.001 - 0.007]
-1	0.002 [0.001 - 0.006]	0.003 [0.002 - 0.005]	0.004 [0.002 - 0.007]	0.008 [0.004 - 0.020]	0.003 [0.002 - 0.006]	0.003 [0.001 - 0.007]
-0.5	0.002 [0.001 - 0.005]	0.002 [0.002 - 0.004]	0.003 [0.002 - 0.006]	0.008 [0.003 - 0.019]	0.003 [0.001 - 0.005]	0.002 [0.001 - 0.006]
Mean	0.002 [0.000 - 0.005]	0.002 [0.001 - 0.003]	0.003 [0.001 - 0.006]	0.007 [0.003 - 0.018]	0.002 [0.001 - 0.004]	0.002 [0.001 - 0.005]
0.5	0.001 [0.000 - 0.004]	0.002 [0.001 - 0.003]	0.003 [0.001 - 0.006]	0.007 [0.003 - 0.017]	0.002 [0.001 - 0.004]	0.001 [0.000 - 0.005]
1	0.001 [0.000 - 0.004]	0.001 [0.001 - 0.003]	0.002 [0.001 - 0.006]	0.006 [0.002 - 0.017]	0.001 [0.000 - 0.003]	0.001 [0.000 - 0.005]
1.5	0.001 [0.000 - 0.004]	0.001 [0.001 - 0.002]	0.002 [0.001 - 0.005]	0.006 [0.002 - 0.017]	0.001 [0.000 - 0.003]	0.001 [0.000 - 0.005]
2	0.001 [0.000 - 0.005]	0.001 [0.001 - 0.002]	0.002 [0.001 - 0.005]	0.005 [0.002 - 0.017]	0.001 [0.000 - 0.003]	0.001 [0.000 - 0.005]
2.5	0.001 [0.000 - 0.005]	0.001 [0.000 - 0.002]	0.002 [0.001 - 0.005]	0.005 [0.002 - 0.017]	0.001 [0.000 - 0.003]	0.001 [0.000 - 0.006]
3	0.001 [0.000 - 0.005]	0.001 [0.000 - 0.002]	0.002 [0.000 - 0.006]	0.005 [0.001 - 0.017]	0.001 [0.000 - 0.003]	0.001 [0.000 - 0.006]
3.5	0.001 [0.000 - 0.006]	0.001 [0.000 - 0.002]	0.001 [0.000 - 0.006]	0.005 [0.001 - 0.018]	0.000 [0.000 - 0.003]	0.000 [0.000 - 0.007]
4	0.001 [0.000 - 0.006]	0.001 [0.000 - 0.002]	0.001 [0.000 - 0.006]	0.004 [0.001 - 0.019]	0.000 [0.000 - 0.003]	0.000 [0.000 - 0.008]
4.5	0.001 [0.000 - 0.007]	0.001 [0.000 - 0.002]	0.001 [0.000 - 0.007]	0.004 [0.001 - 0.020]	0.000 [0.000 - 0.003]	0.000 [0.000 - 0.009]
5	0.001 [0.000 - 0.008]	0.001 [0.000 - 0.002]	0.001 [0.000 - 0.007]	0.004 [0.001 - 0.021]	0.000 [0.000 - 0.003]	0.000 [0.000 - 0.010]

3.6

Macrosomia

Age		<25 yrs			25-29 yrs	
Weight gain	Normal weight (n=1,317)	Underweight (n=697)	Overweight/Obese (n=313)	Normal weight (n=12,390)	Underweight (n=3,940)	Overweight/Obese (n=3,236)
-5	0.003 [0.000 - 0.016]	0.012 [0.006 - 0.023]	0.052 [0.027 - 0.098]	0.002 [0.001 - 0.005]	0.013 [0.011 - 0.017]	0.043 [0.035 - 0.054]
-4.5	0.003 [0.001 - 0.016]	0.013 [0.007 - 0.024]	0.054 [0.029 - 0.098]	0.003 [0.001 - 0.006]	0.014 [0.012 - 0.018]	0.046 [0.037 - 0.056]
-4	0.003 [0.001 - 0.016]	0.014 [0.008 - 0.025]	0.056 [0.031 - 0.098]	0.003 [0.001 - 0.006]	0.016 [0.013 - 0.019]	0.048 [0.039 - 0.058]
-3.5	0.003 [0.001 - 0.016]	0.015 [0.009 - 0.026]	0.057 [0.033 - 0.099]	0.003 [0.002 - 0.007]	0.017 [0.014 - 0.020]	0.050 [0.042 - 0.060]
-3	0.004 [0.001 - 0.017]	0.016 [0.009 - 0.027]	0.059 [0.035 - 0.100]	0.004 [0.002 - 0.007]	0.018 [0.015 - 0.021]	0.053 [0.045 - 0.063]
-2.5	0.004 [0.001 - 0.017]	0.017 [0.010 - 0.029]	0.061 [0.037 - 0.101]	0.004 [0.002 - 0.008]	0.019 [0.017 - 0.023]	0.056 [0.047 - 0.065]
-2	0.005 [0.001 - 0.017]	0.019 [0.012 - 0.030]	0.064 [0.039 - 0.102]	0.005 [0.003 - 0.008]	0.021 [0.018 - 0.024]	0.058 [0.050 - 0.068]
-1.5	0.005 [0.001 - 0.017]	0.020 [0.013 - 0.032]	0.066 [0.041 - 0.103]	0.005 [0.003 - 0.009]	0.022 [0.019 - 0.026]	0.061 [0.053 - 0.071]
-1	0.005 [0.002 - 0.018]	0.022 [0.014 - 0.033]	0.068 [0.044 - 0.104]	0.006 [0.004 - 0.010]	0.024 [0.021 - 0.027]	0.064 [0.056 - 0.074]
-0.5	0.006 [0.002 - 0.018]	0.023 [0.016 - 0.035]	0.070 [0.046 - 0.106]	0.007 [0.005 - 0.011]	0.026 [0.023 - 0.029]	0.068 [0.059 - 0.077]
Mean	0.007 [0.002 - 0.019]	0.025 [0.017 - 0.037]	0.073 [0.048 - 0.108]	0.008 [0.005 - 0.012]	0.028 [0.025 - 0.031]	0.071 [0.062 - 0.081]
0.5	0.007 [0.003 - 0.019]	0.027 [0.019 - 0.039]	0.075 [0.050 - 0.111]	0.009 [0.006 - 0.013]	0.030 [0.027 - 0.033]	0.075 [0.066 - 0.084]
1	0.008 [0.003 - 0.020]	0.029 [0.021 - 0.041]	0.077 [0.052 - 0.113]	0.010 [0.007 - 0.014]	0.032 [0.029 - 0.035]	0.078 [0.069 - 0.088]
1.5	0.009 [0.004 - 0.020]	0.031 [0.023 - 0.043]	0.080 [0.054 - 0.117]	0.011 [0.008 - 0.016]	0.034 [0.031 - 0.038]	0.082 [0.073 - 0.093]
2	0.009 [0.004 - 0.021]	0.034 [0.025 - 0.046]	0.083 [0.056 - 0.121]	0.013 [0.010 - 0.018]	0.037 [0.034 - 0.040]	0.086 [0.076 - 0.097]
2.5	0.010 [0.005 - 0.022]	0.036 [0.027 - 0.048]	0.085 [0.058 - 0.125]	0.015 [0.011 - 0.019]	0.040 [0.036 - 0.043]	0.090 [0.080 - 0.102]
3	0.011 [0.005 - 0.023]	0.039 [0.029 - 0.051]	0.088 [0.059 - 0.130]	0.016 [0.012 - 0.022]	0.042 [0.039 - 0.046]	0.095 [0.083 - 0.108]
3.5	0.012 [0.006 - 0.025]	0.042 [0.032 - 0.055]	0.091 [0.060 - 0.135]	0.018 [0.014 - 0.024]	0.046 [0.042 - 0.050]	0.099 [0.087 - 0.114]
4	0.013 [0.007 - 0.027]	0.045 [0.034 - 0.059]	0.094 [0.062 - 0.141]	0.021 [0.016 - 0.027]	0.049 [0.045 - 0.054]	0.104 [0.090 - 0.120]
4.5	0.015 [0.007 - 0.029]	0.048 [0.037 - 0.063]	0.097 [0.063 - 0.148]	0.023 [0.018 - 0.030]	0.052 [0.048 - 0.058]	0.109 [0.094 - 0.126]
5	0.016 [0.008 - 0.032]	0.052 [0.039 - 0.068]	0.100 [0.064 - 0.155]	0.026 [0.020 - 0.034]	0.056 [0.051 - 0.062]	0.114 [0.098 - 0.133]
Age		30-34 yrs			>34 yrs	
Weight gain	Normal weight (n=12,281)	Underweight (n=2,458)	Overweight/Obese (n=4,738)	Normal weight (n=5,735)	Underweight (n=703)	Overweight/Obese (n=3,211)
-5	0.003 [0.001 - 0.009]	0.014 [0.011 - 0.017]	0.042 [0.034 - 0.051]	0.000 [0.000 - 0.033]	0.016 [0.012 - 0.021]	0.041 [0.034 - 0.050]
-4.5	0.003 [0.001 - 0.009]	0.015 [0.013 - 0.018]	0.044 [0.037 - 0.053]	0.000 [0.000 - 0.028]	0.017 [0.013 - 0.022]	0.044 [0.036 - 0.053]
-4	0.003 [0.001 - 0.009]	0.016 [0.014 - 0.020]	0.047 [0.040 - 0.056]	0.000 [0.000 - 0.023]	0.018 [0.014 - 0.023]	0.046 [0.039 - 0.055]
-3.5	0.004 [0.002 - 0.010]	0.018 [0.015 - 0.021]	0.051 [0.043 - 0.060]	0.000 [0.000 - 0.020]	0.020 [0.016 - 0.025]	0.049 [0.041 - 0.058]
-3	0.005 [0.002 - 0.010]	0.019 [0.017 - 0.023]	0.054 [0.047 - 0.064]	0.000 [0.000 - 0.017]	0.021 [0.017 - 0.026]	0.052 [0.044 - 0.060]
-2.5	0.005 [0.003 - 0.011]	0.021 [0.018 - 0.024]	0.058 [0.050 - 0.068]	0.000 [0.000 - 0.016]	0.023 [0.018 - 0.028]	0.054 [0.047 - 0.063]
-2	0.006 [0.003 - 0.012]	0.023 [0.020 - 0.026]	0.062 [0.054 - 0.072]	0.000 [0.000 - 0.014]	0.024 [0.020 - 0.029]	0.057 [0.050 - 0.066]
-1.5	0.007 [0.004 - 0.013]	0.025 [0.022 - 0.028]	0.067 [0.058 - 0.076]	0.000 [0.000 - 0.013]	0.026 [0.022 - 0.031]	0.061 [0.053 - 0.070]
-1	0.008 [0.005 - 0.014]	0.027 [0.024 - 0.030]	0.071 [0.062 - 0.081]	0.000 [0.000 - 0.013]	0.028 [0.024 - 0.033]	0.064 [0.056 - 0.073]
-0.5	0.009 [0.006 - 0.015]	0.029 [0.026 - 0.032]	0.075 [0.066 - 0.086]	0.001 [0.000 - 0.013]	0.030 [0.026 - 0.035]	0.067 [0.059 - 0.077]
Mean	0.011 [0.007 - 0.017]	0.031 [0.028 - 0.035]	0.080 [0.070 - 0.091]	0.001 [0.000 - 0.013]	0.032 [0.028 - 0.037]	0.071 [0.062 - 0.081]
0.5	0.012 [0.008 - 0.019]	0.034 [0.031 - 0.038]	0.085 [0.075 - 0.097]	0.001 [0.000 - 0.014]	0.034 [0.030 - 0.040]	0.075 [0.065 - 0.086]
1	0.014 [0.010 - 0.021]	0.037 [0.033 - 0.040]	0.090 [0.079 - 0.102]	0.002 [0.000 - 0.015]	0.037 [0.032 - 0.042]	0.079 [0.069 - 0.091]
1.5	0.016 [0.011 - 0.023]	0.040 [0.036 - 0.044]	0.095 [0.083 - 0.108]	0.003 [0.000 - 0.017]	0.040 [0.034 - 0.045]	0.083 [0.072 - 0.096]
2	0.018 [0.013 - 0.026]	0.043 [0.039 - 0.047]	0.100 [0.087 - 0.114]	0.004 [0.001 - 0.019]	0.042 [0.037 - 0.049]	0.088 [0.076 - 0.102]
2.5	0.021 [0.014 - 0.029]	0.047 [0.043 - 0.051]	0.105 [0.092 - 0.121]	0.005 [0.001 - 0.022]	0.045 [0.039 - 0.052]	0.093 [0.079 - 0.108]
3	0.023 [0.016 - 0.033]	0.050 [0.046 - 0.055]	0.111 [0.096 - 0.127]	0.008 [0.002 - 0.027]	0.049 [0.042 - 0.056]	0.098 [0.083 - 0.115]
3.5	0.026 [0.018 - 0.036]	0.055 [0.050 - 0.060]	0.116 [0.100 - 0.134]	0.010 [0.003 - 0.032]	0.052 [0.045 - 0.060]	0.103 [0.086 - 0.122]
4	0.028 [0.020 - 0.040]	0.059 [0.054 - 0.065]	0.121 [0.104 - 0.140]	0.013 [0.005 - 0.039]	0.056 [0.048 - 0.065]	0.108 [0.090 - 0.129]
4.5	0.031 [0.022 - 0.044]	0.064 [0.058 - 0.070]	0.126 [0.107 - 0.147]	0.017 [0.006 - 0.047]	0.060 [0.051 - 0.070]	0.114 [0.094 - 0.137]
5	0.034 [0.023 - 0.049]	0.069 [0.062 - 0.076]	0.131 [0.111 - 0.154]	0.022 [0.008 - 0.058]	0.064 [0.054 - 0.076]	0.120 [0.098 - 0.146]

3.7

Small for gestational age

Age		<25 yrs			25-29 yrs	
Weight gain	Normal weight (n=1,316)	Underweight (n=697)	Overweight/Obese (n=313)	Normal weight (n=12,386)	Underweight (n=3,938)	Overweight/Obese (n=3,236)
-5	0.081 [0.049 - 0.131]	0.066 [0.044 - 0.099]	0.039 [0.018 - 0.081]	0.081 [0.060 - 0.108]	0.060 [0.049 - 0.074]	0.023 [0.017 - 0.032]
-4.5	0.079 [0.049 - 0.125]	0.062 [0.042 - 0.091]	0.038 [0.018 - 0.078]	0.079 [0.060 - 0.103]	0.059 [0.049 - 0.071]	0.023 [0.017 - 0.031]
-4	0.077 [0.049 - 0.119]	0.058 [0.040 - 0.084]	0.038 [0.019 - 0.075]	0.077 [0.060 - 0.099]	0.057 [0.048 - 0.067]	0.023 [0.017 - 0.030]
-3.5	0.076 [0.050 - 0.114]	0.055 [0.038 - 0.078]	0.037 [0.019 - 0.072]	0.075 [0.060 - 0.095]	0.054 [0.046 - 0.064]	0.023 [0.017 - 0.029]
-3	0.074 [0.050 - 0.109]	0.052 [0.037 - 0.073]	0.037 [0.019 - 0.070]	0.073 [0.059 - 0.091]	0.051 [0.044 - 0.060]	0.022 [0.017 - 0.029]
-2.5	0.073 [0.050 - 0.105]	0.049 [0.035 - 0.068]	0.037 [0.020 - 0.068]	0.071 [0.058 - 0.087]	0.048 [0.041 - 0.056]	0.022 [0.017 - 0.028]
-2	0.071 [0.050 - 0.100]	0.046 [0.033 - 0.063]	0.036 [0.020 - 0.066]	0.068 [0.056 - 0.083]	0.045 [0.039 - 0.052]	0.022 [0.017 - 0.028]
-1.5	0.070 [0.050 - 0.097]	0.043 [0.031 - 0.060]	0.036 [0.020 - 0.065]	0.066 [0.054 - 0.079]	0.042 [0.036 - 0.048]	0.021 [0.017 - 0.027]
-1	0.068 [0.049 - 0.093]	0.041 [0.030 - 0.056]	0.036 [0.020 - 0.063]	0.063 [0.052 - 0.075]	0.039 [0.034 - 0.045]	0.021 [0.017 - 0.027]
-0.5	0.067 [0.049 - 0.090]	0.039 [0.028 - 0.053]	0.035 [0.020 - 0.063]	0.060 [0.050 - 0.072]	0.036 [0.031 - 0.042]	0.021 [0.016 - 0.026]
Mean	0.065 [0.048 - 0.087]	0.037 [0.027 - 0.051]	0.035 [0.019 - 0.062]	0.057 [0.047 - 0.068]	0.034 [0.029 - 0.039]	0.021 [0.016 - 0.026]
0.5	0.064 [0.048 - 0.085]	0.035 [0.025 - 0.048]	0.035 [0.019 - 0.062]	0.054 [0.045 - 0.064]	0.032 [0.027 - 0.037]	0.020 [0.016 - 0.026]
1	0.063 [0.047 - 0.083]	0.033 [0.024 - 0.046]	0.034 [0.019 - 0.062]	0.051 [0.042 - 0.061]	0.030 [0.026 - 0.035]	0.020 [0.015 - 0.026]
1.5	0.061 [0.046 - 0.082]	0.032 [0.023 - 0.044]	0.034 [0.018 - 0.063]	0.048 [0.040 - 0.058]	0.028 [0.024 - 0.033]	0.020 [0.015 - 0.026]
2	0.060 [0.044 - 0.081]	0.031 [0.022 - 0.043]	0.034 [0.018 - 0.064]	0.046 [0.038 - 0.056]	0.027 [0.023 - 0.032]	0.020 [0.015 - 0.026]
2.5	0.059 [0.043 - 0.080]	0.030 [0.021 - 0.042]	0.033 [0.017 - 0.065]	0.044 [0.036 - 0.054]	0.027 [0.022 - 0.031]	0.019 [0.014 - 0.026]
3	0.057 [0.041 - 0.079]	0.028 [0.020 - 0.041]	0.033 [0.016 - 0.066]	0.043 [0.035 - 0.053]	0.026 [0.022 - 0.031]	0.019 [0.014 - 0.026]
3.5	0.056 [0.040 - 0.079]	0.027 [0.019 - 0.040]	0.033 [0.016 - 0.067]	0.042 [0.033 - 0.052]	0.026 [0.021 - 0.031]	0.019 [0.013 - 0.026]
4	0.055 [0.038 - 0.078]	0.027 [0.018 - 0.039]	0.032 [0.015 - 0.069]	0.041 [0.032 - 0.051]	0.025 [0.021 - 0.031]	0.019 [0.013 - 0.027]
4.5	0.054 [0.037 - 0.078]	0.026 [0.017 - 0.039]	0.032 [0.014 - 0.071]	0.040 [0.031 - 0.051]	0.025 [0.021 - 0.031]	0.018 [0.013 - 0.027]
5	0.053 [0.035 - 0.079]	0.025 [0.016 - 0.039]	0.032 [0.014 - 0.073]	0.039 [0.030 - 0.051]	0.025 [0.020 - 0.031]	0.018 [0.012 - 0.027]
Age		30-34 yrs			>34 yrs	
Weight gain	Normal weight (n=12,275)	Underweight (n=2,458)	Overweight/Obese (n=4,735)	Normal weight (n=5,730)	Underweight (n=703)	Overweight/Obese (n=3,208)
-5	0.097 [0.074 - 0.125]	0.049 [0.043 - 0.056]	0.026 [0.021 - 0.032]	0.094 [0.061 - 0.143]	0.035 [0.027 - 0.044]	0.020 [0.016 - 0.027]
-4.5	0.091 [0.071 - 0.116]	0.047 [0.041 - 0.053]	0.025 [0.021 - 0.031]	0.089 [0.059 - 0.132]	0.034 [0.027 - 0.043]	0.020 [0.015 - 0.026]
-4	0.085 [0.067 - 0.107]	0.045 [0.040 - 0.050]	0.025 [0.020 - 0.030]	0.083 [0.057 - 0.121]	0.034 [0.027 - 0.042]	0.020 [0.015 - 0.025]
-3.5	0.080 [0.064 - 0.099]	0.043 [0.038 - 0.048]	0.024 [0.020 - 0.029]	0.078 [0.054 - 0.111]	0.033 [0.027 - 0.040]	0.019 [0.015 - 0.025]
-3	0.075 [0.061 - 0.093]	0.041 [0.037 - 0.045]	0.023 [0.019 - 0.028]	0.073 [0.052 - 0.103]	0.032 [0.026 - 0.039]	0.019 [0.015 - 0.024]
-2.5	0.071 [0.058 - 0.087]	0.039 [0.035 - 0.043]	0.022 [0.019 - 0.027]	0.069 [0.049 - 0.095]	0.031 [0.025 - 0.038]	0.018 [0.014 - 0.024]
-2	0.067 [0.055 - 0.081]	0.037 [0.034 - 0.041]	0.022 [0.018 - 0.026]	0.065 [0.047 - 0.088]	0.030 [0.025 - 0.036]	0.018 [0.014 - 0.023]
-1.5	0.063 [0.052 - 0.076]	0.036 [0.032 - 0.039]	0.021 [0.017 - 0.026]	0.060 [0.044 - 0.083]	0.029 [0.024 - 0.035]	0.018 [0.014 - 0.023]
-1	0.060 [0.049 - 0.072]	0.034 [0.031 - 0.038]	0.020 [0.017 - 0.025]	0.057 [0.041 - 0.077]	0.028 [0.023 - 0.033]	0.017 [0.013 - 0.023]
-0.5	0.057 [0.047 - 0.068]	0.033 [0.030 - 0.036]	0.020 [0.016 - 0.024]	0.053 [0.038 - 0.073]	0.026 [0.022 - 0.032]	0.017 [0.013 - 0.023]
Mean	0.054 [0.044 - 0.065]	0.031 [0.028 - 0.035]	0.019 [0.015 - 0.024]	0.050 [0.036 - 0.069]	0.025 [0.021 - 0.031]	0.017 [0.012 - 0.023]
0.5	0.051 [0.042 - 0.062]	0.030 [0.027 - 0.033]	0.019 [0.015 - 0.023]	0.047 [0.033 - 0.066]	0.024 [0.020 - 0.030]	0.016 [0.012 - 0.023]
1	0.049 [0.040 - 0.059]	0.029 [0.026 - 0.032]	0.018 [0.014 - 0.023]	0.044 [0.030 - 0.063]	0.023 [0.018 - 0.028]	0.016 [0.011 - 0.023]
1.5	0.046 [0.038 - 0.057]	0.027 [0.024 - 0.031]	0.017 [0.013 - 0.023]	0.041 [0.027 - 0.061]	0.022 [0.017 - 0.027]	0.016 [0.011 - 0.023]
2	0.044 [0.036 - 0.055]	0.026 [0.023 - 0.030]	0.017 [0.013 - 0.022]	0.038 [0.025 - 0.058]	0.021 [0.016 - 0.026]	0.015 [0.010 - 0.023]
2.5	0.042 [0.034 - 0.053]	0.025 [0.022 - 0.028]	0.016 [0.012 - 0.022]	0.036 [0.023 - 0.056]	0.020 [0.015 - 0.026]	0.015 [0.010 - 0.023]
3	0.041 [0.032 - 0.052]	0.024 [0.021 - 0.027]	0.016 [0.012 - 0.022]	0.033 [0.020 - 0.055]	0.019 [0.014 - 0.025]	0.015 [0.010 - 0.023]
3.5	0.039 [0.030 - 0.051]	0.023 [0.020 - 0.026]	0.015 [0.011 - 0.021]	0.031 [0.018 - 0.053]	0.018 [0.013 - 0.024]	0.014 [0.009 - 0.023]
4	0.038 [0.029 - 0.050]	0.022 [0.018 - 0.026]	0.015 [0.011 - 0.021]	0.029 [0.017 - 0.051]	0.017 [0.012 - 0.024]	0.014 [0.009 - 0.023]
4.5	0.037 [0.027 - 0.049]	0.021 [0.017 - 0.025]	0.014 [0.010 - 0.021]	0.027 [0.015 - 0.050]	0.016 [0.011 - 0.023]	0.014 [0.008 - 0.023]
5	0.035 [0.026 - 0.049]	0.020 [0.017 - 0.024]	0.014 [0.010 - 0.020]	0.026 [0.013 - 0.048]	0.015 [0.010 - 0.023]	0.014 [0.008 - 0.023]

3.8

Large for gestational age

Age		<25 yrs			25-29 yrs	
Weight gain	Normal weight (n=1,316)	Underweight (n=697)	Overweight/Obese (n=313)	Normal weight (n=12,386)	Underweight (n=3,938)	Overweight/Obese (n=3,236)
-5	0.116 [0.071 - 0.182]	0.157 [0.115 - 0.211]	0.214 [0.147 - 0.302]	0.074 [0.056 - 0.098]	0.107 [0.095 - 0.122]	0.175 [0.157 - 0.195]
-4.5	0.110 [0.070 - 0.170]	0.147 [0.109 - 0.196]	0.215 [0.149 - 0.300]	0.071 [0.055 - 0.092]	0.106 [0.094 - 0.118]	0.178 [0.160 - 0.196]
-4	0.105 [0.068 - 0.159]	0.138 [0.104 - 0.182]	0.215 [0.151 - 0.297]	0.068 [0.054 - 0.087]	0.104 [0.093 - 0.115]	0.180 [0.163 - 0.198]
-3.5	0.101 [0.067 - 0.149]	0.130 [0.098 - 0.170]	0.213 [0.151 - 0.292]	0.066 [0.053 - 0.083]	0.103 [0.093 - 0.113]	0.183 [0.167 - 0.200]
-3	0.096 [0.065 - 0.140]	0.122 [0.093 - 0.159]	0.210 [0.150 - 0.287]	0.065 [0.052 - 0.079]	0.102 [0.092 - 0.112]	0.186 [0.170 - 0.202]
-2.5	0.092 [0.063 - 0.132]	0.116 [0.089 - 0.149]	0.206 [0.148 - 0.280]	0.063 [0.052 - 0.077]	0.101 [0.092 - 0.110]	0.189 [0.173 - 0.205]
-2	0.088 [0.061 - 0.124]	0.109 [0.084 - 0.141]	0.201 [0.144 - 0.273]	0.063 [0.052 - 0.075]	0.101 [0.093 - 0.110]	0.192 [0.176 - 0.208]
-1.5	0.084 [0.059 - 0.118]	0.104 [0.081 - 0.133]	0.195 [0.140 - 0.265]	0.062 [0.052 - 0.074]	0.101 [0.093 - 0.110]	0.195 [0.180 - 0.211]
-1	0.080 [0.057 - 0.112]	0.099 [0.077 - 0.127]	0.189 [0.136 - 0.257]	0.063 [0.053 - 0.074]	0.102 [0.094 - 0.110]	0.198 [0.183 - 0.214]
-0.5	0.077 [0.055 - 0.107]	0.096 [0.075 - 0.122]	0.183 [0.131 - 0.249]	0.063 [0.054 - 0.074]	0.103 [0.096 - 0.111]	0.201 [0.186 - 0.217]
Mean	0.074 [0.053 - 0.103]	0.093 [0.072 - 0.118]	0.177 [0.126 - 0.242]	0.064 [0.055 - 0.075]	0.105 [0.098 - 0.113]	0.205 [0.190 - 0.221]
0.5	0.072 [0.051 - 0.099]	0.090 [0.071 - 0.115]	0.171 [0.122 - 0.235]	0.066 [0.057 - 0.077]	0.108 [0.100 - 0.116]	0.209 [0.193 - 0.225]
1	0.069 [0.050 - 0.096]	0.089 [0.070 - 0.113]	0.166 [0.117 - 0.229]	0.068 [0.058 - 0.079]	0.111 [0.103 - 0.119]	0.212 [0.196 - 0.229]
1.5	0.067 [0.048 - 0.093]	0.089 [0.070 - 0.113]	0.161 [0.113 - 0.225]	0.070 [0.061 - 0.081]	0.115 [0.107 - 0.123]	0.216 [0.200 - 0.234]
2	0.066 [0.047 - 0.092]	0.090 [0.071 - 0.114]	0.157 [0.109 - 0.221]	0.073 [0.063 - 0.084]	0.119 [0.111 - 0.128]	0.220 [0.203 - 0.238]
2.5	0.065 [0.046 - 0.091]	0.092 [0.073 - 0.117]	0.154 [0.106 - 0.219]	0.076 [0.065 - 0.088]	0.124 [0.115 - 0.133]	0.224 [0.207 - 0.243]
3	0.064 [0.046 - 0.091]	0.096 [0.075 - 0.121]	0.153 [0.104 - 0.218]	0.079 [0.068 - 0.092]	0.129 [0.120 - 0.138]	0.229 [0.210 - 0.248]
3.5	0.064 [0.045 - 0.091]	0.100 [0.079 - 0.127]	0.152 [0.102 - 0.219]	0.083 [0.071 - 0.096]	0.135 [0.125 - 0.145]	0.233 [0.213 - 0.254]
4	0.065 [0.045 - 0.093]	0.106 [0.083 - 0.135]	0.152 [0.101 - 0.221]	0.087 [0.075 - 0.101]	0.140 [0.130 - 0.151]	0.238 [0.217 - 0.260]
4.5	0.066 [0.045 - 0.095]	0.114 [0.089 - 0.144]	0.153 [0.101 - 0.225]	0.091 [0.078 - 0.107]	0.146 [0.135 - 0.158]	0.242 [0.220 - 0.266]
5	0.068 [0.046 - 0.098]	0.123 [0.096 - 0.156]	0.155 [0.101 - 0.231]	0.096 [0.081 - 0.113]	0.153 [0.141 - 0.165]	0.247 [0.223 - 0.273]
Age		30-34 yrs			>34 yrs	
Weight gain	Normal weight (n=12,275)	Underweight (n=2,458)	Overweight/Obese (n=4,735)	Normal weight (n=5,730)	Underweight (n=703)	Overweight/Obese (n=3,208)
-5	0.068 [0.050 - 0.093]	0.101 [0.091 - 0.113]	0.180 [0.165 - 0.197]	0.080 [0.049 - 0.129]	0.138 [0.123 - 0.154]	0.215 [0.198 - 0.234]
-4.5	0.069 [0.052 - 0.091]	0.102 [0.093 - 0.112]	0.183 [0.168 - 0.199]	0.077 [0.048 - 0.120]	0.138 [0.124 - 0.153]	0.218 [0.201 - 0.236]
-4	0.070 [0.054 - 0.090]	0.103 [0.094 - 0.113]	0.186 [0.171 - 0.201]	0.074 [0.048 - 0.113]	0.139 [0.126 - 0.153]	0.221 [0.204 - 0.239]
-3.5	0.070 [0.056 - 0.089]	0.104 [0.096 - 0.113]	0.189 [0.175 - 0.204]	0.072 [0.048 - 0.107]	0.139 [0.127 - 0.153]	0.224 [0.207 - 0.242]
-3	0.071 [0.057 - 0.088]	0.106 [0.098 - 0.115]	0.193 [0.179 - 0.208]	0.071 [0.048 - 0.103]	0.140 [0.129 - 0.153]	0.227 [0.210 - 0.245]
-2.5	0.072 [0.059 - 0.088]	0.108 [0.100 - 0.116]	0.197 [0.183 - 0.211]	0.070 [0.048 - 0.100]	0.142 [0.130 - 0.154]	0.230 [0.213 - 0.248]
-2	0.073 [0.061 - 0.088]	0.110 [0.103 - 0.118]	0.201 [0.187 - 0.216]	0.070 [0.049 - 0.099]	0.143 [0.132 - 0.155]	0.234 [0.217 - 0.251]
-1.5	0.074 [0.062 - 0.089]	0.113 [0.106 - 0.121]	0.206 [0.192 - 0.221]	0.070 [0.050 - 0.098]	0.145 [0.134 - 0.157]	0.237 [0.220 - 0.255]
-1	0.076 [0.064 - 0.090]	0.116 [0.109 - 0.124]	0.211 [0.196 - 0.226]	0.071 [0.051 - 0.099]	0.147 [0.136 - 0.159]	0.241 [0.223 - 0.259]
-0.5	0.078 [0.066 - 0.091]	0.120 [0.112 - 0.128]	0.216 [0.201 - 0.231]	0.073 [0.053 - 0.101]	0.149 [0.138 - 0.161]	0.245 [0.227 - 0.263]
Mean	0.080 [0.068 - 0.093]	0.124 [0.116 - 0.131]	0.222 [0.207 - 0.237]	0.076 [0.055 - 0.104]	0.152 [0.141 - 0.164]	0.249 [0.230 - 0.268]
0.5	0.082 [0.070 - 0.095]	0.128 [0.120 - 0.136]	0.227 [0.212 - 0.244]	0.080 [0.058 - 0.109]	0.155 [0.144 - 0.167]	0.253 [0.234 - 0.273]
1	0.084 [0.072 - 0.098]	0.132 [0.124 - 0.140]	0.234 [0.217 - 0.250]	0.084 [0.062 - 0.114]	0.158 [0.147 - 0.171]	0.257 [0.237 - 0.278]
1.5	0.087 [0.074 - 0.101]	0.136 [0.128 - 0.145]	0.240 [0.223 - 0.258]	0.090 [0.066 - 0.121]	0.162 [0.150 - 0.175]	0.262 [0.241 - 0.284]
2	0.090 [0.077 - 0.104]	0.141 [0.133 - 0.150]	0.246 [0.229 - 0.265]	0.096 [0.070 - 0.128]	0.166 [0.153 - 0.180]	0.267 [0.244 - 0.290]
2.5	0.093 [0.080 - 0.108]	0.146 [0.137 - 0.155]	0.253 [0.234 - 0.273]	0.102 [0.076 - 0.137]	0.170 [0.157 - 0.185]	0.272 [0.248 - 0.297]
3	0.097 [0.082 - 0.113]	0.151 [0.142 - 0.161]	0.260 [0.240 - 0.281]	0.110 [0.081 - 0.147]	0.175 [0.160 - 0.190]	0.277 [0.252 - 0.304]
3.5	0.100 [0.085 - 0.118]	0.156 [0.146 - 0.167]	0.267 [0.246 - 0.290]	0.118 [0.087 - 0.158]	0.179 [0.164 - 0.196]	0.282 [0.256 - 0.311]
4	0.105 [0.088 - 0.123]	0.162 [0.151 - 0.173]	0.275 [0.252 - 0.299]	0.126 [0.092 - 0.170]	0.184 [0.168 - 0.202]	0.288 [0.259 - 0.319]
4.5	0.109 [0.092 - 0.130]	0.168 [0.156 - 0.180]	0.282 [0.257 - 0.308]	0.134 [0.097 - 0.183]	0.189 [0.172 - 0.208]	0.294 [0.263 - 0.328]
5	0.114 [0.095 - 0.137]	0.174 [0.161 - 0.188]	0.289 [0.262 - 0.318]	0.143 [0.102 - 0.196]	0.195 [0.175 - 0.216]	0.300 [0.266 - 0.337]

3.9

All disease

Age		<25 yrs			25-29 yrs	
Weight gain	Normal weight (n=1,324)	Underweight (n=698)	Overweight/Obese (n=314)	Normal weight (n=12,406)	Underweight (n=3,943)	Overweight/Obese (n=3,249)
-5	0.472 [0.369 - 0.578]	0.438 [0.370 - 0.507]	0.572 [0.481 - 0.658]	0.412 [0.364 - 0.463]	0.469 [0.444 - 0.495]	0.544 [0.511 - 0.577]
-4.5	0.434 [0.342 - 0.532]	0.410 [0.349 - 0.475]	0.555 [0.468 - 0.639]	0.381 [0.339 - 0.426]	0.443 [0.420 - 0.466]	0.530 [0.499 - 0.561]
-4	0.399 [0.317 - 0.488]	0.385 [0.328 - 0.445]	0.538 [0.454 - 0.620]	0.355 [0.317 - 0.396]	0.417 [0.396 - 0.438]	0.517 [0.488 - 0.547]
-3.5	0.367 [0.293 - 0.448]	0.360 [0.308 - 0.416]	0.522 [0.440 - 0.602]	0.333 [0.298 - 0.370]	0.392 [0.373 - 0.411]	0.505 [0.476 - 0.533]
-3	0.339 [0.272 - 0.412]	0.338 [0.289 - 0.390]	0.505 [0.426 - 0.584]	0.314 [0.282 - 0.348]	0.368 [0.350 - 0.385]	0.493 [0.465 - 0.520]
-2.5	0.313 [0.252 - 0.380]	0.316 [0.271 - 0.365]	0.489 [0.413 - 0.567]	0.298 [0.269 - 0.329]	0.345 [0.328 - 0.361]	0.481 [0.454 - 0.508]
-2	0.290 [0.234 - 0.352]	0.297 [0.255 - 0.343]	0.474 [0.399 - 0.550]	0.284 [0.257 - 0.312]	0.323 [0.308 - 0.339]	0.470 [0.444 - 0.496]
-1.5	0.269 [0.218 - 0.327]	0.280 [0.240 - 0.323]	0.459 [0.385 - 0.534]	0.271 [0.246 - 0.297]	0.304 [0.290 - 0.319]	0.459 [0.433 - 0.485]
-1	0.251 [0.204 - 0.305]	0.265 [0.227 - 0.306]	0.445 [0.373 - 0.519]	0.259 [0.235 - 0.284]	0.287 [0.274 - 0.301]	0.448 [0.423 - 0.474]
-0.5	0.235 [0.191 - 0.286]	0.251 [0.215 - 0.291]	0.431 [0.360 - 0.505]	0.248 [0.225 - 0.272]	0.273 [0.260 - 0.286]	0.438 [0.413 - 0.464]
Mean	0.221 [0.179 - 0.269]	0.240 [0.206 - 0.278]	0.418 [0.349 - 0.492]	0.237 [0.216 - 0.260]	0.262 [0.249 - 0.274]	0.429 [0.404 - 0.454]
0.5	0.209 [0.168 - 0.255]	0.231 [0.198 - 0.267]	0.407 [0.338 - 0.480]	0.228 [0.207 - 0.250]	0.253 [0.241 - 0.266]	0.420 [0.395 - 0.446]
1	0.198 [0.159 - 0.244]	0.224 [0.192 - 0.260]	0.397 [0.328 - 0.470]	0.220 [0.200 - 0.241]	0.248 [0.236 - 0.260]	0.412 [0.386 - 0.439]
1.5	0.189 [0.151 - 0.234]	0.219 [0.188 - 0.254]	0.388 [0.319 - 0.462]	0.214 [0.194 - 0.235]	0.245 [0.233 - 0.257]	0.406 [0.379 - 0.433]
2	0.182 [0.145 - 0.226]	0.217 [0.185 - 0.252]	0.380 [0.311 - 0.455]	0.210 [0.190 - 0.231]	0.244 [0.232 - 0.257]	0.400 [0.373 - 0.428]
2.5	0.176 [0.139 - 0.220]	0.216 [0.184 - 0.252]	0.374 [0.304 - 0.450]	0.208 [0.188 - 0.230]	0.245 [0.233 - 0.258]	0.395 [0.368 - 0.424]
3	0.172 [0.135 - 0.216]	0.218 [0.186 - 0.255]	0.369 [0.298 - 0.446]	0.208 [0.187 - 0.231]	0.248 [0.235 - 0.261]	0.392 [0.363 - 0.421]
3.5	0.169 [0.132 - 0.214]	0.223 [0.189 - 0.260]	0.365 [0.293 - 0.444]	0.211 [0.189 - 0.234]	0.251 [0.237 - 0.265]	0.389 [0.359 - 0.420]
4	0.168 [0.130 - 0.215]	0.229 [0.194 - 0.268]	0.363 [0.288 - 0.444]	0.214 [0.192 - 0.238]	0.255 [0.240 - 0.270]	0.387 [0.356 - 0.420]
4.5	0.169 [0.130 - 0.217]	0.238 [0.201 - 0.278]	0.361 [0.285 - 0.445]	0.218 [0.195 - 0.244]	0.259 [0.244 - 0.274]	0.387 [0.353 - 0.421]
5	0.171 [0.130 - 0.221]	0.249 [0.210 - 0.292]	0.361 [0.282 - 0.448]	0.223 [0.197 - 0.251]	0.263 [0.247 - 0.279]	0.387 [0.352 - 0.423]
Age		30-34 yrs			>34 yrs	
Weight gain	Normal weight (n=12,302)	Underweight (n=2,460)	Overweight/Obese (n=4,749)	Normal weight (n=5,750)	Underweight (n=704)	Overweight/Obese (n=3,221)
-5	0.522 [0.467 - 0.577]	0.512 [0.489 - 0.534]	0.599 [0.572 - 0.626]	0.596 [0.508 - 0.677]	0.604 [0.575 - 0.632]	0.666 [0.638 - 0.692]
-4.5	0.489 [0.440 - 0.539]	0.483 [0.462 - 0.504]	0.579 [0.553 - 0.604]	0.561 [0.482 - 0.637]	0.573 [0.546 - 0.600]	0.646 [0.618 - 0.672]
-4	0.458 [0.413 - 0.504]	0.457 [0.438 - 0.476]	0.559 [0.534 - 0.583]	0.527 [0.455 - 0.598]	0.543 [0.517 - 0.569]	0.626 [0.599 - 0.652]
-3.5	0.429 [0.388 - 0.471]	0.433 [0.415 - 0.451]	0.539 [0.515 - 0.562]	0.495 [0.429 - 0.561]	0.514 [0.489 - 0.538]	0.607 [0.581 - 0.633]
-3	0.403 [0.366 - 0.442]	0.411 [0.395 - 0.428]	0.519 [0.496 - 0.542]	0.465 [0.404 - 0.527]	0.486 [0.463 - 0.509]	0.589 [0.563 - 0.615]
-2.5	0.379 [0.345 - 0.415]	0.393 [0.377 - 0.408]	0.501 [0.477 - 0.524]	0.437 [0.380 - 0.497]	0.459 [0.437 - 0.482]	0.573 [0.547 - 0.599]
-2	0.358 [0.326 - 0.391]	0.376 [0.361 - 0.391]	0.483 [0.460 - 0.506]	0.412 [0.357 - 0.469]	0.435 [0.413 - 0.456]	0.558 [0.531 - 0.584]
-1.5	0.339 [0.309 - 0.369]	0.361 [0.347 - 0.376]	0.466 [0.443 - 0.489]	0.389 [0.337 - 0.444]	0.412 [0.391 - 0.434]	0.544 [0.517 - 0.571]
-1	0.321 [0.294 - 0.350]	0.348 [0.334 - 0.362]	0.450 [0.427 - 0.473]	0.369 [0.319 - 0.422]	0.393 [0.373 - 0.414]	0.532 [0.505 - 0.559]
-0.5	0.305 [0.279 - 0.333]	0.337 [0.323 - 0.350]	0.437 [0.414 - 0.460]	0.352 [0.304 - 0.404]	0.377 [0.358 - 0.398]	0.522 [0.494 - 0.549]
Mean	0.291 [0.265 - 0.318]	0.326 [0.313 - 0.340]	0.425 [0.402 - 0.448]	0.338 [0.290 - 0.389]	0.365 [0.345 - 0.386]	0.513 [0.485 - 0.541]
0.5	0.277 [0.252 - 0.304]	0.317 [0.304 - 0.331]	0.415 [0.392 - 0.439]	0.326 [0.278 - 0.377]	0.356 [0.336 - 0.377]	0.506 [0.477 - 0.535]
1	0.265 [0.240 - 0.292]	0.309 [0.296 - 0.323]	0.408 [0.385 - 0.433]	0.317 [0.269 - 0.368]	0.351 [0.330 - 0.372]	0.501 [0.470 - 0.531]
1.5	0.254 [0.230 - 0.281]	0.302 [0.289 - 0.315]	0.404 [0.379 - 0.429]	0.310 [0.263 - 0.361]	0.348 [0.328 - 0.370]	0.496 [0.465 - 0.528]
2	0.245 [0.220 - 0.271]	0.296 [0.282 - 0.309]	0.402 [0.376 - 0.429]	0.306 [0.258 - 0.358]	0.348 [0.327 - 0.370]	0.493 [0.460 - 0.526]
2.5	0.237 [0.212 - 0.264]	0.290 [0.277 - 0.305]	0.403 [0.376 - 0.430]	0.304 [0.256 - 0.357]	0.350 [0.328 - 0.373]	0.491 [0.457 - 0.526]
3	0.231 [0.205 - 0.259]	0.286 [0.272 - 0.301]	0.405 [0.377 - 0.434]	0.304 [0.255 - 0.359]	0.354 [0.330 - 0.378]	0.490 [0.454 - 0.526]
3.5	0.227 [0.200 - 0.256]	0.284 [0.268 - 0.299]	0.410 [0.380 - 0.440]	0.306 [0.255 - 0.363]	0.357 [0.332 - 0.384]	0.490 [0.452 - 0.528]
4	0.224 [0.197 - 0.255]	0.282 [0.266 - 0.298]	0.415 [0.384 - 0.447]	0.310 [0.256 - 0.370]	0.361 [0.334 - 0.389]	0.491 [0.450 - 0.531]
4.5	0.224 [0.195 - 0.257]	0.282 [0.265 - 0.299]	0.422 [0.387 - 0.456]	0.315 [0.257 - 0.379]	0.364 [0.336 - 0.394]	0.492 [0.448 - 0.535]
5	0.226 [0.195 - 0.261]	0.283 [0.265 - 0.302]	0.428 [0.391 - 0.466]	0.321 [0.259 - 0.390]	0.366 [0.336 - 0.398]	0.493 [0.446 - 0.540]
